# Historic changes in species composition for a globally unique bird community

**DOI:** 10.1038/s41598-020-67400-z

**Published:** 2020-07-01

**Authors:** Swen C. Renner, Paul J. J. Bates

**Affiliations:** 1 Ornithology, Natural History Museum Vienna, Burgring 7, 1010 Vienna, Austria; 2 Institute of Zoology, University of Natural Resources and Life Science, Gregor-Mendel-Straße 33/I, 1180 Vienna, Austria; 3 Harrison Institute, Bowerwood House, 15, St Botolph’s Road, Sevenoaks, Kent, TN13 3AQ UK

**Keywords:** Community ecology, Conservation biology, Tropical ecology, Biodiversity, Environmental impact

## Abstract

Significant uncertainties remain of how global change impacts on species richness, relative abundance and species composition. Recently, a discussion emerged on the importance of detecting and understanding long-term fluctuations in species composition and relative abundance and whether deterministic or non-deterministic factors can explain any temporal change. However, currently, one of the main impediments to providing answers to these questions is the relatively short time series of species diversity datasets. Many datasets are limited to 2 years and it is rare for a few decades of data to be available. In addition, long-term data typically has standardization issues from the past and/or the methods are not comparable. We address several of these uncertainties by investigating bird diversity in a globally important mountain ecosystem of the Hkakabo Razi Landscape in northern Myanmar. The study compares bird communities in two periods (pre-1940: 1900–1939 vs. post-2000: 2001–2006). Land-cover classes have been included to provide understanding of their potential role as drivers. While species richness did not change, species composition and relative abundance differed, indicating a significant species turn over and hence temporal change. Only 19.2% of bird species occurred during both periods. Land-cover model predictors explained part of the species richness variability but not relative abundance nor species composition changes. The temporal change is likely caused by minimal methodological differences and partially by land-cover.

## Introduction

Species richness, relative abundance and species composition are dynamic phenomena and vary in space and over time^1,2^. Recorded fluctuations of bird species richness and species diversity are explained by deterministic changes (e.g., global change such as changes in land-cover or land-use intensification^1^), methodological changes (different effort or sites; limited or no standardisation; and methodological and non-systematic errors^3,4^), random processes (e.g., neutral dynamics^5–8^), or any combination of the above.

Global change is rapidly proceeding and includes land-use intensification, changes in land-cover, climate, atmospheric composition, and invasive species, among other factors^1^. Land-cover change is probably the most important in terms of species response^9,10^. It is probably more important than climate change for most biodiversity^1,11^, particularly for many bird species^9^. Land-cover change, including land-use intensification, have been shown to affect species in a variety of direct, indirect and interacting ways, including local extinction, range shifts, changes in local abundance, or interactions with other species^12–14^.

Although several studies have shown the effects of global change in the form of habitat loss or land-use change, these studies typically are limited in explanatory power. In many cases, the historic (previous) baseline, which is used to estimate the diversity statistics, has a low statistical power. In others, the temporal aspect is too short to show meaningful effects^15^. Most studies use only recent baseline data and the time difference (mostly 2 years, in rare instances more than a decade) is too short for changes in species assemblage. Typically they explain only short-term fluctuations, particularly fluctuations within or between consecutive years^7,16,17^.

Only a few regions worldwide remain with a habitat cover of near pristine condition^15^. These untouched areas are embedded in a land-cover mosaic of various forms^18^. The Hkakabo Razi Landscape in the northern tip of Myanmar is largely untouched and includes large tracts (11,280 km^2^) of pristine forests interspersed with a few, relatively small areas of degraded forests or other local land-cover forms^19^. Within this Landscape, an historic bird assemblage has been documented^20^. These baseline data were collected by British and US explorers and include specimens and letters on methods and localities. It is the quality of this historical documentation, together with the rigour of the collecting methodology (which compare favourably and complement the recent efforts), that makes the Hkakabo Razi Landscape almost unique for studying species compositional turnover. At the same time, the effects of land-cover change on birds can be analysed, because the historic samples are located in pristine forests, while our own recent samples cover both pristine and some of the relatively few degraded habitats. The historic and recent collections, when reviewed together, allow for an analysis of the historical changes to bird assemblages, covering data separated by almost 70 years. This will increase our understanding of temporal dynamics in bird communities.

Mountain ecosystems of the tropics are home to high species diversity. Those of the Himalayas, including the Hkakabo Razi Landscape are also home to a rich variety of endemic taxa. For example, the study area hosts at least one endemic bird (*Jabouilleia naungmungensis*^21^) and at least two endemic subspecies of birds^20,22^ (*Alcippe cinereiceps hkakaboraziensis*, *Malacocincla abbotti kachinensis*; all species and samples are listed in Online Supporting Information Table S1). However, all biodiversity in mountain ecosystems is vulnerable to land-cover change^23^. Currently, the forests of the Hkakabo Razi Landscape are likely the last vast area of pristine forests in Asia or at least Southeast Asia^19,24–26^, with relatively few degraded habitats imbedded within the pristine forests. To date, 456 bird species have been recorded in the Hkakabo Razi Landscape, proving its global importance for bird conservation. While the Hkakabo Razi Landscape covers about 1% of terrestrial Myanmar, it is habitat for almost half of all bird species recorded from the country (456 vs. ~ 1,100^20^).

Here we describe and test species turnover and temporal variation in relation to global change parameters. We predict no detectable differences in species richness, relative abundance or species composition between the two periods considered (pre-1940 vs. post-2000), because land-cover change has not yet occurred to a significant extent. In turn, any significant differences in species richness, relative abundance or species composition would indicate a high proportion of temporal variation (i.e. non-static species composition) and/or a response to deterministic reasons (e.g., environmental drivers). If we find a differences in species composition between the two periods of over 50% (natural fluctuations in bird communities of the tropics with very limited human impact exhibit up to 49% species change within or between years^27^), or significant variation between the periods in respect to species richness and relative abundance, these fluctuations can be interpreted as a consequence of temporal change.

Since the Hkakabo Razi Landscape is one of the few remaining significantly large and natural mountain forests worldwide^24–26,28^, from which we have almost perfect historical datasets, it is an invaluable natural laboratory in which to test the impact of temporal change on species richness, relative abundance and species composition. The results of such studies are of global importance. The Hkakabo Razi Landscape is a unique constellation of largely “untouched” forests^26^ with collectors in the first half of the twentieth century having labelled their specimens almost perfectly.

## Material and methods

### Study region and study sites

**Figure 1:**
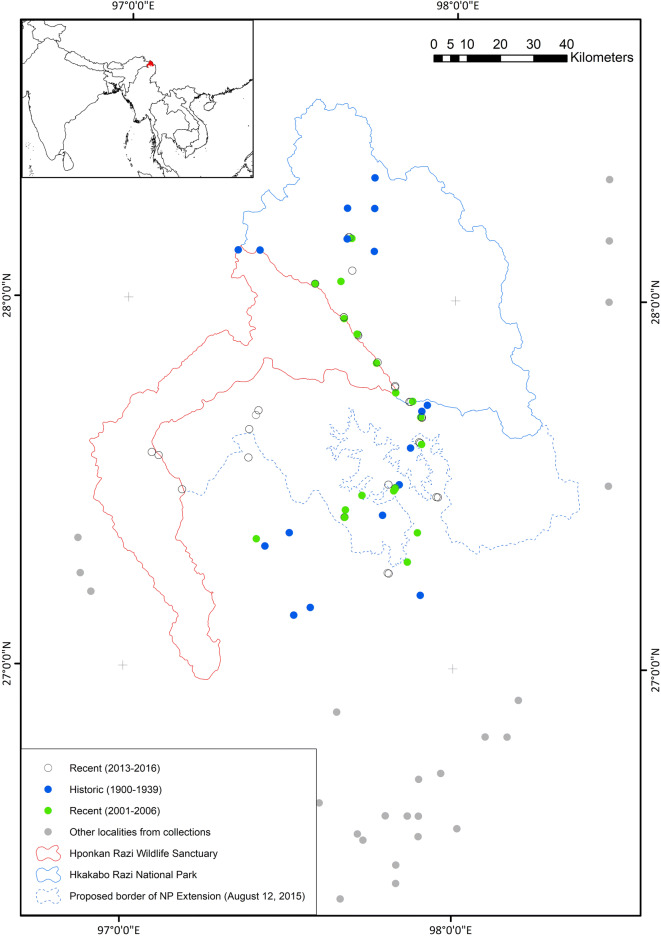
Map of study region in northern Kachin State, Myanmar (*red area* in *inset map* shows the location of the protected areas and outlines the Hkakabo Razi Landscape within Myanmar). *Blue* and *green circles* are for sample sites from which data are used in the study. *Grey* and *open circles* are for sample sites whose data are excluded since they are either outside the study region or have incompatible datasets.

The study sites, i.e. the localities of bird sampling, are located in the Hkakabo Razi Landscape. Distribution of the localities and consequently the area covered is defined by the historic collectors (redrawn in Fig. 1, following Suarez-Rubio, et al.^26^). The Hkakabo Razi Landscape is located in the northern most part of Myanmar (to many Westerners still known as “Burma”) and comprises the Hkakabo Razi National Park, the planned “Southern Extension” of the National Park and the Hponkan Razi Wildlife Sanctuary (all borders as proposed on August 15, 2015).

Within the Hkakabo Razi Landscape, two major survey programmes have been completed to assess the total number of species. The first, a series of uncoordinated ‘one-off’ studies was undertaken by British collectors in the early twentieth century, the second by S.C.R. and several colleagues in the early 2000s^20^. All samples are available, either at the Natural History Museum (Tring, UK), or at the Smithsonian Institution (Washington, DC, USA) or at the Zoological Park (Yangon, Myanmar).

In the post-2000 studies, all samples were taken in accordance with European Union, US and particularly national Myanmar laws on animal protection and conservation measures at the time of data sampling. All necessary permits have been approved by the Nature and Wildlife Conservation Division of Myanmar’s Ministry of Natural Resources and Environmental Protection (MoNREC, formerly Ministry of Forestry—MoF). The responsible officers and the permit number are listed in the acknowledgements.

### Collection based data search

There is an enormous amount of data available from bird collections worldwide. However, insufficient or imprecise locality data and habitat information is an issue for analysis involving museum specimens, particularly if collected prior to the 1960’s. Nevertheless, a quite remarkable number of specimens in the collections is available for the Hkakabo Razi Landscape for further analysis. Those collected by the British forester Ronald Kaulback (sometimes written as Kaulbach) and his colleagues indicate on the labels exact locality, including coordinates and elevation (details listed in Table S1, Online Supporting Information). They also provide simple information about the habitat types and how the birds were captured. Many were collected in the Adung Valley, which is today part of the Hkakabo Razi National Park. Kaulback was loosely associated with Lord Cranbrook, Francis Kingdon-Ward, and Bertram C. Smythies^29–33^, the latter used much of Hebert Cecil Smith’s information from “Notes of the birds of Burma”^34^ in his field guide “Birds of Burma”. Smythies^31^ and Mayr^35^ provided detailed sight records of the birds found by Kaulback, and by Major John K. Stanford^36–50^ and Garthwaite^34^. In addition, the botanist Francis Kingdon-Ward^51–57^ provided some additional specimens to the collections (Kaulback participated in some of Kingdon-Ward’s expeditions). The specimens used for this analysis, were collected by Kaulback (96 specimens), Lord Cranbrook (34), Stanford (30), Kingdon-Ward (12), and an anonymous collector (possibly identified as Kingdon-Ward based on the collection date and locality: 1). All these records are the baseline for reconstruction of the historic bird community used herein.

**Figure 2:**
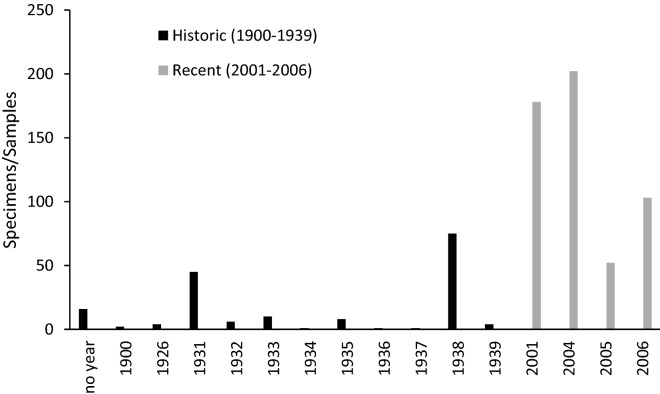
Individuals (specimens) sampled per year in the Hkakabo Razi Landscape pre-1940 and post-2000 with comparable methods and effort.

All the historic specimens are held in the Ornithology Section of the BMNH in Tring, UK (collections visited during the study are listed in “Appendix A”, Online Supporting Information). They were collected between January 1, 1900 and September 20, 1939 (16 specimens are labelled without any date). However, most of the specimens were collected in 1931 and 1938, while the other years show a relatively low coverage (Fig. 2).

Kaulback and colleagues used mainly shotguns in the Hkakabo Razi area and also set snare traps^58^. Kaulback gives a rough indication of his shotgun collecting effort. It is apparent that there was probably two individuals shooting birds for a maximum of “half an hour” each day^58^. The effort for the snare trapping is not documented. However, from the labels written at the time of collection, ~ 5% of the specimens in the Hkakabo Razi Landscape were described as “snared in”. The specimens were collected on a total of 56 capture dates for pre-1940 (dates derived from labels in the collections). This is likely to equate to the number of days Kaulback and his colleagues sampled birds with shotguns or snare traps.

The historic collection covers an area between 27.10 North degree to 28.50 North and 96.50 East to 98.40 East (Fig. 1). The historic spatial extent has been chosen with a maximum overlap with the recent collection in order to maximise the comparison between the two periods. Therefore, we neglect in any analysis further localities of historic and recent collectors, particularly towards the South of the study sites (the so-called “The Triangle”) and east in Yunnan (China) (Fig. 1). The historic sites used in this study, sum up to 17 and have been sampled mainly in February/March (13% of the specimens pre-1940), July–September (34%), November-January (31%) in 1931 and 1938. We verified all data to the best of our knowledge to maximize accuracy and precision.

Only one historic record has been corrected based on inconclusive data (post-museum procedure), because “Adung valley” is certainly not at 97.00 East (as written on specimen label), because 97.00 East is located in India (BMNH 1938.5.5.1, *Strix aluco*, Female, collected by Kingdon-Ward on 7 March 1937). We corrected the locality’s coordinates in our database by using the coordinates as from other specimens’ labels with the same locality name (“Adung Valley”).

### Recent collection and field data accumulation

The post-2000 data were collected between February 9, 2001 and March 20, 2006 (Fig. 1). This dataset comprises collections made by John H. Rappole and S.C.R., with significant support from Thein Aung, Nay Myo Shwe, Myint Kyaw, Myint Aung, and Chris M. Milensky^19–21,59–63^. For all recent sampling included in this study, mist nets of 12 m × 2.6 m have been used (number of capture days and mist nets detailed in Table 1). Typically, nets were set from 05:00 to 10:00 and from 15:00 to 18:00 local time. Recent sites were sampled for 2 days in 2001 and for 1 day each in 2004, 2005, and 2006 (details on capture days and net numbers in Table 1). The sampling season was mainly February to March for the recent sites and there are 65 capture days for the post-2000 period included in this analysis (Table 1).

**Table 1:** Characteristics of the collection trips post-2000.

Year	Dates	Principal investigator	Localities	Collections and/or preparations by	Nets	Capture days	Helpers	References
1997	March–April	Rabinowitz	Putao-Tahaundam	n/a	n/a	n/a	Not acknowledged	^81^
**2001**	**9 February to 12 March**	**Rappole, Renner**	**Putao-Tahaundam**	**Rappole, Renner, Nay Myo Shwe, Kyi Aung, Kyaw Lin**	**15**	**15 (inbound)**	**4 guides/translators, 1 trail boss Tay Za, 1 cook, 60 porters**	^20,59^
					**19**	**15 (outbound)**		
**2004**	**5 to 19 February**	**Rappole, Renner**	**Naung Mung, Nam Ti**	**Rappole, Renner, Nay Myo Shwe, Kyi Aung, A Jo, Tu Myint U, Myint Kyaw**	**25**	**14**	**1 trail boss Aung Khin, 1 cook, 2 cook’s assistants, 15 porters**	^20,59^
**2005**	**7 to 13 September**	**Renner, Myint Aung**	**Naung Mung**	**Myint Aung, Braing Shaw, A Jo**	**10**	**6**	**n/a**	^20,59–62^
**2006**	**5 to 20 March**	**Rappole, Renner**	**Maza, Naung Mung, U Ring Ga**	**Rappole, Renner, Milensky, A Jo, Myint Kyaw**	**20**	**15**	**1 trail boss Aung Khin, 1 cook**	^20,59^
2006	July	Rasmussen	Putao, Nam Ti, Naung Mung, Maza	A Jo, Myint Kyaw, Braing Shaw	10	n/a	1 trail boss Aung Khin, 1 cook	^20,59^
2013/2014	15 December to 30 January	Renner	Putao-Tahaundam	Renner, Sang Nai Dee	10	28	1 cook, 20 helpers	^21,26^
2016	31 January to 27 March	Renner	Wanglaindam, Zyaidam, Mali Raing, Gatu, Gawlei, Shinsanku, Htanga	Renner, Suarez-Rubio, Myint Kyaw, Sang Nai Dee	18	26	1 trail boss Aung Kyaw, 2 cooks, up to 20 helpers	^26^

**Bold** indicates trips with data included for analysis herein.

The methods during historic (snares/shotguns) and current data collections (mist nets) imply differences employed in collection methods. Consequently, some difference based on the methods might explain at least part of the differing species composition (further details in the discussion). However, the datasets are very close—if not identical—for several characteristics: elevational band (between 400 and 2000 m in both periods, with an occasional locality from a higher elevation); capture localities within a small spatial margin (maximum distance between the sites is 25 km; Fig. 1); days of capture (56 vs. 65). The number of sites within the general study area was 17 versus 17. However, the exact localities are different (Fig. 1). The selection criteria for the historic sites is not documented. The recent sites were chosen randomly and are within walking distance of an existing settlement. Historic and recent sites were selected independently from each other.

We use three terms to characterize the bird communities (which are also the dependent variables in the tests as outlined below). **Species richness** is the number of species within the collections. We use the number of species per period (or per land-cover type or per locality) to have a measurement for comparison. With limitations, it is also possible to establish a measurement of abundance for each of the two periods, since all collections show for several species different numbers of sampled individuals per capture site (i.e. the number of samples detected for each species per site). This is described here as **relative abundance**. The number of individuals sampled per species and period is a measurement of abundance. The **species composition** comprises a list of species names per site (or locality or period).

### Land-cover and habitat data

For Hkakabo Razi Landscape, land-cover and land-use classifications are available from 1989^26^, 2001^19^, and 2016^26^. Land-cover has been classified as mostly intact. The changes in land-cover types are marginal with an annual deforestation rate of ≤ 0.23% from 1989 to 2016^26^ (this is indistinguishable from background noise). There has been almost no change in land-cover for the historic bird localities from 1989 to 2016. Only 47 times the land-cover form of the two classifications changed from 1989 to 2016 (Table 2) for the exact localities of the 173 recorded pre-1940 birds (the Landsat 30 m by 30 m pixel^26^). Most of these land-cover changes are negligible and based on melting snow (e.g., from snow/ice/glacier to rock/bolder). Except for the Putao plains, hardly any change occurred from 1989 to 2016^19,26^. The pre-1940 localities have remained undeveloped^58,64,65^ or are close to the same settlements as in 2016. Since most of the habitat remains pristine, all historic (pre-1940) localities are assumed to be of the same land-cover type as classified in 1989. All post-2000 localities are assigned to the land-cover classification as of 2016.

**Table 2:** Changes in land-cover and land-use from 1989 to 2016^26^.

Habitat description 1989	Habitat description 2016	Counts of changes 1989–2016	Remark
Forest > 1800 m	Forest < 600 m	2	–
Forest > 1800 m	Ice/glacier	4	–
Ice/glacier	Rock/boulder	9	Large inter-annual variation possible
Paddy field	Settlement	16	Possibly abandoned rice paddy
Rock/boulder	Pine/Rhododendron	16	–

Habitat classifications in 173 instances have been revised in total.

The land-cover has been classified with the same class names for 1989 and 2016^26^: pine/rhododendron, forest < 600 m, forest 600–1800 m, forest > 1,800 m, grassland/pasture, ice/glacier, clear-cut, paddy field, rock/boulder, secondary forest < 600 m, secondary forest 600–1800 m, settlement, shrub/bush/fern, and streambed.

### Statistical outline

The largest issue for testing is the unknown sampling effort for pre-1940. It is not possible to assess the completeness of the pre-1940 datasets because this information is not available in the archives or from the specimen labels. This makes for some uncertainty when comparing the bird assemblages from the two periods. Nevertheless, we maximized comparability of the datasets, as far as possible with the archival and label information available. Details on methods are further published for post-2000^20,59^.

We performed an ANOVA for species richness and relative abundance to analyse the variance between the periods (pre-1940 vs. post-2000) and used a Kruskal–Wallis test if not normally distributed. In a second step, we added a generalized linear model (GLM) approach to test whether habitat or locality (sampling sites pooled as per nearest settlement name) have an effect on the species richness or relative abundance. Differences between periods and species richness (or relative abundance) were assessed using analysis of variance (ANOVA) after verifying for homogeneity of variances (Fligner test) and normality (Bartlett test). All analyses were performed in R version 3.5.1^66^ and an α-level of 0.05. Observational data (count, species numbers) have been log-transformed (ln).

We assessed differences in bird species composition among periods, habitat types and localities using non-metric multidimensional scaling (NMS). Relative abundance was square-root transformed (vegan-package). The NMS was run using Sørensen (Bray–Curtis) distance with an automatic stepping down resolution starting 200 runs from a random configuration.

Since the number of sample sites and the number of samples overall is relatively low for all analyses, we performed power analysis with the pwr-package in R to assess the strength of statistical outcomes. We assessed power always with a significance level of 0.05. ANOVA (variation of species numbers between periods) had a power of 1; GLM with response “species numbers” 0.137 and power for GLM with response “relative abundance” was 1.

## Results

There were a total of 708 individual bird records belonging to 193 species; 98 species (173 individuals) were only recorded pre-1940 and 132 species (535 individuals) were only recorded post-2000. Only 19.2% (37 species) occurred in both periods. This indicates a considerable discrepancy between the species assemblages. The top five most abundant species differed between the periods: Post-2000, the most abundant species was *Alcippe morrisonia* (42), followed by *Alcippe rufogularis* (25), *Alophoixus flaveolus* (19), *Niltava grandis* (19), and *Ficedula monileger* (18). For pre-1940, the most abundant species was *Garrulax striatus* (7), followed by *Aethopyga saturata* (6), *Heterophasia pulchella* (6), *Arachnothera magna* (4), and *Cissa chinensis* (4) (all samples and species included are listed in Table S1, Online Supporting Information).

**Figure 3:**
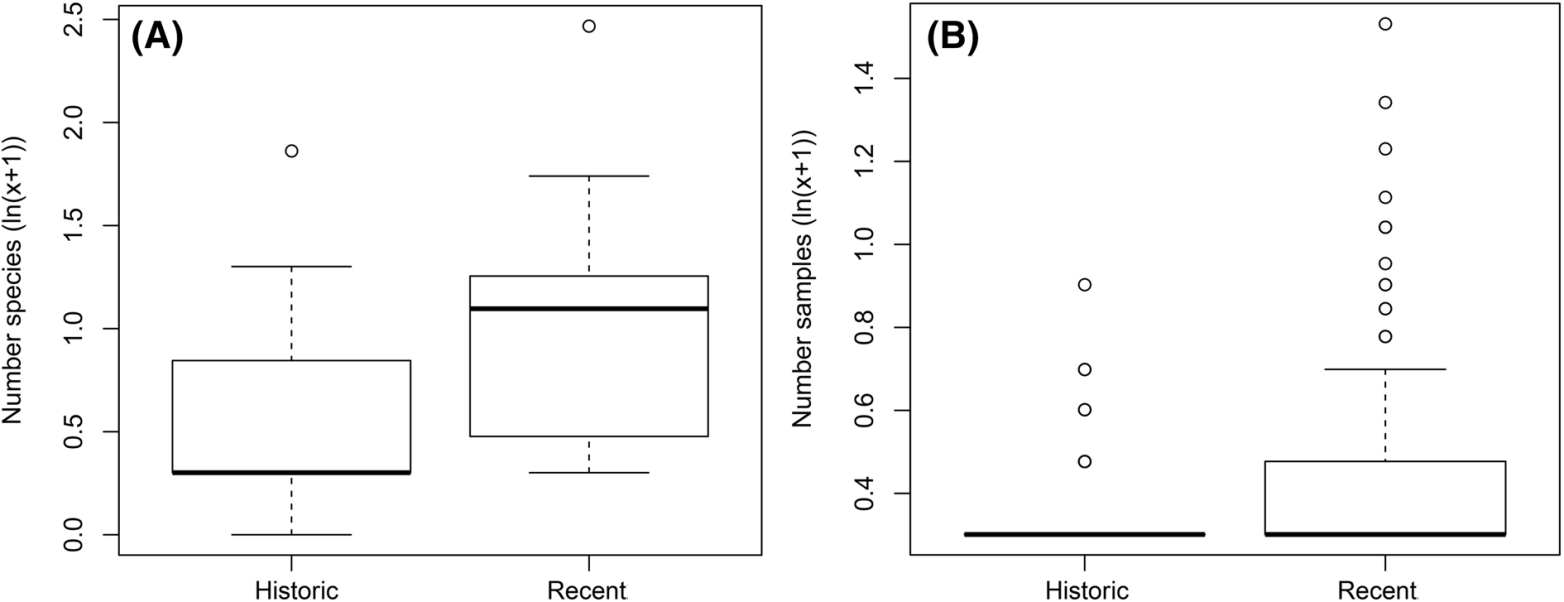
Species numbers (**A**) and relative abundance (**B**) per collecting locality of birds in the Hkakabo Razi Landscape pre-1940 and post-2000 with comparable methods and effort. The *black solid line* indicates the median, *circles* indicate outliers, *whiskers* 95% CI and *box margins* 75% CI.

Analysing species composition with NMS yielded weak ties and hence should be considered with caution. Nevertheless, model-selection procedures in NMS showed that “period” is the best explaining factor out of “period”, “habitat (2016)” and “locality” (CCA stepwise permutation selection *p* for “period” = 0.02, all other *p* > 0.05). Contrasting to the species composition, species richness showed no differences. Species richness did not change from pre-1940 to post-2000 (Kruskal–Wallis χ^2^ = 3.774, *df* = 1, *p* = 0.052; Fig. 3). When modelling species richness with the predictors “locality”, “period”, “land-cover 1989” and “land-cover 2016”, the latter two had an effect on the species richness (*p* < 0.001, GLM models “s2” and “s3” in Table 3). Considering each predictor singly with species richness, only “period” predicts species richness (models “s4” to “s7” in Table 3).

**Table 3:** GLM model results of species richness and abundance of birds in the Hkakabo Razi Landscape pre-1940 and post-2000 with similar methods and effort established.

Model name	Response	Predictor(s)	*DF*	Deviance	Residuals *DF*	Residuals deviance	*p*	AIC
s2	Species richness				38	13.178		35.431
		Locality	25	5.327	13	7.850	0.925	
		Land-cover 1989	8	6.079	5	1.771	**0.022**	
		Period	1	0.408	4	1.363	0.274	
s3	Species richness				38	13.178		7.009
		Locality	25	5.327	13	7.850	0.277	
		Land-cover 2016	8	6.700	5	1.150	** < 0.001**	
		Period	1	0.408	4	0.744	0.138	
s4	Species richness				38	13.178		65.365
		Land-cover 2016	11	5.033	27	8.145	0.118	
s5	Species richness				38	13.178		55.866
		Period	1	2.169	37	11.009	**0.006**	
s6	Species richness				38	13.178		90.102
		Locality	25	5.327	13	7.850	0.999	
s7	Species richness				38	13.178		67.471
		Land-cover 1989	11	4.503	27	8.675	0.232	
a1	Relative abundance				360	12.764		Inf
		Species	191	5.120	169	7.644	0.811	
		Locality	24	3.191	145	4.453	** < 0.001**	
		land-cover 1989	6	0.314	139	4.139	0.099	
		Land-cover 2016	3	0.129	136	4.009	0.223	
		Period	0	0.000	136	4.009	n/a	
a2	Relative abundance				360	12.764		− 249.41
		Locality	25	3.603	335	9.161	** < 0.001**	
		Land-cover 1989	7	0.387	328	8.774	**0.043**	
a3	Relative abundance				361	12.776		− 249.65
		Locality	25	3.615	336	9.161	** < 0.001**	
a4	Relative abundance				360	12.764		− 199.41
		Land-cover 1989	10	1.381	350	11.383	** < 0.001**	
a5	Relative abundance				360	12.764		− 201.03
		Land-cover 2016	10	1.432	350	11.332	** < 0.001**	
a6	Relative abundance				361	12.776		− 200.75
		Period	1	0.804	360	11.973	** < 0.001**	
a7	Relative abundance				361	12.776		18.808
		Species	192	5.132	169	7.644	1.000	

*DF* = degree of freedom, *p* ≤ 0.05 highlighted in **bold**. All estimates are listed in Table S2, Online Supporting Information.

The relative abundance changed from pre-1940 to post-2000 (Kruskal–Wallis χ^2^ = 26.125, *df* = 1, *p* ≤ 0.001; Fig. 3). When modelling relative abundance, neither “land-cover 1989”, “land-cover 2016”, nor “period” had an effect on the relative abundance, but only “locality” (*p* < 0.001, GLM model “a1” in Table 3). Considering each predictor singly with relative abundance, all but “species” predict relative abundance (models “a3” to “a7” in Table 3).

**Figure 4:**
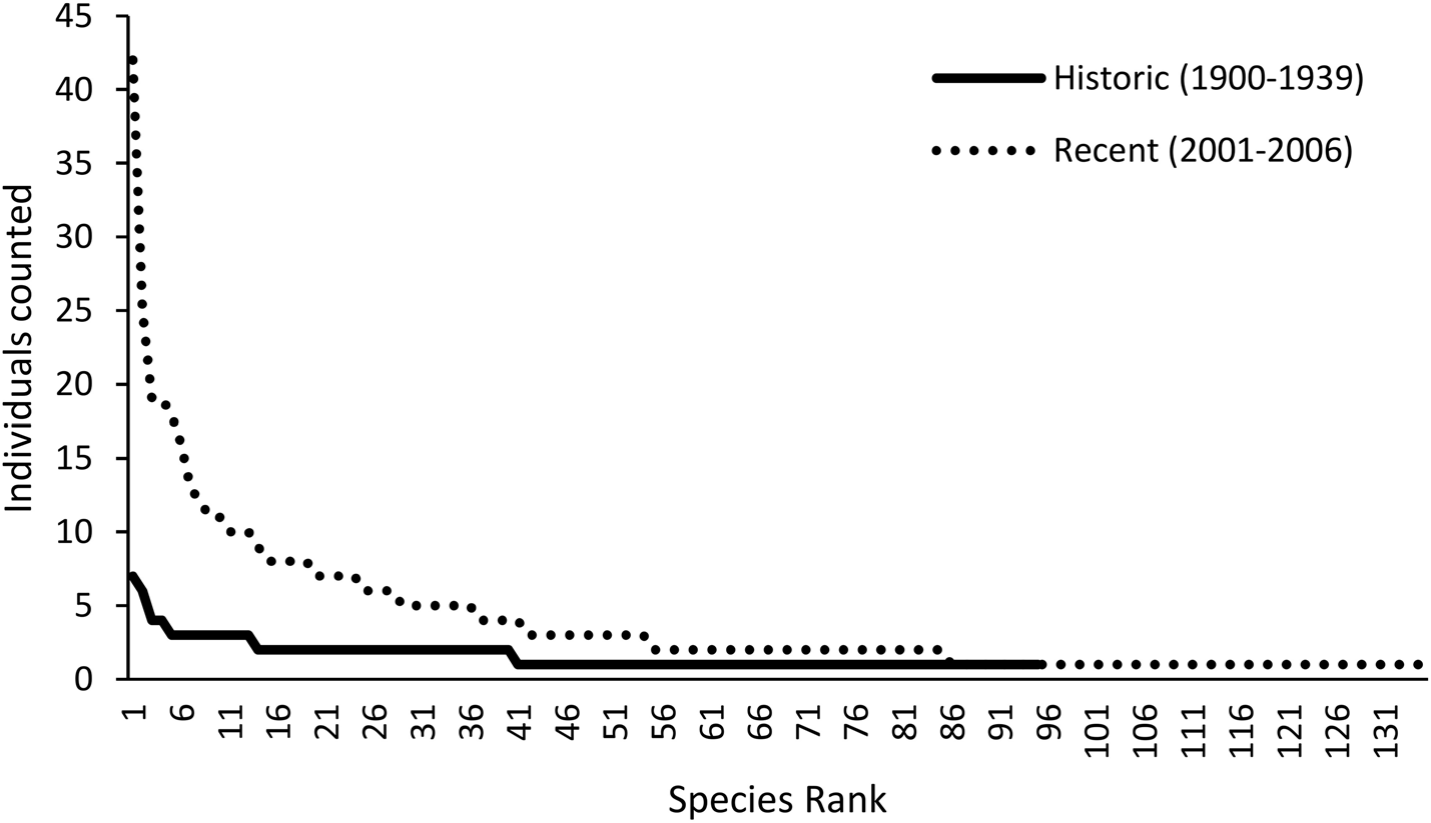
Species rank-abundance (i.e. number of specimens) curve of all detected individuals per period considered in the analysis of birds in the Hkakabo Razi Landscape pre-1940 and post-2000 with comparable methods and effort.

The community structure follows a typical species rank-abundance curve (Fig. 4), with few species of many individuals and many species with few individuals. The species-abundance shows a similar pattern for pre-1940 and post-2000, however, the post-2000 is about one magnitude higher (Fig. 4).

## Discussion

The long-term datasets of Hkakabo Razi Landscape inform us that while species richness did not change from pre-1940 to post-2000, species composition and the relative abundance changed significantly, including the five most abundant species in each period. The magnitude of change in species composition, with less than 20% of taxa shared between the two time periods, is particularly noteworthy and unexpected.

In a world where temporal patterns of biodiversity have received much less attention than spatial ones^67,68^, the datasets from Hkakabo Razi Landscape are important because, almost uniquely, they give us the chance to differentiate between anthropogenic impacts and background temporal changes in ecological communities in an extensive area of Old World forest biome, with a timescale of almost a century (primarily between 1931 and 2006, although a minority of specimens were collected as far back as 1900). The datasets are unusual for such studies because they are based in a subtropical rather than a temperate area and are drawn from a large tract of forest (11,280 km^2^) that remains almost pristine. Furthermore, they are statistically valuable since, although the methodologies between the historical and more recent surveys are not the same, they are well documented and share many comparable components and are sufficiently informative to give us the opportunity to observe temporal changes not only in species diversity and species richness, but also crucially in species composition, and to a lesser extent, relative abundance within species.

The results from Hkakabo Razi Landscape, particularly the large variation in species composition, reflect earlier findings from Costa Rica, where working with more detailed data, albeit gathered over a much shorter time-scale (1985–1992)^67^, researchers noted that tropical bird communities far from being stable systems are in reality dynamic ones with a ‘complex mix of stable and variable components that produce changes in species composition and abundance over various spatial and temporal scales’. This variability in Costa Rica was observed not only, as might be expected, in secondary forest (partially as a response to vegetational succession) but also, though to a lesser extent, in mature forest. It should also be noted that rates of temporal turnover will also vary amongst ecosystem types^67,68^ and in relation to local environmental factors, with variable responses to the same disturbance events^68^.

In contrast to the Hkakabo Razi Landscape study, which provides information on long-term temporal patterns, most others have focused more on short-term fluctuations driven by resource availability^69,70^. These include, for example, the movement of birds in response to the availability of fruits in a mountain biome in Costa Rica; the differential movement of insectivorous and frugivorous birds in Kenya in response to food availability; and the movement of birds in the Australian tropical forest in response to climatic variations and subsequent resource availability^71^. Other studies, both short and long-term, and over a variety of spatial scales, have focused on changes in bird diversity and composition but primarily in areas that have been significantly impacted by anthropogenic activities. These include, for example, studies of the temporal variation of taxonomic and functional diversity in the conterminous USA based on 40 years of data (1970–2011)^72–74^. Such studies, although extremely valuable, do not provide data that enables us to develop conservation policies that take into account purely natural cycles in diversity and abundance.

Without an understanding of natural long-term variability in essentially pristine ecosystems, it is almost impossible to differentiate between human-induced change and natural cycles in those that are anthropogenically modified. As such, the impacts of human induced environmental change may be overstated when comparing differences in species composition at any particular site over a longer time period.

That said, the results of Hkakabo Razi Landscape, should be treated with some caution. For although some of the variables in the collection methods between the pre-1940 and post 2000 datasets are (surprisingly) comparable, others are not. Those that are similar (as outlined in the Methods section) include: elevational band, primarily between 400 and 2000 m in both periods; number of collection sites, 17 versus 17; spatial distribution of capture localities, which although not the same, have a maximum distance between the sites of 25 km (Fig. 1); the number of capture days (56 vs. 65); period of collection 8 years (primarily from 1931 to 1938) and 6 years (from 2001 to 2006)—all of this is important since typically it has been predicted from elsewhere that there will be around twice as many species detected in a decade as in a single year^68^. However, there are also differences, the most important of which is capture method. This could be particularly important in an ecosystem, where it is predicted (based from data collected elsewhere) that high species diversity is inversely correlated to low species density—i.e. many species with fewer individuals. It is probable that some of the difference in species composition observed from the pre-1940 post 2000 data is directly attributable to differences in collecting method. Post-2000, the exclusive use of mist-nets would favour the collection of those bird species that favour niches nearer to ground level, whilst pre-1940, a hunter with a gun, will have greater success with birds, which are more visible and/or high in the canopy. This is reflected in the five most abundant species of pre-1940, which are either more colourful (e.g., brightly coloured such as some laughingtrushes), or easy to watch (such as *Arachnothera magna* which occurs in open forest patches and at the forest edge), or more visible through their behaviour (e.g., loud alarm calls such as from *Garrulax striatus*). Contrasting, the top five post-2000 species are more secretive in behaviour and less bright coloured, hence less obvious to the hunter.

Furthermore, the by one magnitude higher, relative abundance post-2000 is probably a methodological bias. While mist nets capture, for example, the largest part of an *Alcippe morrisonia* flock (20 + individuals, own unpublished observations), the hunters pre-1940 shot one individual out of a flock, and the remainder of the flock certainly escaped and disappeared without trace in the forest. Moreover, mist nets, unlike hunters, do not discriminate since they catch every bird that becomes entangled in them whereas a hunter may either consciously or subconsciously eschew birds of a species for which a number of specimens have already been collected. Theoretically, the only way to compare the relative abundance between the two periods would be to collect birds today in a manner similar to that employed pre-1940. However, these methods, shooting and snares, are obviously not possible or desirable today for ethical reasons and Myanmar national laws.

In addition to variation in capture methods, there is some variation in the season of capture between the two datasets. Post-2000, all 535 individuals of the 132 species were collected in the months February–March. However, for the 173 specimens collected from pre-1940, 13% were captured in the February–March time period whilst the remainder were collected mainly in July–September and November-January. This is important since Myanmar hosts a diverse winter migrant bird fauna and since inter-seasonal fluctuations in bird composition are known to be on average higher for migratory and nomadic species than for sedentary ones^70,75,76^. However, interestingly, hardly any long-distance migrants were detected in either the pre-1940 or post-2000 datasets so that migration status alone cannot explain the large fluctuations seen in species composition between the two time periods.

An additional analysis including, for instance, the phylogenetic structure^77^ of the bird community or its functional traits, could add further insights. However, for this paper we have avoided such approaches since, currently, the phylogenetic structure of the phylogenetic placement and validity of the three most important families in our data set, the Muscicapidae, Timaliidae, and Pellorneidae, are controversial and all deep-phylogeny assignments are in continuous flow for many species occurring in the Hkakabo Razi Landscape (detailed in Online Supporting Information C). Meanwhile data on the functional traits of bird species from Hkakabo Razi Landscape remains incomplete and/or speculative with little detailed information on the functional groups beyond generalised descriptions, such as insectivores, granivores,… There are also no data available on seasonal variation, e.g. breeding versus non-breeding^18^. Therefore, rather than working with incomplete or speculative data sets, we focused on the parsimonious and relative robust analysis of the bird community.

Preliminary analysis of the long-term Hkakabo Razi Landscape datasets provide some very interesting information that is of importance not just to bird ecologists but to the much broader scientific community, especially those concerned with environmental change, including climate change and habitat fragmentation, and its impact on biodiversity. The datasets help put short-term fluctuations into a meaningful context, for example within monitoring programmes, and provide information that gives an insight into whether contemporary trends in diversity are simply a response to anthropogenic-induced changes or are the result of dynamics originating before the onset of the Anthropocene^78,79^. They also have important implications for conservationists who seek to interpret the meanings of changes in faunal composition both in natural and man-made habitats and who wish to develop conservation policies that take into account natural cycles in diversity and abundance. As with interesting studies in the USA and France, the next stage for the Hkakabo Razi Landscape data are to develop more sophisticated models to determine if significant changes in taxonomic diversity are also reflected in changes in phylogenetic and functional diversities^79,80^, as well as determining random portion of the species richness^5–8^.

## Conclusion

The two Hkakabo Razi Landscape datasets, pre-1940 and post-2000, give an invaluable insight into the question ‘what is the underlying level of temporal turnover in a bird community?’ They help us to understand background turnover in birds in a subtropical pristine forest site, which will provide an invaluable foundation (despite the caveat of different methodologies in the two datasets) when trying to assess anthropogenic impacts in increasingly disturbed habitats elsewhere. The datasets further challenge the notion that bird communities in the tropics/subtropics, even in natural habitats, are stable systems. Rather they show that there is an important temporal component to biodiversity and that natural ecosystems are dynamic with a complex combination of stable and variable components and that this dynamic component impacts in different ways and with different severity on species diversity, species composition, and relative abundance.

## Supplementary information

Supplementary information

## Data Availability

The data used for analysis is available in the Online Supporting Information.
