# Supplementary material for: Historic changes in species composition for a globally unique bird community
Source: Sci Rep. 2020 Jul 1;10:10739. doi: 10.1038/s41598-020-67400-z (PMC7329821; doi:10.1038/s41598-020-67400-z)
Supplement: Supplementary file 1 — Supplementary information [file 41598_2020_67400_MOESM1_ESM.docx]

Historic changes in species composition for a globally unique bird community

Swen C. Renner and Paul J. J. Bates

# Description of Supplementary Information

**Appendix A.** Collections visited

**Appendix B.** Climate data and further explanations of data reliability and precision

**Appendix C.** Preliminary analysis beyond taxonomic diversity

**Table S1.** List of data entries based on collections as used in the analysis.

**Table S2.** Model estimates of GLM.

**Figure S1.** Residuals for species numbers **(A)** and relative abundance **(B)** of birds in the Hkakabo Razi Landscape pre-1940 and post-2000 with similar methods and effort established.

**Figure S2.** Density of bird records in respect of elevation of 365 species known from the Hkakabo Razi Landscape.

**Figure S3.** Families after “The Tree of Live” (alphabetical order) of the species encountered pre-1940 and post-2000 in the Hkakabo Razi Landscape.

**References cited** in Online Supporting Information

# Appendix A. Collections visited

The following museums have been visited during the course of the study since 2001. However, most of the databased specimens from all of the museums have limited relevance for the present study because of limited geographic precision or incomplete datasets.

- AMNH – American Museum of Natural History, New York, USA
- ANSP – Academy of Natural Sciences, Philadelphia, USA
- BMNH – British Museum of Natural History, Bird Collection, Tring, UK
- CAS – California Academy of Sciences, San Francisco, USA
- MfN – Natural History Museum (*Museum für Naturkunde*), Berlin, Germany
- NMN – Naturalis Natural History Museum, Leiden, The Netherlands
- NMNH (USNM) – Smithsonian’s National Museum of Natural History, Washington DC, USA
- NHMW – Natural History Collections (*Naturhistorisches Museum Wien*), Vienna, Austria
- NRM – Natural History Museum, Stockholm, Sweden
- SMNS – State Museum of Natural History, Stuttgart, Germany
- ZMUC – Zoological University Museum, Copenhagen, Denmark

# Appendix B. Climate data and further explanations of data reliability and precision

Climatic data for the Hkakabo Razi Landscape are available, however, only for one point at the Putao weather station since the mid-1960’s (exact date not verified). Beside the data, only extrapolations and models from elsewhere could support our analysis. Data on climate is hence in general available as monthly mean temperature and monthly mean precipitation; however, the accuracy and precision of the data are not known. Other studies use weather stations in relative geographic proximity, but rely on extrapolation methods which are very rough (50 km grid cells)^1^. The so-called ‘Myanmar Gazetteers’ sometimes provide weather records for the British colonial period, but only for Myitkyina (approximately 220 km South of Putao) or other places in Myanmar^2-15^.

One climate change observation (in the form of an extrapolation over a large scale) indicates a decrease of mean annual precipitation by 40% from 1900 to 2000 in the eastern Himalayas^16^. This trend is predicted to continue; a predicted increase in temperature of 4°C by the end of the current century is possible (SRES scenario A2 IPCC 2002, cf. Renner, et al. ^17^).

# Appendix C. Preliminary analysis beyond taxonomic diversity

In addition to the analysis of taxonomic diversity, we also considered two other approaches, phylogenetic structure and functional traits. However, we decided to exclude a phylogenetic analysis from our specific framework because the phylogenetic placement and validity of the Muscicapidae, Timaliidae, and Pellorneidae are controversial and all deep-phylogeny assignments are in continuous flow for many species occurring in the Hkakabo Razi Landscape. Since these are the three most important families in our data set (Fig. S3), we felt it was premature to include this aspect into our statistical analysis. Second, increasing the levels or number of groups would decrease significantly the robustness of our statistical tests (power analysis, see method section in main text).

Nevertheless, we did undertake a surrogate-analysis for phylogenetic diversity based on the familial structure outlined in “The Tree of Life” (Fig. S3). This analysis can be regarded as a first approximation of a phylogenetic study but includes some limitations, as outlined above. We found:

- All species are placed in 45 families.
- Most families are represented by species from both periods (Fig. S3).
- Three families are overrepresented post-2000 compared to pre-1940: the Muscicapidae, Timaliidae, Pellorneidae – we suggest that species in these three families are relatively shy and that they are likely driven away by shotguns/hunting, but not by passive/silent mist netting methods.

As a first statistical approach, we performed a t-test to compare the families in the two datasets (2-sided, different variance); the families are not significantly different in presence pre-1940 to post-2000 (t-test *p*=0.17 ±3.81 s.d. pre-1940 and ±10.25 post-2000; variance: 14.21 pre-1940, 102.78 post-2000). This indicates that the bird families of the bird community of the two datasets are not differing.

An alternative to analysing the phylogenetic structure would be to analyse the functional traits of the bird species. This approach also has limitations for many species in Myanmar and particularly for the Hkakabo Razi Landscape, since (a) there is little detailed information on the functional groups beyond generalised descriptions, such as insectivores, granivores,…; (b) much of the information is based on speculation rather than observed behaviour and (c) no data are available on seasonal variation, e.g. breeding vs. non-breeding^18^.

Since the data for functional or phylogenetic analysis was either not available or of low quality and/or insufficient in quantity, it was considered better to omit any such hypothetical analysis and focus on taxonomic diversity, for which we had extensive, reliable data.

**Table** **S1.** List of data entries based on collections as used in the analysis. *Coordinates* indicate the source of the coordinates as listed. *Type* indicates pre-1940 (covering the years 1900 to 1939 = historic) *vs.* post-2000 (covering the years 2001 to 2006 = recent and own data); “settlement” indicates sampling in the proximity to settlements, not necessarily sampling in the village.

| **ID** | ***Species*** | **Coordinates** | **North** | **East** | **Collector** | **Type** | **year** | **Habitat^19^** |
| --- | --- | --- | --- | --- | --- | --- | --- | --- |
| 5086 | *Polyplectron bicalcaratum* | ^16^ | 27.14 | 97.52 | Stanford | Pre-1940 | n/a | Forest 600-1800m |
| 2999 | *Alophoixus flaveolus* | label | 27.7 | 97.9 | Kaulback | Pre-1940 | n/a | Forest 600-1800m |
| 2800 | *Leiothrix argentauris* | label | 27.7 | 97.9 | Kaulback | Pre-1940 | n/a | Forest 600-1800m |
| 4782 | *Aceros undulatus* | label | 28.13 | 97.4 | Kaulback | Pre-1940 | n/a | Pine/Rhododendron |
| 3760 | *Aethopyga saturata* | label | 28.13 | 97.4 | Kaulback | Pre-1940 | n/a | Pine/Rhododendron |
| 3761 | *Aethopyga saturata* | label | 28.13 | 97.4 | Kaulback | Pre-1940 | n/a | Pine/Rhododendron |
| 3762 | *Aethopyga saturata* | label | 28.13 | 97.4 | Kaulback | Pre-1940 | n/a | Pine/Rhododendron |
| 3763 | *Aethopyga saturata* | label | 28.13 | 97.4 | Kaulback | Pre-1940 | n/a | Pine/Rhododendron |
| 4650 | *Cissa chinensis* | label | 28.13 | 97.4 | Kaulback | Pre-1940 | n/a | Pine/Rhododendron |
| 4652 | *Cissa chinensis* | label | 28.13 | 97.4 | Kaulback | Pre-1940 | n/a | Pine/Rhododendron |
| 2202 | *Garrulax ruficollis* | label | 28.13 | 97.4 | Kaulback | Pre-1940 | n/a | Pine/Rhododendron |
| 4844 | *Nyctyornis athertoni* | label | 28.13 | 97.4 | Kaulback | Pre-1940 | n/a | Pine/Rhododendron |
| 4659 | *Oriolus traillii* | label | 28.13 | 97.4 | Kaulback | Pre-1940 | n/a | Pine/Rhododendron |
| 3385 | *Paradoxornis gularis* | label | 28.13 | 97.4 | Kaulback | Pre-1940 | n/a | Pine/Rhododendron |
| 3668 | *Parus monticolus* | label | 28.13 | 97.4 | Kaulback | Pre-1940 | n/a | Pine/Rhododendron |
| 3349 | *Yuhina flavicollis* | label | 28.13 | 97.4 | Kaulback | Pre-1940 | n/a | Pine/Rhododendron |
| 2247 | *Garrulax gularis* | GoogleEarth | 27.33 | 97.43 | Stanford | Pre-1940 | 1900 | Settlement |
| 2248 | *Garrulax gularis* | GoogleEarth | 27.33 | 97.43 | Stanford | Pre-1940 | 1900 | Settlement |
| 5057 | *Ithaginis cruentus* | label | 28.13 | 97.4 | Kingdon-Ward | Pre-1940 | 1926 | Pine/Rhododendron |
| 2297 | *Garrulax affinis* | label | 28.13 | 97.4 | Kingdon-Ward | Pre-1940 | 1926 | Pine/Rhododendron |
| 5054 | *Ithaginis cruentus* | label | 28.13 | 97.13 | Kingdon-Ward | Pre-1940 | 1926 | Ice/glacier |
| 5056 | *Ithaginis cruentus* | label | 28.13 | 97.13 | Kingdon-Ward | Pre-1940 | 1926 | Ice/glacier |
| 3414 | *Abroscopus schisticeps* | label | 28.16 | 97.67 | Cranbrook | Pre-1940 | 1931 | Forest > 1800m |
| 3622 | *Aegithalos iouschistos* | label | 28.16 | 97.66 | Cranbrook | Pre-1940 | 1931 | Forest > 1800m |
| 4721 | *Aethopyga gouldiae* | label | 28.17 | 97.66 | Kingdon-Ward | Pre-1940 | 1931 | Forest > 1800m |
| 4736 | *Aethopyga nipalensis* | label | 28.16 | 97.66 | Cranbrook | Pre-1940 | 1931 | Forest > 1800m |
| 3429 | *Alcippe vinipectus* | label | 28.16 | 97.66 | Cranbrook | Pre-1940 | 1931 | Forest <600m |
| 1533 | *Chaimarrornis leucocephalus* | label | 28.13 | 97.75 | Cranbrook | Pre-1940 | 1931 | Rock/boulder |
| 4201 | *Collocalia brevirostris* | label | 28.17 | 97.67 | Cranbrook | Pre-1940 | 1931 | Forest > 1800m |
| 4677 | *Corvus macrorhynchus* | label | 28.17 | 97.66 | Cranbrook | Pre-1940 | 1931 | Forest > 1800m |
| 3564 | *Culicicapa ceylonensis* | label | 28.16 | 97.66 | Cranbrook | Pre-1940 | 1931 | Ice/glacier |
| 4926 | *Dendrocopus cathparius* | label | 28.17 | 97.66 | Cranbrook | Pre-1940 | 1931 | Forest > 1800m |
| 3467 | *Ficedula sapphira* | label | 28.16 | 97.66 | Cranbrook | Pre-1940 | 1931 | Forest <600m |
| 3421 | *Ficedula strophiata* | label | 28.16 | 97.66 | Cranbrook | Pre-1940 | 1931 | Ice/glacier |
| 2295 | *Garrulax affinis* | label | 28.13 | 97.75 | Kaulback | Pre-1940 | 1931 | Rock/boulder |
| 2296 | *Garrulax affinis* | label | 28.17 | 97.67 | Cranbrook | Pre-1940 | 1931 | Forest > 1800m |
| 2743 | *Garrulax erythrocephalus* | label | 28.17 | 97.67 | Kingdon-Ward | Pre-1940 | 1931 | Forest > 1800m |
| 2279 | *Garrulax subunicolor* | label | 28.13 | 97.75 | Kingdon-Ward | Pre-1940 | 1931 | Rock/boulder |
| 1028 | *Heterophasia pulchella* | label | 28.16 | 97.67 | Kingdon-Ward | Pre-1940 | 1931 | Ice/glacier |
| 2956 | *Hypsipetes mcclelandi* | label | 28.17 | 97.67 | Cranbrook | Pre-1940 | 1931 | Forest > 1800m |
| 2825 | *Leiothrix lutea* | label | 28.17 | 97.66 | Cranbrook | Pre-1940 | 1931 | Forest > 1800m |
| 4401 | *Leucosticte nemoricola* | label | 28.17 | 97.66 | Cranbrook | Pre-1940 | 1931 | Forest > 1800m |
| 3559 | *Muscicapa sibirica* | label | 28.13 | 97.75 | Cranbrook | Pre-1940 | 1931 | Rock/boulder |
| 4550 | *Mycerobas affinis* | label | 28.13 | 97.75 | Cranbrook | Pre-1940 | 1931 | Rock/boulder |
| 4557 | *Mycerobas carnipes* | label | 28.13 | 97.75 | Cranbrook | Pre-1940 | 1931 | Rock/boulder |
| 3629 | *Parus ater* | label | 28.25 | 97.75 | Cranbrook | Pre-1940 | 1931 | Pine/Rhododendron |
| 3828 | *Parus ater* | label | 28.13 | 97.75 | Cranbrook | Pre-1940 | 1931 | Rock/boulder |
| 3667 | *Parus monticolus* | label | 28.16 | 97.66 | Cranbrook | Pre-1940 | 1931 | Forest > 1800m |
| 4488 | *Passer rutilans* | label | 28.16 | 97.67 | Cranbrook | Pre-1940 | 1931 | Forest > 1800m |
| 3171 | *Pericrocotus ethologus* | label | 28.25 | 97.66 | Cranbrook | Pre-1940 | 1931 | Rock/boulder |
| 5026 | *Pericrocotus ethologus* | label | 28.25 | 97.67 | Cranbrook | Pre-1940 | 1931 | Rock/boulder |
| 1403 | *Phoenicurus frontalis* | label | 28.16 | 97.66 | Cranbrook | Pre-1940 | 1931 | n/a |
| 3408 | *Phylloscopus inornatus* | label | 28.13 | 97.75 | Cranbrook | Pre-1940 | 1931 | Rock/boulder |
| 3410 | *Phylloscopus magnirostris* | label | 28.25 | 97.75 | Cranbrook | Pre-1940 | 1931 | Pine/Rhododendron |
| 1117 | *Prunella immaculata* | label | 28.16 | 97.66 | Cranbrook | Pre-1940 | 1931 | Ice/glacier |
| 3052 | *Pycnonotus xanthorrous* | label | 28.16 | 97.67 | Kingdon-Ward | Pre-1940 | 1931 | Forest > 1800m |
| 4551 | *Pyrrhula erythaca* | label | 28.13 | 97.75 | Cranbrook | Pre-1940 | 1931 | Rock/boulder |
| 4390 | *Pyrrhula nipalensis* | label | 28.16 | 97.66 | Cranbrook | Pre-1940 | 1931 | Forest > 1800m |
| 5060 | *Trogopan temminckii* | label | 28.17 | 97.67 | Kingdon-Ward | Pre-1940 | 1931 | Forest > 1800m |
| 4786 | *Upupa epops* | label | 28.16 | 97.67 | Kaulback | Pre-1940 | 1931 | Forest > 1800m |
| 3350 | *Yuhina flavicollis* | label | 28.17 | 97.67 | Cranbrook | Pre-1940 | 1931 | Forest > 1800m |
| 3304 | *Yuhina gularis* | label | 28.17 | 97.66 | Cranbrook | Pre-1940 | 1931 | Forest > 1800m |
| 2168 | *Garrulax striatus* | label | 27.7 | 97.9 | Kingdon-Ward | Pre-1940 | 1931 | Forest 600-1800m |
| 5165 | *Glaucidium cuculoides* | label | 27.7 | 97.9 | Cranbrook | Pre-1940 | 1931 | Forest 600-1800m |
| 1016 | *Heterophasia pulchella* | label | 27.7 | 97.9 | n/a | Pre-1940 | 1931 | Forest 600-1800m |
| 5002 | *Pericrocotus flammeus* | label | 27.7 | 97.9 | Cranbrook | Pre-1940 | 1931 | Forest 600-1800m |
| 4679 | *Arachnothera longirostris* | label | 28.13 | 97.4 | Stanford | Pre-1940 | 1931 | Pine/Rhododendron |
| 4997 | *Anthus roseatus* | ^16^ | 27.14 | 97.52 | Stanford | Pre-1940 | 1932 | Forest 600-1800m |
| 2249 | *Garrulax gularis* | ^16^ | 27.14 | 97.52 | Stanford | Pre-1940 | 1932 | Forest 600-1800m |
| 2200 | *Garrulax ruficollis* | ^16^ | 27.14 | 97.52 | Stanford | Pre-1940 | 1932 | Forest 600-1800m |
| 2205 | *Garrulax ruficollis* | ^16^ | 27.14 | 97.52 | Stanford | Pre-1940 | 1932 | Forest 600-1800m |
| 3338 | *Yuhina nigrimenta* | ^16^ | 27.14 | 97.52 | Stanford | Pre-1940 | 1932 | Forest 600-1800m |
| 4681 | *Arachnothera longirostris* | GoogleEarth | 27.33 | 97.43 | Stanford | Pre-1940 | 1932 | Settlement |
| 3669 | *Parus monticolus* | ^16^ | 27.14 | 97.52 | Stanford | Pre-1940 | 1933 | Forest 600-1800m |
| 4826 | *Ceryle lugubris* | ^16^ | 27.16 | 97.57 | Stanford | Pre-1940 | 1933 | Streambed |
| 4660 | *Dendrocitta frontalis* | ^16^ | 27.16 | 97.57 | Stanford | Pre-1940 | 1933 | Streambed |
| 4668 | *Dendrocitta frontalis* | ^16^ | 27.16 | 97.57 | Stanford | Pre-1940 | 1933 | Streambed |
| 5159 | *Glaucidium brodiei* | ^16^ | 27.16 | 97.57 | Stanford | Pre-1940 | 1933 | Streambed |
| 5036 | *Hemipus picatus* | ^16^ | 27.16 | 97.57 | Stanford | Pre-1940 | 1933 | Streambed |
| 4672 | *Acridotheres tristes* | GoogleEarth | 27.33 | 97.43 | Stanford | Pre-1940 | 1933 | Settlement |
| 4827 | *Ceryle lugubris* | GoogleEarth | 27.33 | 97.43 | Stanford | Pre-1940 | 1933 | Settlement |
| 947 | *Lanius schach* | GoogleEarth | 27.33 | 97.43 | Stanford | Pre-1940 | 1933 | Settlement |
| 4972 | *Psarisomus dalhousiae* | GoogleEarth | 27.33 | 97.43 | Stanford | Pre-1940 | 1933 | Settlement |
| 2210 | *Garrulax nuchalis* | GoogleEarth | 27.33 | 97.43 | Stanford | Pre-1940 | 1934 | Settlement |
| 4990 | *Anthus richardi* | GoogleEarth | 27.33 | 97.43 | Stanford | Pre-1940 | 1935 | Settlement |
| 4930 | *Blythipicus pyrrhotis* | GoogleEarth | 27.33 | 97.43 | Stanford | Pre-1940 | 1935 | Settlement |
| 4653 | *Cissa chinensis* | GoogleEarth | 27.33 | 97.43 | Stanford | Pre-1940 | 1935 | Settlement |
| 4667 | *Dendrocitta frontalis* | GoogleEarth | 27.33 | 97.43 | Stanford | Pre-1940 | 1935 | Settlement |
| 2080 | *Garrulax monileger* | GoogleEarth | 27.33 | 97.43 | Stanford | Pre-1940 | 1935 | Settlement |
| 2203 | *Garrulax ruficollis* | GoogleEarth | 27.33 | 97.43 | Stanford | Pre-1940 | 1935 | Settlement |
| 4838 | *Nyctyornis athertoni* | GoogleEarth | 27.33 | 97.43 | Stanford | Pre-1940 | 1935 | Settlement |
| 4658 | *Oriolus traillii* | GoogleEarth | 27.33 | 97.43 | Stanford | Pre-1940 | 1935 | Settlement |
| 2215 | *Garrulax nuchalis* | label | 27.7 | 97.9 | Stanford | Pre-1940 | 1936 | Forest 600-1800m |
| 5157 | *Strix aluco* | label | 28.25 | 97.67 | Kingdon-Ward | Pre-1940 | 1937 | Rock/boulder |
| 3106 | *Pycnonotus jocosus* | label | 27.37 | 97.5 | Kaulback | Pre-1940 | 1938 | Secondary forest <600m |
| 3110 | *Pycnonotus jocosus* | label | 27.37 | 97.5 | Kaulback | Pre-1940 | 1938 | Secondary forest <600m |
| 1953 | *Sphenocichla roberti* | label | 27.5 | 97.83 | Kaulback | Pre-1940 | 1938 | Shrub/Bush/Fern |
| 4774 | *Aceros nipalensis* | label | 27.7 | 97.9 | Kaulback | Pre-1940 | 1938 | Forest 600-1800m |
| 3196 | *Actinodura egertoni* | label | 27.7 | 97.9 | Kaulback | Pre-1940 | 1938 | Forest 600-1800m |
| 3758 | *Aethopyga saturata* | label | 27.7 | 97.9 | Kaulback | Pre-1940 | 1938 | Forest 600-1800m |
| 3759 | *Aethopyga saturata* | label | 27.7 | 97.9 | Kaulback | Pre-1940 | 1938 | Forest 600-1800m |
| 1418 | *Alcippe cinerea* | label | 27.7 | 97.9 | Kaulback | Pre-1940 | 1938 | Forest 600-1800m |
| 1419 | *Alcippe cinerea* | label | 27.7 | 97.9 | Kaulback | Pre-1940 | 1938 | Forest 600-1800m |
| 4989 | *Anthus richardi* | label | 27.7 | 97.9 | Kaulback | Pre-1940 | 1938 | Forest 600-1800m |
| 3805 | *Arachnothera magna* | label | 27.7 | 97.9 | Kaulback | Pre-1940 | 1938 | Forest 600-1800m |
| 3806 | *Arachnothera magna* | label | 27.7 | 97.9 | Kaulback | Pre-1940 | 1938 | Forest 600-1800m |
| 3807 | *Arachnothera magna* | label | 27.7 | 97.9 | Kaulback | Pre-1940 | 1938 | Forest 600-1800m |
| 3808 | *Arachnothera magna* | label | 27.7 | 97.9 | Kaulback | Pre-1940 | 1938 | Forest 600-1800m |
| 4927 | *Blythipicus pyrrhotis* | label | 27.7 | 97.9 | Kaulback | Pre-1940 | 1938 | Forest 600-1800m |
| 3631 | *Cheliodorhynx hypoxantha* | label | 27.7 | 97.9 | Kaulback | Pre-1940 | 1938 | Forest 600-1800m |
| 4649 | *Cissa chinensis* | label | 27.7 | 97.9 | Kaulback | Pre-1940 | 1938 | Forest 600-1800m |
| 4203 | *Collocalia brevirostris* | label | 27.7 | 97.9 | Kaulback | Pre-1940 | 1938 | Forest 600-1800m |
| 4204 | *Collocalia brevirostris* | label | 27.7 | 97.9 | Kaulback | Pre-1940 | 1938 | Forest 600-1800m |
| 3078 | *Coracina melaschistos* | label | 27.7 | 97.9 | Kaulback | Pre-1940 | 1938 | Forest 600-1800m |
| 5009 | *Coracina melaschistos* | label | 27.7 | 97.9 | Kaulback | Pre-1940 | 1938 | Forest 600-1800m |
| 3181 | *Delichon dasypus* | label | 27.7 | 97.9 | Kaulback | Pre-1940 | 1938 | Forest 600-1800m |
| 4799 | *Eurystomus orientalis* | label | 27.7 | 97.9 | Kaulback | Pre-1940 | 1938 | Forest 600-1800m |
| 4800 | *Eurystomus orientalis* | label | 27.7 | 97.9 | Kaulback | Pre-1940 | 1938 | Forest 600-1800m |
| 4801 | *Eurystomus orientalis* | label | 27.7 | 97.9 | Kaulback | Pre-1940 | 1938 | Forest 600-1800m |
| 2725 | *Garrulax erythrocephalus* | label | 27.7 | 97.9 | Kaulback | Pre-1940 | 1938 | Forest 600-1800m |
| 2167 | *Garrulax striatus* | label | 27.7 | 97.9 | Kaulback | Pre-1940 | 1938 | Forest 600-1800m |
| 2169 | *Garrulax striatus* | label | 27.7 | 97.9 | Kaulback | Pre-1940 | 1938 | Forest 600-1800m |
| 2170 | *Garrulax striatus* | label | 27.7 | 97.9 | Kaulback | Pre-1940 | 1938 | Forest 600-1800m |
| 2171 | *Garrulax striatus* | label | 27.7 | 97.9 | Kaulback | Pre-1940 | 1938 | Forest 600-1800m |
| 2172 | *Garrulax striatus* | label | 27.7 | 97.9 | Kaulback | Pre-1940 | 1938 | Forest 600-1800m |
| 2173 | *Garrulax striatus* | label | 27.7 | 97.9 | Kaulback | Pre-1940 | 1938 | Forest 600-1800m |
| 2930 | *Hemixos flavala* | label | 27.7 | 97.9 | Kaulback | Pre-1940 | 1938 | Forest 600-1800m |
| 1017 | *Heterophasia pulchella* | label | 27.7 | 97.9 | Kaulback | Pre-1940 | 1938 | Forest 600-1800m |
| 1021 | *Heterophasia pulchella* | label | 27.7 | 97.9 | Kaulback | Pre-1940 | 1938 | Forest 600-1800m |
| 1026 | *Heterophasia pulchella* | label | 27.72 | 97.92 | Kaulback | Pre-1940 | 1938 | Forest > 1800m |
| 1054 | *Heterophasia pulchella* | label | 27.7 | 97.9 | Kaulback | Pre-1940 | 1938 | Forest 600-1800m |
| 3180 | *Hirundo rustica* | label | 27.7 | 97.9 | Kaulback | Pre-1940 | 1938 | Forest 600-1800m |
| 4985 | *Hirundo rustica* | label | 27.7 | 97.9 | Kaulback | Pre-1940 | 1938 | Forest 600-1800m |
| 2955 | *Hypsipetes mcclelandi* | label | 27.7 | 97.9 | Kaulback | Pre-1940 | 1938 | Forest 600-1800m |
| 4877 | *Jynx torquilla* | label | 27.7 | 97.9 | Kaulback | Pre-1940 | 1938 | Forest 600-1800m |
| 2799 | *Leiothrix argentauris* | label | 27.7 | 97.9 | Kaulback | Pre-1940 | 1938 | Forest 600-1800m |
| 2803 | *Leiothrix argentauris* | label | 27.7 | 97.9 | Kaulback | Pre-1940 | 1938 | Forest 600-1800m |
| 5091 | *Macropygia unchall* | label | 27.7 | 97.9 | Kaulback | Pre-1940 | 1938 | Forest 600-1800m |
| 4810 | *Megalaima franklinii* | label | 27.7 | 97.9 | Kaulback | Pre-1940 | 1938 | Forest 600-1800m |
| 4811 | *Megalaima franklinii* | label | 27.7 | 97.9 | Kaulback | Pre-1940 | 1938 | Forest 600-1800m |
| 4812 | *Megalaima franklinii* | label | 27.7 | 97.9 | Kaulback | Pre-1940 | 1938 | Forest 600-1800m |
| 4813 | *Megalaima franklinii* | label | 27.7 | 97.9 | Kaulback | Pre-1940 | 1938 | Forest 600-1800m |
| 4808 | *Megalaima virens* | label | 27.7 | 97.9 | Kaulback | Pre-1940 | 1938 | Forest 600-1800m |
| 3678 | *Melanochlora sultanea* | label | 27.7 | 97.9 | Kaulback | Pre-1940 | 1938 | Forest 600-1800m |
| 4977 | *Motacilla alba* | label | 27.7 | 97.9 | Kaulback | Pre-1940 | 1938 | Forest 600-1800m |
| 4980 | *Motacilla alba* | label | 27.7 | 97.9 | Kaulback | Pre-1940 | 1938 | Forest 600-1800m |
| 4981 | *Motacilla alba* | label | 27.7 | 97.9 | Kaulback | Pre-1940 | 1938 | Forest 600-1800m |
| 4962 | *Motacilla citriola* | label | 27.7 | 97.9 | Kaulback | Pre-1940 | 1938 | Forest 600-1800m |
| 3519 | *Niltava grandis* | label | 27.7 | 97.9 | Kaulback | Pre-1940 | 1938 | Forest 600-1800m |
| 3671 | *Parus spilnotus* | label | 27.7 | 97.9 | Kaulback | Pre-1940 | 1938 | Forest 600-1800m |
| 3411 | *Phylloscopus magnirostris* | label | 27.7 | 97.9 | Kaulback | Pre-1940 | 1938 | Forest 600-1800m |
| 1746 | *Pomatorhinus ferruginosus* | label | 27.7 | 97.9 | Kaulback | Pre-1940 | 1938 | Forest 600-1800m |
| 1749 | *Pomatorhinus ferruginosus* | label | 27.7 | 97.9 | Kaulback | Pre-1940 | 1938 | Forest 600-1800m |
| 4967 | *Psarisomus dalhousiae* | label | 27.7 | 97.9 | Kaulback | Pre-1940 | 1938 | Forest 600-1800m |
| 2903 | *Pycnonotus leucocephalus* | label | 27.7 | 97.9 | Kaulback | Pre-1940 | 1938 | Forest 600-1800m |
| 2906 | *Pycnonotus leucocephalus* | label | 27.7 | 97.9 | Kaulback | Pre-1940 | 1938 | Forest 600-1800m |
| 3679 | *Sitta formosa* | label | 27.7 | 97.9 | Kaulback | Pre-1940 | 1938 | Forest 600-1800m |
| 3680 | *Sitta formosa* | label | 27.7 | 97.9 | Kaulback | Pre-1940 | 1938 | Forest 600-1800m |
| 1949 | *Sphenocichla roberti* | label | 27.2 | 97.9 | Kaulback | Pre-1940 | 1938 | Forest 600-1800m |
| 1456 | *Stachyris nigriceps* | label | 27.7 | 97.9 | Kaulback | Pre-1940 | 1938 | Forest 600-1800m |
| 2021 | *Stachyris nigriceps* | label | 27.7 | 97.9 | Kaulback | Pre-1940 | 1938 | Forest 600-1800m |
| 3346 | *Yuhina flavicollis* | label | 27.7 | 97.9 | Kaulback | Pre-1940 | 1938 | Forest 600-1800m |
| 4949 | *Gecinulus grantia* | label | 27.5 | 97.83 | Kaulback | Pre-1940 | 1938 | Shrub/Bush/Fern |
| 5066 | *Lophura leucomelanus* | label | 27.5 | 97.83 | Kaulback | Pre-1940 | 1938 | Shrub/Bush/Fern |
| 4646 | *Dicrurus paradiseus* | label | 27.42 | 97.78 | Kaulback | Pre-1940 | 1938 | Forest 600-1800m |
| 4890 | *Micropternus brachyurus* | label | 27.42 | 97.78 | Kaulback | Pre-1940 | 1938 | Forest 600-1800m |
| 5001 | *Pericrocotus flammeus* | label | 27.42 | 97.78 | Kaulback | Pre-1940 | 1938 | Forest 600-1800m |
| 5079 | *Polyplectron bicalcaratum* | label | 27.42 | 97.78 | Kaulback | Pre-1940 | 1938 | Forest 600-1800m |
| 3224 | *Actinodura waldeni* | label | 28.13 | 97.75 | Kaulback | Pre-1940 | 1938 | Forest > 1800m |
| 3205 | *Actinodura egertoni* | label | 27.6 | 97.87 | Kaulback | Pre-1940 | 1939 | Forest 600-1800m |
| 4963 | *Hirundo striolata* | label | 27.6 | 97.87 | Kaulback | Pre-1940 | 1939 | Forest 600-1800m |
| 4875 | *Jynx torquilla* | label | 27.7 | 97.9 | Kaulback | Pre-1940 | 1939 | Forest 600-1800m |
| 4876 | *Jynx torquilla* | label | 27.7 | 97.9 | Kaulback | Pre-1940 | 1939 | Forest 600-1800m |
| 2001-5 | *Alcippe morrisonia* | GPS | 27.37 | 97.89 | Renner | Post-2000 | 2001 | Rock/boulder |
| 2001-3 | *Cinclidium leucurum* | GPS | 27.37 | 97.89 | Renner | Post-2000 | 2001 | Rock/boulder |
| 2001-4 | *Niltava sundara* | GPS | 27.37 | 97.89 | Renner | Post-2000 | 2001 | Rock/boulder |
| 2001-120 | *Zoothera dixoni* | GPS | 27.95 | 97.66 | Renner | Post-2000 | 2001 | Forest 600-1800m |
| 2001-121 | *Zoothera dixoni* | GPS | 27.95 | 97.66 | Renner | Post-2000 | 2001 | Forest 600-1800m |
| 2001-150 | *Accipiter virgatus* | GPS | 27.61 | 97.9 | Renner | Post-2000 | 2001 | Secondary forest 600-1800m |
| 2001-38 | *Alcippe rufogularis* | GPS | 27.61 | 97.9 | Renner | Post-2000 | 2001 | Secondary forest 600-1800m |
| 2001-147 | *Cissa hypoleuca* | GPS | 27.61 | 97.9 | Renner | Post-2000 | 2001 | Secondary forest 600-1800m |
| 2001-80 | *Cissa hypoleuca* | GPS | 27.61 | 97.9 | Renner | Post-2000 | 2001 | Secondary forest 600-1800m |
| 2001-41 | *Culicicapa ceylonensis* | GPS | 27.61 | 97.9 | Renner | Post-2000 | 2001 | Secondary forest 600-1800m |
| 2001-148 | *Garrulax nuchalis* | GPS | 27.61 | 97.9 | Renner | Post-2000 | 2001 | Secondary forest 600-1800m |
| 2001-149 | *Garrulax nuchalis* | GPS | 27.61 | 97.9 | Renner | Post-2000 | 2001 | Secondary forest 600-1800m |
| 2001-118 | *Lophura leucomelanus* | GPS | 27.61 | 97.9 | Renner | Post-2000 | 2001 | Secondary forest 600-1800m |
| 2001-39 | *Niltava grandis* | GPS | 27.61 | 97.9 | Renner | Post-2000 | 2001 | Secondary forest 600-1800m |
| 2001-151 | *Niltava sundara* | GPS | 27.61 | 97.9 | Renner | Post-2000 | 2001 | Secondary forest 600-1800m |
| 2001-40 | *Niltava sundara* | GPS | 27.61 | 97.9 | Renner | Post-2000 | 2001 | Secondary forest 600-1800m |
| 2001-77 | *Niltava sundara* | GPS | 27.61 | 97.9 | Renner | Post-2000 | 2001 | Secondary forest 600-1800m |
| 2001-37 | *Pericrocotus roseus* | GPS | 27.61 | 97.9 | Renner | Post-2000 | 2001 | Secondary forest 600-1800m |
| 2001-152 | *Picus canus* | GPS | 27.61 | 97.9 | Renner | Post-2000 | 2001 | Secondary forest 600-1800m |
| 2001-81 | *Pnoepyga pusilla* | GPS | 27.61 | 97.9 | Renner | Post-2000 | 2001 | Secondary forest 600-1800m |
| 2001-78 | *Seicercus poliogenys* | GPS | 27.61 | 97.9 | Renner | Post-2000 | 2001 | Secondary forest 600-1800m |
| 2001-79 | *Seicercus poliogenys* | GPS | 27.61 | 97.9 | Renner | Post-2000 | 2001 | Secondary forest 600-1800m |
| 2001-146 | *Tarsiger cyanurus* | GPS | 27.61 | 97.9 | Renner | Post-2000 | 2001 | Secondary forest 600-1800m |
| 2001-59 | *Alcippe cinerea* | GPS | 27.75 | 97.82 | Renner | Post-2000 | 2001 | Forest 600-1800m |
| 2001-60 | *Alcippe cinerea* | GPS | 27.75 | 97.82 | Renner | Post-2000 | 2001 | Forest 600-1800m |
| 2001-61 | *Alcippe cinerea* | GPS | 27.75 | 97.82 | Renner | Post-2000 | 2001 | Forest 600-1800m |
| 2001-130 | *Alcippe morrisonia* | GPS | 27.75 | 97.82 | Renner | Post-2000 | 2001 | Forest 600-1800m |
| 2001-131 | *Alcippe morrisonia* | GPS | 27.75 | 97.82 | Renner | Post-2000 | 2001 | Forest 600-1800m |
| 2001-129 | *Brachypteryx leucophris* | GPS | 27.75 | 97.82 | Renner | Post-2000 | 2001 | Forest 600-1800m |
| 2001-58 | *Enicurus scouleri* | GPS | 27.75 | 97.82 | Renner | Post-2000 | 2001 | Forest 600-1800m |
| 2001-135 | *Glaucidium cuculoides* | GPS | 27.75 | 97.82 | Renner | Post-2000 | 2001 | Forest 600-1800m |
| 2001-133 | *Liocichla phoenicea* | GPS | 27.75 | 97.82 | Renner | Post-2000 | 2001 | Forest 600-1800m |
| 2001-132 | *Niltava grandis* | GPS | 27.75 | 97.82 | Renner | Post-2000 | 2001 | Forest 600-1800m |
| 2001-64 | *Sasia ochracea* | GPS | 27.75 | 97.82 | Renner | Post-2000 | 2001 | Forest 600-1800m |
| 2001-134 | *Tarsiger cyanurus* | GPS | 27.75 | 97.82 | Renner | Post-2000 | 2001 | Forest 600-1800m |
| 2001-160 | *Aethopyga saturata* | GPS | 27.47 | 97.72 | Renner | Post-2000 | 2001 | Forest 600-1800m |
| 2001-172 | *Dicrurus aeneus* | GPS | 27.47 | 97.72 | Renner | Post-2000 | 2001 | Forest 600-1800m |
| 2001-175 | *Niltava sundara* | GPS | 27.47 | 97.72 | Renner | Post-2000 | 2001 | Forest 600-1800m |
| 2001-161 | *Pseudominla castaneceps* | GPS | 27.47 | 97.72 | Renner | Post-2000 | 2001 | Forest 600-1800m |
| 2001-169 | *Sitta formosa* | GPS | 27.47 | 97.72 | Renner | Post-2000 | 2001 | Forest 600-1800m |
| 2001-170 | *Sitta formosa* | GPS | 27.47 | 97.72 | Renner | Post-2000 | 2001 | Forest 600-1800m |
| 2001-173 | *Sphenocichla humei* | GPS | 27.47 | 97.72 | Renner | Post-2000 | 2001 | Forest 600-1800m |
| 2001-174 | *Sphenocichla humei* | GPS | 27.47 | 97.72 | Renner | Post-2000 | 2001 | Forest 600-1800m |
| 2001-171 | *Yuhina bakeri* | GPS | 27.47 | 97.72 | Renner | Post-2000 | 2001 | Forest 600-1800m |
| 2001-123 | *Aethopyga saturata* | GPS | 27.83 | 97.76 | Renner | Post-2000 | 2001 | Forest 600-1800m |
| 2001-127 | *Aethopyga saturata* | GPS | 27.83 | 97.76 | Renner | Post-2000 | 2001 | Forest 600-1800m |
| 2001-66 | *Ficedula monileger* | GPS | 27.83 | 97.76 | Renner | Post-2000 | 2001 | Forest 600-1800m |
| 2001-128 | *Ficedula strophiata* | GPS | 27.83 | 97.76 | Renner | Post-2000 | 2001 | Forest 600-1800m |
| 2001-62 | *Garrulax striatus* | GPS | 27.83 | 97.76 | Renner | Post-2000 | 2001 | Forest 600-1800m |
| 2001-68 | *Niltava grandis* | GPS | 27.83 | 97.76 | Renner | Post-2000 | 2001 | Forest 600-1800m |
| 2001-70 | *Niltava sundara* | GPS | 27.83 | 97.76 | Renner | Post-2000 | 2001 | Forest 600-1800m |
| 2001-125 | *Parus monticolus* | GPS | 27.83 | 97.76 | Renner | Post-2000 | 2001 | Forest 600-1800m |
| 2001-124 | *Phoenicurus auroreus* | GPS | 27.83 | 97.76 | Renner | Post-2000 | 2001 | Forest 600-1800m |
| 2001-126 | *Rhipidura albicollis* | GPS | 27.83 | 97.76 | Renner | Post-2000 | 2001 | Forest 600-1800m |
| 2001-63 | *Rhipidura albicollis* | GPS | 27.83 | 97.76 | Renner | Post-2000 | 2001 | Forest 600-1800m |
| 2001-69 | *Rhipidura albicollis* | GPS | 27.83 | 97.76 | Renner | Post-2000 | 2001 | Forest 600-1800m |
| 2001-71 | *Seicercus poliogenys* | GPS | 27.83 | 97.76 | Renner | Post-2000 | 2001 | Forest 600-1800m |
| 2001-177 | *Otus sunia* | GPS | 27.41 | 97.67 | Renner | Post-2000 | 2001 | Forest 600-1800m |
| 2001-178 | *Otus sunia* | GPS | 27.41 | 97.67 | Renner | Post-2000 | 2001 | Forest 600-1800m |
| 2001-176 | *Pellorneum ruficeps* | GPS | 27.41 | 97.67 | Renner | Post-2000 | 2001 | Forest 600-1800m |
| 2001-34 | *Abroscopus albogularis* | GPS | 27.49 | 97.82 | Renner | Post-2000 | 2001 | Forest 600-1800m |
| 2001-35 | *Abroscopus albogularis* | GPS | 27.49 | 97.82 | Renner | Post-2000 | 2001 | Forest 600-1800m |
| 2001-159 | *Aethopyga siparaja* | GPS | 27.49 | 97.82 | Renner | Post-2000 | 2001 | Forest 600-1800m |
| 2001-36 | *Alcippe morrisonia* | GPS | 27.49 | 97.82 | Renner | Post-2000 | 2001 | Forest 600-1800m |
| 2001-166 | *Alcippe rufogularis* | GPS | 27.49 | 97.82 | Renner | Post-2000 | 2001 | Forest 600-1800m |
| 2001-167 | *Alcippe rufogularis* | GPS | 27.49 | 97.82 | Renner | Post-2000 | 2001 | Forest 600-1800m |
| 2001-26 | *Alcippe rufogularis* | GPS | 27.49 | 97.82 | Renner | Post-2000 | 2001 | Forest 600-1800m |
| 2001-27 | *Alcippe rufogularis* | GPS | 27.49 | 97.82 | Renner | Post-2000 | 2001 | Forest 600-1800m |
| 2001-168 | *Alophoixus flaveolus* | GPS | 27.49 | 97.82 | Renner | Post-2000 | 2001 | Forest 600-1800m |
| 2001-20 | *Alophoixus flaveolus* | GPS | 27.49 | 97.82 | Renner | Post-2000 | 2001 | Forest 600-1800m |
| 2001-33 | *Alophoixus flaveolus* | GPS | 27.49 | 97.82 | Renner | Post-2000 | 2001 | Forest 600-1800m |
| 2001-158 | *Arachnothera magna* | GPS | 27.49 | 97.82 | Renner | Post-2000 | 2001 | Forest <600m |
| 2001-19 | *Arachnothera magna* | GPS | 27.49 | 97.82 | Renner | Post-2000 | 2001 | Forest 600-1800m |
| 2001-13 | *Dendrocitta frontalis* | GPS | 27.49 | 97.82 | Renner | Post-2000 | 2001 | Forest 600-1800m |
| 2001-14 | *Dendrocitta frontalis* | GPS | 27.49 | 97.82 | Renner | Post-2000 | 2001 | Forest 600-1800m |
| 2001-12 | *Ficedula monileger* | GPS | 27.49 | 97.82 | Renner | Post-2000 | 2001 | Forest 600-1800m |
| 2001-7 | *Ficedula monileger* | GPS | 27.49 | 97.82 | Renner | Post-2000 | 2001 | Forest 600-1800m |
| 2001-8 | *Ficedula monileger* | GPS | 27.49 | 97.82 | Renner | Post-2000 | 2001 | Forest 600-1800m |
| 2001-164 | *Ficedula tricolor* | GPS | 27.49 | 97.82 | Renner | Post-2000 | 2001 | Forest <600m |
| 2001-15 | *Heterophasia picaoides* | GPS | 27.49 | 97.82 | Renner | Post-2000 | 2001 | Forest 600-1800m |
| 2001-16 | *Heterophasia picaoides* | GPS | 27.49 | 97.82 | Renner | Post-2000 | 2001 | Forest 600-1800m |
| 2001-18 | *Leiothrix argentauris* | GPS | 27.49 | 97.82 | Renner | Post-2000 | 2001 | Forest 600-1800m |
| 2001-163 | *Minla ignotincta* | GPS | 27.49 | 97.82 | Renner | Post-2000 | 2001 | Forest 600-1800m |
| 2001-21 | *Napothera brevicaudata* | GPS | 27.49 | 97.82 | Renner | Post-2000 | 2001 | Forest 600-1800m |
| 2001-162 | *Napothera epilepidota* | GPS | 27.49 | 97.82 | Renner | Post-2000 | 2001 | Forest 600-1800m |
| 2001-165 | *Niltava macgrigoriae* | GPS | 27.49 | 97.82 | Renner | Post-2000 | 2001 | Forest 600-1800m |
| 2001-9 | *Paradoxornis atrosupercilliaris* | GPS | 27.49 | 97.82 | Renner | Post-2000 | 2001 | Forest 600-1800m |
| 2001-11 | *Pomatorhinus ferruginosus* | GPS | 27.49 | 97.82 | Renner | Post-2000 | 2001 | Forest 600-1800m |
| 2001-17 | *Pomatorhinus ferruginosus* | GPS | 27.49 | 97.82 | Renner | Post-2000 | 2001 | Forest 600-1800m |
| 2001-22 | *Sasia ochracea* | GPS | 27.49 | 97.82 | Renner | Post-2000 | 2001 | Forest 600-1800m |
| 2001-23 | *Sasia ochracea* | GPS | 27.49 | 97.82 | Renner | Post-2000 | 2001 | Forest 600-1800m |
| 2001-29 | *Seicercus poliogenys* | GPS | 27.49 | 97.82 | Renner | Post-2000 | 2001 | Forest 600-1800m |
| 2001-30 | *Seicercus poliogenys* | GPS | 27.49 | 97.82 | Renner | Post-2000 | 2001 | Forest 600-1800m |
| 2001-10 | *Serilophus lunatus* | GPS | 27.49 | 97.82 | Renner | Post-2000 | 2001 | Forest 600-1800m |
| 2001-6 | *Stachyris chrysaea* | GPS | 27.49 | 97.82 | Renner | Post-2000 | 2001 | Forest 600-1800m |
| 2001-24 | *Stachyris nigriceps* | GPS | 27.49 | 97.82 | Renner | Post-2000 | 2001 | Forest 600-1800m |
| 2001-25 | *Stachyris nigriceps* | GPS | 27.49 | 97.82 | Renner | Post-2000 | 2001 | Forest 600-1800m |
| 2001-28 | *Stachyris nigriceps* | GPS | 27.49 | 97.82 | Renner | Post-2000 | 2001 | Forest 600-1800m |
| 2001-31 | *Stachyris striolata* | GPS | 27.49 | 97.82 | Renner | Post-2000 | 2001 | Forest 600-1800m |
| 2001-32 | *Stachyris striolata* | GPS | 27.49 | 97.82 | Renner | Post-2000 | 2001 | Forest 600-1800m |
| 2001-54 | *Alcippe cinerea* | GPS | 27.73 | 97.87 | Renner | Post-2000 | 2001 | Settlement |
| 2001-55 | *Alcippe cinerea* | GPS | 27.73 | 97.87 | Renner | Post-2000 | 2001 | Settlement |
| 2001-49 | *Alcippe morrisonia* | GPS | 27.73 | 97.87 | Renner | Post-2000 | 2001 | Settlement |
| 2001-52 | *Alcippe morrisonia* | GPS | 27.73 | 97.87 | Renner | Post-2000 | 2001 | Settlement |
| 2001-138 | *Brachypteryx leucophris* | GPS | 27.73 | 97.87 | Renner | Post-2000 | 2001 | Settlement |
| 2001-140 | *Cinclus pallasii* | GPS | 27.73 | 97.87 | Renner | Post-2000 | 2001 | Settlement |
| 2001-136 | *Enicurus schistaceus* | GPS | 27.73 | 97.87 | Renner | Post-2000 | 2001 | Settlement |
| 2001-141 | *Ficedula monileger* | GPS | 27.73 | 97.87 | Renner | Post-2000 | 2001 | Settlement |
| 2001-48 | *Ficedula monileger* | GPS | 27.73 | 97.87 | Renner | Post-2000 | 2001 | Settlement |
| 2001-53 | *Ficedula strophiata* | GPS | 27.73 | 97.87 | Renner | Post-2000 | 2001 | Settlement |
| 2001-57 | *Niltava sp.* | GPS | 27.73 | 97.87 | Renner | Post-2000 | 2001 | Settlement |
| 2001-137 | *Orthotomus cucullatus* | GPS | 27.73 | 97.87 | Renner | Post-2000 | 2001 | Settlement |
| 2001-42 | *Paradoxornis nipalensis* | GPS | 27.73 | 97.87 | Renner | Post-2000 | 2001 | Settlement |
| 2001-43 | *Paradoxornis nipalensis* | GPS | 27.73 | 97.87 | Renner | Post-2000 | 2001 | Settlement |
| 2001-139 | *Rhyacornis fuliginosus* | GPS | 27.73 | 97.87 | Renner | Post-2000 | 2001 | Settlement |
| 2001-50 | *Stachyris nigriceps* | GPS | 27.73 | 97.87 | Renner | Post-2000 | 2001 | Settlement |
| 2001-51 | *Stachyris nigriceps* | GPS | 27.73 | 97.87 | Renner | Post-2000 | 2001 | Settlement |
| 2001-56 | *Stachyris ruficeps* | GPS | 27.73 | 97.87 | Renner | Post-2000 | 2001 | Settlement |
| 2001-1 | *Cyornis hainanus* | GPS | 27.349 | 97.4 | Renner | Post-2000 | 2001 | Settlement |
| 2001-2 | *Orthotomus sutorius* | GPS | 27.349 | 97.4 | Renner | Post-2000 | 2001 | Settlement |
| 2001-143 | *Alcippe morrisonia* | GPS | 27.68 | 97.90 | Renner | Post-2000 | 2001 | Forest 600-1800m |
| 2001-144 | *Alcippe morrisonia* | GPS | 27.68 | 97.90 | Renner | Post-2000 | 2001 | Forest 600-1800m |
| 2001-145 | *Alcippe morrisonia* | GPS | 27.68 | 97.90 | Renner | Post-2000 | 2001 | Forest 600-1800m |
| 2001-142 | *Blythipicus pyrrhotis* | GPS | 27.68 | 97.90 | Renner | Post-2000 | 2001 | Forest 600-1800m |
| 2001-44 | *Niltava grandis* | GPS | 27.68 | 97.90 | Renner | Post-2000 | 2001 | Forest 600-1800m |
| 2001-45 | *Rimator malacoptilus* | GPS | 27.68 | 97.90 | Renner | Post-2000 | 2001 | Forest 600-1800m |
| 2001-46 | *Rimator malacoptilus* | GPS | 27.68 | 97.90 | Renner | Post-2000 | 2001 | Forest 600-1800m |
| 2001-47 | *Yuhina nigrimenta* | GPS | 27.68 | 97.90 | Renner | Post-2000 | 2001 | Forest 600-1800m |
| 2001-93 | *Aethopyga nipalensis* | GPS | 28.17 | 97.68 | Renner | Post-2000 | 2001 | Settlement |
| 2001-89 | *Alcippe cinereiceps* | GPS | 28.17 | 97.68 | Renner | Post-2000 | 2001 | Settlement |
| 2001-100 | *Alcippe cinereiceps* | GPS | 28.17 | 97.68 | Renner | Post-2000 | 2001 | Settlement |
| 2001-98 | *Alcippe cinereiceps* | GPS | 28.17 | 97.68 | Renner | Post-2000 | 2001 | Settlement |
| 2001-99 | *Alcippe vinipectus* | GPS | 28.17 | 97.68 | Renner | Post-2000 | 2001 | Settlement |
| 2001-97 | *Garrulax affinis* | GPS | 28.17 | 97.68 | Renner | Post-2000 | 2001 | Settlement |
| 2001-96 | *Garrulax striatus* | GPS | 28.17 | 97.68 | Renner | Post-2000 | 2001 | Settlement |
| 2001-101 | *Garrulax subunicolor* | GPS | 28.17 | 97.68 | Renner | Post-2000 | 2001 | Settlement |
| 2001-91 | *Heterophasia picaoides* | GPS | 28.17 | 97.68 | Renner | Post-2000 | 2001 | Settlement |
| 2001-92 | *Heterophasia picaoides* | GPS | 28.17 | 97.68 | Renner | Post-2000 | 2001 | Settlement |
| 2001-103 | *Lophophorus impejanus* | GPS | 28.17 | 97.68 | Renner | Post-2000 | 2001 | Settlement |
| 2001-104 | *Lophophorus sclateri* | GPS | 28.17 | 97.68 | Renner | Post-2000 | 2001 | Settlement |
| 2001-94 | *Parus monticolus* | GPS | 28.17 | 97.68 | Renner | Post-2000 | 2001 | Settlement |
| 2001-95 | *Parus monticolus* | GPS | 28.17 | 97.68 | Renner | Post-2000 | 2001 | Settlement |
| 2001-102 | *Prunella immaculata* | GPS | 28.17 | 97.68 | Renner | Post-2000 | 2001 | Settlement |
| 2001-88 | *Pseudominla castaneceps* | GPS | 28.17 | 97.68 | Renner | Post-2000 | 2001 | Settlement |
| 2001-85 | *Stachyris ruficeps* | GPS | 28.17 | 97.68 | Renner | Post-2000 | 2001 | Settlement |
| 2001-86 | *Stachyris ruficeps* | GPS | 28.17 | 97.68 | Renner | Post-2000 | 2001 | Settlement |
| 2001-87 | *Stachyris ruficeps* | GPS | 28.17 | 97.68 | Renner | Post-2000 | 2001 | Settlement |
| 2001-90 | *Urocissa flavirostris* | GPS | 28.17 | 97.68 | Renner | Post-2000 | 2001 | Settlement |
| 2001-82 | *Ficedula monileger* | GPS | 28.04 | 97.57 | Renner | Post-2000 | 2001 | Settlement |
| 2001-111 | *Orthotomus cucullatus* | GPS | 28.04 | 97.57 | Renner | Post-2000 | 2001 | Settlement |
| 2001-112 | *Sasia ochracea* | GPS | 28.04 | 97.57 | Renner | Post-2000 | 2001 | Settlement |
| 2001-83 | *Sasia ochracea* | GPS | 28.04 | 97.57 | Renner | Post-2000 | 2001 | Settlement |
| 2001-110 | *Stachyris chrysaea* | GPS | 28.04 | 97.57 | Renner | Post-2000 | 2001 | Settlement |
| 2001-156 | *Aethopyga saturata* | GPS | 28.05 | 97.65 | Renner | Post-2000 | 2001 | Settlement |
| 2001-105 | *Alcippe cinerea* | GPS | 28.05 | 97.65 | Renner | Post-2000 | 2001 | Settlement |
| 2001-106 | *Alcippe cinerea* | GPS | 28.05 | 97.65 | Renner | Post-2000 | 2001 | Settlement |
| 2001-109 | *Alcippe cinerea* | GPS | 28.05 | 97.65 | Renner | Post-2000 | 2001 | Settlement |
| 2001-157 | *Ficedula strophiata* | GPS | 28.05 | 97.65 | Renner | Post-2000 | 2001 | Settlement |
| 2001-113 | *Garrulax erythrocephalus* | GPS | 28.05 | 97.65 | Renner | Post-2000 | 2001 | Settlement |
| 2001-114 | *Garrulax erythrocephalus* | GPS | 28.05 | 97.65 | Renner | Post-2000 | 2001 | Settlement |
| 2001-115 | *Garrulax erythrocephalus* | GPS | 28.05 | 97.65 | Renner | Post-2000 | 2001 | Settlement |
| 2001-116 | *Garrulax erythrocephalus* | GPS | 28.05 | 97.65 | Renner | Post-2000 | 2001 | Settlement |
| 2001-117 | *Heterophasia picaoides* | GPS | 28.05 | 97.65 | Renner | Post-2000 | 2001 | Settlement |
| 2001-154 | *Leiothrix argentauris* | GPS | 28.05 | 97.65 | Renner | Post-2000 | 2001 | Settlement |
| 2001-155 | *Leiothrix argentauris* | GPS | 28.05 | 97.65 | Renner | Post-2000 | 2001 | Settlement |
| 2001-119 | *Prunella immaculata* | GPS | 28.05 | 97.65 | Renner | Post-2000 | 2001 | Settlement |
| 2001-84 | *Seicercus poliogenys* | GPS | 28.05 | 97.65 | Renner | Post-2000 | 2001 | Settlement |
| 2001-153 | *Stachyris chrysaea* | GPS | 28.05 | 97.65 | Renner | Post-2000 | 2001 | Settlement |
| 2001-107 | *Stachyris nigriceps* | GPS | 28.05 | 97.65 | Renner | Post-2000 | 2001 | Settlement |
| 2001-108 | *Stachyris nigriceps* | GPS | 28.05 | 97.65 | Renner | Post-2000 | 2001 | Settlement |
| 2001-72 | *Ficedula monileger* | GPS | 27.91 | 97.7 | Renner | Post-2000 | 2001 | Forest 600-1800m |
| 2001-76 | *Ficedula monileger* | GPS | 27.91 | 97.7 | Renner | Post-2000 | 2001 | Forest 600-1800m |
| 2001-65 | *Ficedula strophiata* | GPS | 27.91 | 97.7 | Renner | Post-2000 | 2001 | Forest 600-1800m |
| 2001-67 | *Ficedula strophiata* | GPS | 27.91 | 97.7 | Renner | Post-2000 | 2001 | Forest 600-1800m |
| 2001-75 | *Ficedula strophiata* | GPS | 27.91 | 97.7 | Renner | Post-2000 | 2001 | Forest 600-1800m |
| 2001-122 | *Myophonus caeruleus* | GPS | 27.91 | 97.7 | Renner | Post-2000 | 2001 | Forest 600-1800m |
| 2001-73 | *Niltava grandis* | GPS | 27.91 | 97.7 | Renner | Post-2000 | 2001 | Forest 600-1800m |
| 2001-74 | *Niltava sundara* | GPS | 27.91 | 97.7 | Renner | Post-2000 | 2001 | Forest 600-1800m |
| 2004-129 | *Alcedo athis* | GPS | 27.48 | 97.82 | Renner | Post-2000 | 2004 | Forest 600-1800m |
| 2004-147 | *Alcedo hercules* | GPS | 27.48 | 97.82 | Renner | Post-2000 | 2004 | Forest 600-1800m |
| 2004-178 | *Abroscopus albogularis* | GPS | 27.43 | 97.67 | Renner | Post-2000 | 2004 | Forest 600-1800m |
| 2004-183 | *Aethopyga saturata* | GPS | 27.43 | 97.67 | Renner | Post-2000 | 2004 | Forest 600-1800m |
| 2004-193 | *Alcippe morrisonia* | GPS | 27.43 | 97.67 | Renner | Post-2000 | 2004 | Forest 600-1800m |
| 2004-203 | *Alcippe rufogularis* | GPS | 27.43 | 97.67 | Renner | Post-2000 | 2004 | Forest 600-1800m |
| 2004-204 | *Alcippe rufogularis* | GPS | 27.43 | 97.67 | Renner | Post-2000 | 2004 | Forest 600-1800m |
| 2004-205 | *Alcippe rufogularis* | GPS | 27.43 | 97.67 | Renner | Post-2000 | 2004 | Forest 600-1800m |
| 2004-173 | *Alophoixus flaveolus* | GPS | 27.43 | 97.67 | Renner | Post-2000 | 2004 | Forest 600-1800m |
| 2004-187 | *Alophoixus flaveolus* | GPS | 27.43 | 97.67 | Renner | Post-2000 | 2004 | Forest 600-1800m |
| 2004-194 | *Alophoixus flaveolus* | GPS | 27.43 | 97.67 | Renner | Post-2000 | 2004 | Forest 600-1800m |
| 2004-198 | *Arborophila rufogularis* | GPS | 27.43 | 97.67 | Renner | Post-2000 | 2004 | Forest 600-1800m |
| 2004-172 | *Chalcophaps indica* | GPS | 27.43 | 97.67 | Renner | Post-2000 | 2004 | Forest 600-1800m |
| 2004-176 | *Chalcophaps indica* | GPS | 27.43 | 97.67 | Renner | Post-2000 | 2004 | Forest 600-1800m |
| 2004-182 | *Cissa chinensis* | GPS | 27.43 | 97.67 | Renner | Post-2000 | 2004 | Forest 600-1800m |
| 2004-170 | *Enicurus maculatus* | GPS | 27.43 | 97.67 | Renner | Post-2000 | 2004 | Forest 600-1800m |
| 2004-177 | *Ficedula hyperythra* | GPS | 27.43 | 97.67 | Renner | Post-2000 | 2004 | Forest 600-1800m |
| 2004-196 | *Ficedula hyperythra* | GPS | 27.43 | 97.67 | Renner | Post-2000 | 2004 | Forest 600-1800m |
| 2004-163 | *Ficedula monileger* | GPS | 27.43 | 97.67 | Renner | Post-2000 | 2004 | Forest 600-1800m |
| 2004-171 | *Harpactes erythrocephalus* | GPS | 27.43 | 97.67 | Renner | Post-2000 | 2004 | Forest 600-1800m |
| 2004-174 | *Harpactes erythrocephalus* | GPS | 27.43 | 97.67 | Renner | Post-2000 | 2004 | Forest 600-1800m |
| 2004-179 | *Leiothrix argentauris* | GPS | 27.43 | 97.67 | Renner | Post-2000 | 2004 | Forest 600-1800m |
| 2004-180 | *Leiothrix argentauris* | GPS | 27.43 | 97.67 | Renner | Post-2000 | 2004 | Forest 600-1800m |
| 2004-195 | *Lophura leucomelanus* | GPS | 27.43 | 97.67 | Renner | Post-2000 | 2004 | Forest 600-1800m |
| 2004-184 | *Napothera brevicaudata* | GPS | 27.43 | 97.67 | Renner | Post-2000 | 2004 | Forest 600-1800m |
| 2004-207 | *Napothera brevicaudata* | GPS | 27.43 | 97.67 | Renner | Post-2000 | 2004 | Forest 600-1800m |
| 2004-202 | *Napothera epilepidota* | GPS | 27.43 | 97.67 | Renner | Post-2000 | 2004 | Forest 600-1800m |
| 2004-175 | *Niltava grandis* | GPS | 27.43 | 97.67 | Renner | Post-2000 | 2004 | Forest 600-1800m |
| 2004-206 | *Niltava grandis* | GPS | 27.43 | 97.67 | Renner | Post-2000 | 2004 | Forest 600-1800m |
| 2004-181 | *Niltava macgrigoriae* | GPS | 27.43 | 97.67 | Renner | Post-2000 | 2004 | Forest 600-1800m |
| 2004-169 | *Otus bakkamoena* | GPS | 27.43 | 97.67 | Renner | Post-2000 | 2004 | Forest 600-1800m |
| 2004-164 | *Paradoxornis ruficeps* | GPS | 27.43 | 97.67 | Renner | Post-2000 | 2004 | Forest 600-1800m |
| 2004-165 | *Paradoxornis ruficeps* | GPS | 27.43 | 97.67 | Renner | Post-2000 | 2004 | Forest 600-1800m |
| 2004-166 | *Paradoxornis ruficeps* | GPS | 27.43 | 97.67 | Renner | Post-2000 | 2004 | Forest 600-1800m |
| 2004-200 | *Pellorneum ruficeps* | GPS | 27.43 | 97.67 | Renner | Post-2000 | 2004 | Forest 600-1800m |
| 2004-201 | *Pellorneum ruficeps* | GPS | 27.43 | 97.67 | Renner | Post-2000 | 2004 | Forest 600-1800m |
| 2004-167 | *Pomatorhinus ferruginosus* | GPS | 27.43 | 97.67 | Renner | Post-2000 | 2004 | Forest 600-1800m |
| 2004-190 | *Pseudominla castaneceps* | GPS | 27.43 | 97.67 | Renner | Post-2000 | 2004 | Forest 600-1800m |
| 2004-191 | *Pseudominla castaneceps* | GPS | 27.43 | 97.67 | Renner | Post-2000 | 2004 | Forest 600-1800m |
| 2004-192 | *Pseudominla castaneceps* | GPS | 27.43 | 97.67 | Renner | Post-2000 | 2004 | Forest 600-1800m |
| 2004-199 | *Seicercus whistleri* | GPS | 27.43 | 97.67 | Renner | Post-2000 | 2004 | Forest 600-1800m |
| 2004-188 | *Yuhina nigrimenta* | GPS | 27.43 | 97.67 | Renner | Post-2000 | 2004 | Forest 600-1800m |
| 2004-189 | *Yuhina nigrimenta* | GPS | 27.43 | 97.67 | Renner | Post-2000 | 2004 | Forest 600-1800m |
| 2004-185 | *Yuhina zantholeuca* | GPS | 27.43 | 97.67 | Renner | Post-2000 | 2004 | Forest 600-1800m |
| 2004-186 | *Yuhina zantholeuca* | GPS | 27.43 | 97.67 | Renner | Post-2000 | 2004 | Forest 600-1800m |
| 2004-106 | *Abroscopus albogularis* | GPS | 27.49 | 97.82 | Renner | Post-2000 | 2004 | Forest 600-1800m |
| 2004-84 | *Abroscopus albogularis* | GPS | 27.49 | 97.82 | Renner | Post-2000 | 2004 | Forest 600-1800m |
| 2004-85 | *Abroscopus albogularis* | GPS | 27.49 | 97.82 | Renner | Post-2000 | 2004 | Forest 600-1800m |
| 2004-86 | *Abroscopus albogularis* | GPS | 27.49 | 97.82 | Renner | Post-2000 | 2004 | Forest 600-1800m |
| 2004-98 | *Abroscopus albogularis* | GPS | 27.49 | 97.82 | Renner | Post-2000 | 2004 | Forest 600-1800m |
| 2004-139 | *Aethopyga saturata* | GPS | 27.49 | 97.82 | Renner | Post-2000 | 2004 | Forest 600-1800m |
| 2004-154 | *Aethopyga saturata* | GPS | 27.49 | 97.82 | Renner | Post-2000 | 2004 | Forest 600-1800m |
| 2004-19 | *Aethopyga saturata* | GPS | 27.49 | 97.82 | Renner | Post-2000 | 2004 | Forest 600-1800m |
| 2004-87 | *Aethopyga siparaja* | GPS | 27.49 | 97.82 | Renner | Post-2000 | 2004 | Forest 600-1800m |
| 2004-030a | *Alcippe morrisonia* | GPS | 27.49 | 97.82 | Renner | Post-2000 | 2004 | Forest 600-1800m |
| 2004-030b | *Alcippe morrisonia* | GPS | 27.49 | 97.82 | Renner | Post-2000 | 2004 | Forest 600-1800m |
| 2004-110 | *Alcippe morrisonia* | GPS | 27.49 | 97.82 | Renner | Post-2000 | 2004 | Forest 600-1800m |
| 2004-115 | *Alcippe morrisonia* | GPS | 27.49 | 97.82 | Renner | Post-2000 | 2004 | Forest 600-1800m |
| 2004-116 | *Alcippe morrisonia* | GPS | 27.49 | 97.82 | Renner | Post-2000 | 2004 | Forest 600-1800m |
| 2004-126 | *Alcippe morrisonia* | GPS | 27.49 | 97.82 | Renner | Post-2000 | 2004 | Forest 600-1800m |
| 2004-127 | *Alcippe morrisonia* | GPS | 27.49 | 97.82 | Renner | Post-2000 | 2004 | Forest 600-1800m |
| 2004-135 | *Alcippe morrisonia* | GPS | 27.49 | 97.82 | Renner | Post-2000 | 2004 | Forest 600-1800m |
| 2004-138 | *Alcippe morrisonia* | GPS | 27.49 | 97.82 | Renner | Post-2000 | 2004 | Forest 600-1800m |
| 2004-140 | *Alcippe morrisonia* | GPS | 27.49 | 97.82 | Renner | Post-2000 | 2004 | Forest 600-1800m |
| 2004-23 | *Alcippe morrisonia* | GPS | 27.49 | 97.82 | Renner | Post-2000 | 2004 | Forest 600-1800m |
| 2004-24 | *Alcippe morrisonia* | GPS | 27.49 | 97.82 | Renner | Post-2000 | 2004 | Forest 600-1800m |
| 2004-25 | *Alcippe morrisonia* | GPS | 27.49 | 97.82 | Renner | Post-2000 | 2004 | Forest 600-1800m |
| 2004-27 | *Alcippe morrisonia* | GPS | 27.49 | 97.82 | Renner | Post-2000 | 2004 | Forest 600-1800m |
| 2004-28 | *Alcippe morrisonia* | GPS | 27.49 | 97.82 | Renner | Post-2000 | 2004 | Forest 600-1800m |
| 2004-29 | *Alcippe morrisonia* | GPS | 27.49 | 97.82 | Renner | Post-2000 | 2004 | Forest 600-1800m |
| 2004-46 | *Alcippe morrisonia* | GPS | 27.49 | 97.82 | Renner | Post-2000 | 2004 | Forest 600-1800m |
| 2004-47 | *Alcippe morrisonia* | GPS | 27.49 | 97.82 | Renner | Post-2000 | 2004 | Forest 600-1800m |
| 2004-67 | *Alcippe morrisonia* | GPS | 27.49 | 97.82 | Renner | Post-2000 | 2004 | Forest 600-1800m |
| 2004-68 | *Alcippe morrisonia* | GPS | 27.49 | 97.82 | Renner | Post-2000 | 2004 | Forest 600-1800m |
| 2004-80 | *Alcippe morrisonia* | GPS | 27.49 | 97.82 | Renner | Post-2000 | 2004 | Forest 600-1800m |
| 2004-81 | *Alcippe morrisonia* | GPS | 27.49 | 97.82 | Renner | Post-2000 | 2004 | Forest 600-1800m |
| 2004-82 | *Alcippe morrisonia* | GPS | 27.49 | 97.82 | Renner | Post-2000 | 2004 | Forest 600-1800m |
| 2004-83 | *Alcippe morrisonia* | GPS | 27.49 | 97.82 | Renner | Post-2000 | 2004 | Forest 600-1800m |
| 2004-1 | *Alcippe rufogularis* | GPS | 27.49 | 97.82 | Renner | Post-2000 | 2004 | Forest 600-1800m |
| 2004-10 | *Alcippe rufogularis* | GPS | 27.49 | 97.82 | Renner | Post-2000 | 2004 | Forest 600-1800m |
| 2004-100 | *Alcippe rufogularis* | GPS | 27.49 | 97.82 | Renner | Post-2000 | 2004 | Forest 600-1800m |
| 2004-11 | *Alcippe rufogularis* | GPS | 27.49 | 97.82 | Renner | Post-2000 | 2004 | Forest 600-1800m |
| 2004-2 | *Alcippe rufogularis* | GPS | 27.49 | 97.82 | Renner | Post-2000 | 2004 | Forest 600-1800m |
| 2004-3 | *Alcippe rufogularis* | GPS | 27.49 | 97.82 | Renner | Post-2000 | 2004 | Forest 600-1800m |
| 2004-4 | *Alcippe rufogularis* | GPS | 27.49 | 97.82 | Renner | Post-2000 | 2004 | Forest 600-1800m |
| 2004-5 | *Alcippe rufogularis* | GPS | 27.49 | 97.82 | Renner | Post-2000 | 2004 | Forest 600-1800m |
| 2004-55 | *Alcippe rufogularis* | GPS | 27.49 | 97.82 | Renner | Post-2000 | 2004 | Forest 600-1800m |
| 2004-56 | *Alcippe rufogularis* | GPS | 27.49 | 97.82 | Renner | Post-2000 | 2004 | Forest 600-1800m |
| 2004-65 | *Alcippe rufogularis* | GPS | 27.49 | 97.82 | Renner | Post-2000 | 2004 | Forest 600-1800m |
| 2004-9 | *Alcippe rufogularis* | GPS | 27.49 | 97.82 | Renner | Post-2000 | 2004 | Forest 600-1800m |
| 2004-112 | *Alophoixus flaveolus* | GPS | 27.49 | 97.82 | Renner | Post-2000 | 2004 | Forest 600-1800m |
| 2004-143 | *Alophoixus flaveolus* | GPS | 27.49 | 97.82 | Renner | Post-2000 | 2004 | Forest 600-1800m |
| 2004-144 | *Alophoixus flaveolus* | GPS | 27.49 | 97.82 | Renner | Post-2000 | 2004 | Forest 600-1800m |
| 2004-49 | *Alophoixus flaveolus* | GPS | 27.49 | 97.82 | Renner | Post-2000 | 2004 | Forest 600-1800m |
| 2004-50 | *Alophoixus flaveolus* | GPS | 27.49 | 97.82 | Renner | Post-2000 | 2004 | Forest 600-1800m |
| 2004-61 | *Alophoixus flaveolus* | GPS | 27.49 | 97.82 | Renner | Post-2000 | 2004 | Forest 600-1800m |
| 2004-96 | *Alophoixus flaveolus* | GPS | 27.49 | 97.82 | Renner | Post-2000 | 2004 | Forest 600-1800m |
| 2004-130 | *Arachnothera magna* | GPS | 27.49 | 97.82 | Renner | Post-2000 | 2004 | Forest 600-1800m |
| 2004-58 | *Arborophila atrogularis* | GPS | 27.49 | 97.82 | Renner | Post-2000 | 2004 | Forest 600-1800m |
| 2004-72 | *Brachypteryx hyperthyra* | GPS | 27.49 | 97.82 | Renner | Post-2000 | 2004 | Forest 600-1800m |
| 2004-73 | *Brachypteryx hyperthyra* | GPS | 27.49 | 97.82 | Renner | Post-2000 | 2004 | Forest 600-1800m |
| 2004-118 | *Brachypteryx sp.* | GPS | 27.49 | 97.82 | Renner | Post-2000 | 2004 | Forest 600-1800m |
| 2004-122 | *Culicicapa ceylonensis* | GPS | 27.49 | 97.82 | Renner | Post-2000 | 2004 | Forest 600-1800m |
| 2004-153 | *Culicicapa ceylonensis* | GPS | 27.49 | 97.82 | Renner | Post-2000 | 2004 | Forest 600-1800m |
| 2004-16 | *Culicicapa ceylonensis* | GPS | 27.49 | 97.82 | Renner | Post-2000 | 2004 | Forest 600-1800m |
| 2004-37 | *Culicicapa ceylonensis* | GPS | 27.49 | 97.82 | Renner | Post-2000 | 2004 | Forest 600-1800m |
| 2004-38 | *Culicicapa ceylonensis* | GPS | 27.49 | 97.82 | Renner | Post-2000 | 2004 | Forest 600-1800m |
| 2004-26 | *Dicrurus remifer* | GPS | 27.49 | 97.82 | Renner | Post-2000 | 2004 | Forest 600-1800m |
| 2004-123 | *Ficedula hyperythra* | GPS | 27.49 | 97.82 | Renner | Post-2000 | 2004 | Forest 600-1800m |
| 2004-136 | *Ficedula hyperythra* | GPS | 27.49 | 97.82 | Renner | Post-2000 | 2004 | Forest 600-1800m |
| 2004-101 | *Ficedula monileger* | GPS | 27.49 | 97.82 | Renner | Post-2000 | 2004 | Forest 600-1800m |
| 2004-12 | *Ficedula monileger* | GPS | 27.49 | 97.82 | Renner | Post-2000 | 2004 | Forest 600-1800m |
| 2004-18 | *Ficedula monileger* | GPS | 27.49 | 97.82 | Renner | Post-2000 | 2004 | Forest 600-1800m |
| 2004-6 | *Ficedula monileger* | GPS | 27.49 | 97.82 | Renner | Post-2000 | 2004 | Forest 600-1800m |
| 2004-22 | *Ficedula strophiata* | GPS | 27.49 | 97.82 | Renner | Post-2000 | 2004 | Forest 600-1800m |
| 2004-36 | *Ficedula strophiata* | GPS | 27.49 | 97.82 | Renner | Post-2000 | 2004 | Forest 600-1800m |
| 2004-7 | *Ficedula strophiata* | GPS | 27.49 | 97.82 | Renner | Post-2000 | 2004 | Forest 600-1800m |
| 2004-119 | *Garrulax leucolophus* | GPS | 27.49 | 97.82 | Renner | Post-2000 | 2004 | Forest 600-1800m |
| 2004-120 | *Garrulax leucolophus* | GPS | 27.49 | 97.82 | Renner | Post-2000 | 2004 | Forest 600-1800m |
| 2004-74 | *Garrulax leucolophus* | GPS | 27.49 | 97.82 | Renner | Post-2000 | 2004 | Forest 600-1800m |
| 2004-14 | *Glaucidium brodiei* | GPS | 27.49 | 97.82 | Renner | Post-2000 | 2004 | Forest 600-1800m |
| 2004-54 | *Glaucidium brodiei* | GPS | 27.49 | 97.82 | Renner | Post-2000 | 2004 | Forest 600-1800m |
| 2004-44 | *Jabouilleia naungmungensis* | GPS | 27.49 | 97.82 | Renner | Post-2000 | 2004 | Forest 600-1800m |
| 2004-45 | *Jabouilleia naungmungensis* | GPS | 27.49 | 97.82 | Renner | Post-2000 | 2004 | Forest 600-1800m |
| 2004-71 | *Jabouilleia naungmungensis* | GPS | 27.49 | 97.82 | Renner | Post-2000 | 2004 | Forest 600-1800m |
| 2004-105 | *Malacocincla abbotti* | GPS | 27.49 | 97.82 | Renner | Post-2000 | 2004 | Forest 600-1800m |
| 2004-145 | *Napothera brevicaudata* | GPS | 27.49 | 97.82 | Renner | Post-2000 | 2004 | Forest 600-1800m |
| 2004-146 | *Napothera brevicaudata* | GPS | 27.49 | 97.82 | Renner | Post-2000 | 2004 | Forest 600-1800m |
| 2004-8 | *Napothera brevicaudata* | GPS | 27.49 | 97.82 | Renner | Post-2000 | 2004 | Forest 600-1800m |
| 2004-159 | *Napothera epilepidota* | GPS | 27.49 | 97.82 | Renner | Post-2000 | 2004 | Forest 600-1800m |
| 2004-160 | *Napothera epilepidota* | GPS | 27.49 | 97.82 | Renner | Post-2000 | 2004 | Forest 600-1800m |
| 2004-35 | *Napothera epilepidota* | GPS | 27.49 | 97.82 | Renner | Post-2000 | 2004 | Forest 600-1800m |
| 2004-113 | *Niltava grandis* | GPS | 27.49 | 97.82 | Renner | Post-2000 | 2004 | Forest 600-1800m |
| 2004-114 | *Niltava grandis* | GPS | 27.49 | 97.82 | Renner | Post-2000 | 2004 | Forest 600-1800m |
| 2004-137 | *Niltava grandis* | GPS | 27.49 | 97.82 | Renner | Post-2000 | 2004 | Forest 600-1800m |
| 2004-142 | *Niltava grandis* | GPS | 27.49 | 97.82 | Renner | Post-2000 | 2004 | Forest 600-1800m |
| 2004-15 | *Niltava grandis* | GPS | 27.49 | 97.82 | Renner | Post-2000 | 2004 | Forest 600-1800m |
| 2004-152 | *Niltava grandis* | GPS | 27.49 | 97.82 | Renner | Post-2000 | 2004 | Forest 600-1800m |
| 2004-157 | *Niltava grandis* | GPS | 27.49 | 97.82 | Renner | Post-2000 | 2004 | Forest 600-1800m |
| 2004-20 | *Niltava grandis* | GPS | 27.49 | 97.82 | Renner | Post-2000 | 2004 | Forest 600-1800m |
| 2004-40 | *Niltava grandis* | GPS | 27.49 | 97.82 | Renner | Post-2000 | 2004 | Forest 600-1800m |
| 2004-59 | *Niltava grandis* | GPS | 27.49 | 97.82 | Renner | Post-2000 | 2004 | Forest 600-1800m |
| 2004-121 | *Niltava macgrigoriae* | GPS | 27.49 | 97.82 | Renner | Post-2000 | 2004 | Forest 600-1800m |
| 2004-132 | *Niltava macgrigoriae* | GPS | 27.49 | 97.82 | Renner | Post-2000 | 2004 | Forest 600-1800m |
| 2004-133 | *Niltava macgrigoriae* | GPS | 27.49 | 97.82 | Renner | Post-2000 | 2004 | Forest 600-1800m |
| 2004-62 | *Niltava macgrigoriae* | GPS | 27.49 | 97.82 | Renner | Post-2000 | 2004 | Forest 600-1800m |
| 2004-63 | *Niltava macgrigoriae* | GPS | 27.49 | 97.82 | Renner | Post-2000 | 2004 | Forest 600-1800m |
| 2004-97 | *Niltava macgrigoriae* | GPS | 27.49 | 97.82 | Renner | Post-2000 | 2004 | Forest 600-1800m |
| 2004-102 | *Niltava sundara* | GPS | 27.49 | 97.82 | Renner | Post-2000 | 2004 | Forest 600-1800m |
| 2004-104 | *Niltava sundara* | GPS | 27.49 | 97.82 | Renner | Post-2000 | 2004 | Forest 600-1800m |
| 2004-39 | *Niltava sundara* | GPS | 27.49 | 97.82 | Renner | Post-2000 | 2004 | Forest 600-1800m |
| 2004-78 | *Niltava sundara* | GPS | 27.49 | 97.82 | Renner | Post-2000 | 2004 | Forest 600-1800m |
| 2004-108 | *Passer montanus* | GPS | 27.49 | 97.82 | Renner | Post-2000 | 2004 | Forest 600-1800m |
| 2004-131 | *Pellorneum tickelli* | GPS | 27.49 | 97.82 | Renner | Post-2000 | 2004 | Forest 600-1800m |
| 2004-161 | *Pellorneum tickelli* | GPS | 27.49 | 97.82 | Renner | Post-2000 | 2004 | Forest 600-1800m |
| 2004-128 | *Pericrocotus ethologus* | GPS | 27.49 | 97.82 | Renner | Post-2000 | 2004 | Forest 600-1800m |
| 2004-134 | *Phylloscopus proregulus* | GPS | 27.49 | 97.82 | Renner | Post-2000 | 2004 | Forest 600-1800m |
| 2004-41 | *Pomatorhinus ferruginosus* | GPS | 27.49 | 97.82 | Renner | Post-2000 | 2004 | Forest 600-1800m |
| 2004-42 | *Pomatorhinus ferruginosus* | GPS | 27.49 | 97.82 | Renner | Post-2000 | 2004 | Forest 600-1800m |
| 2004-43 | *Pomatorhinus ferruginosus* | GPS | 27.49 | 97.82 | Renner | Post-2000 | 2004 | Forest 600-1800m |
| 2004-95 | *Pomatorhinus ferruginosus* | GPS | 27.49 | 97.82 | Renner | Post-2000 | 2004 | Forest 600-1800m |
| 2004-107 | *Pycnonotus jocosus* | GPS | 27.49 | 97.82 | Renner | Post-2000 | 2004 | Forest 600-1800m |
| 2004-117 | *Pycnonotus jocosus* | GPS | 27.49 | 97.82 | Renner | Post-2000 | 2004 | Forest 600-1800m |
| 2004-162 | *Pycnonotus jocosus* | GPS | 27.49 | 97.82 | Renner | Post-2000 | 2004 | Forest 600-1800m |
| 2004-48 | *Pycnonotus jocosus* | GPS | 27.49 | 97.82 | Renner | Post-2000 | 2004 | Forest 600-1800m |
| 2004-60 | *Pycnonotus jocosus* | GPS | 27.49 | 97.82 | Renner | Post-2000 | 2004 | Forest 600-1800m |
| 2004-13 | *Rhipidura albicollis* | GPS | 27.49 | 97.82 | Renner | Post-2000 | 2004 | Forest 600-1800m |
| 2004-141 | *Rhipidura albicollis* | GPS | 27.49 | 97.82 | Renner | Post-2000 | 2004 | Forest 600-1800m |
| 2004-52 | *Rhipidura albicollis* | GPS | 27.49 | 97.82 | Renner | Post-2000 | 2004 | Forest 600-1800m |
| 2004-17 | *Sasia ochracea* | GPS | 27.49 | 97.82 | Renner | Post-2000 | 2004 | Forest 600-1800m |
| 2004-88 | *Sasia ochracea* | GPS | 27.49 | 97.82 | Renner | Post-2000 | 2004 | Forest 600-1800m |
| 2004-99 | *Seicercus affinis* | GPS | 27.49 | 97.82 | Renner | Post-2000 | 2004 | Forest 600-1800m |
| 2004-155 | *Seicercus poliogenys* | GPS | 27.49 | 97.82 | Renner | Post-2000 | 2004 | Forest 600-1800m |
| 2004-156 | *Seicercus poliogenys* | GPS | 27.49 | 97.82 | Renner | Post-2000 | 2004 | Forest 600-1800m |
| 2004-51 | *Seicercus poliogenys* | GPS | 27.49 | 97.82 | Renner | Post-2000 | 2004 | Forest 600-1800m |
| 2004-66 | *Seicercus poliogenys* | GPS | 27.49 | 97.82 | Renner | Post-2000 | 2004 | Forest 600-1800m |
| 2004-111 | *Seicercus tephrocephalus* | GPS | 27.49 | 97.82 | Renner | Post-2000 | 2004 | Forest 600-1800m |
| 2004-57 | *Seicercus tephrocephalus* | GPS | 27.49 | 97.82 | Renner | Post-2000 | 2004 | Forest 600-1800m |
| 2004-94 | *Seicercus tephrocephalus* | GPS | 27.49 | 97.82 | Renner | Post-2000 | 2004 | Forest 600-1800m |
| 2004-64 | *Serilophus lunatus* | GPS | 27.49 | 97.82 | Renner | Post-2000 | 2004 | Forest 600-1800m |
| 2004-89 | *Serilophus lunatus* | GPS | 27.49 | 97.82 | Renner | Post-2000 | 2004 | Forest 600-1800m |
| 2004-90 | *Serilophus lunatus* | GPS | 27.49 | 97.82 | Renner | Post-2000 | 2004 | Forest 600-1800m |
| 2004-91 | *Serilophus lunatus* | GPS | 27.49 | 97.82 | Renner | Post-2000 | 2004 | Forest 600-1800m |
| 2004-92 | *Serilophus lunatus* | GPS | 27.49 | 97.82 | Renner | Post-2000 | 2004 | Forest 600-1800m |
| 2004-93 | *Serilophus lunatus* | GPS | 27.49 | 97.82 | Renner | Post-2000 | 2004 | Forest 600-1800m |
| 2004-158 | *Stachyris chrysaea* | GPS | 27.49 | 97.82 | Renner | Post-2000 | 2004 | Forest 600-1800m |
| 2004-103 | *Stachyris nigriceps* | GPS | 27.49 | 97.82 | Renner | Post-2000 | 2004 | Forest 600-1800m |
| 2004-124 | *Stachyris nigriceps* | GPS | 27.49 | 97.82 | Renner | Post-2000 | 2004 | Forest 600-1800m |
| 2004-125 | *Stachyris nigriceps* | GPS | 27.49 | 97.82 | Renner | Post-2000 | 2004 | Forest 600-1800m |
| 2004-21 | *Stachyris nigriceps* | GPS | 27.49 | 97.82 | Renner | Post-2000 | 2004 | Forest 600-1800m |
| 2004-31 | *Stachyris nigriceps* | GPS | 27.49 | 97.82 | Renner | Post-2000 | 2004 | Forest 600-1800m |
| 2004-32 | *Stachyris nigriceps* | GPS | 27.49 | 97.82 | Renner | Post-2000 | 2004 | Forest 600-1800m |
| 2004-33 | *Stachyris nigriceps* | GPS | 27.49 | 97.82 | Renner | Post-2000 | 2004 | Forest 600-1800m |
| 2004-34 | *Stachyris nigriceps* | GPS | 27.49 | 97.82 | Renner | Post-2000 | 2004 | Forest 600-1800m |
| 2004-109 | *Tesia olivea* | GPS | 27.49 | 97.82 | Renner | Post-2000 | 2004 | Forest 600-1800m |
| 2004-69 | *Tesia olivea* | GPS | 27.49 | 97.82 | Renner | Post-2000 | 2004 | Forest 600-1800m |
| 2004-70 | *Tesia olivea* | GPS | 27.49 | 97.82 | Renner | Post-2000 | 2004 | Forest 600-1800m |
| 2004-79 | *Tesia olivea* | GPS | 27.49 | 97.82 | Renner | Post-2000 | 2004 | Forest 600-1800m |
| 2004-53 | *Yuhina zantholeuca* | GPS | 27.49 | 97.82 | Renner | Post-2000 | 2004 | Forest 600-1800m |
| 2004-75 | *Yuhina zantholeuca* | GPS | 27.49 | 97.82 | Renner | Post-2000 | 2004 | Forest 600-1800m |
| 2004-76 | *Yuhina zantholeuca* | GPS | 27.49 | 97.82 | Renner | Post-2000 | 2004 | Forest 600-1800m |
| 2004-77 | *Yuhina zantholeuca* | GPS | 27.49 | 97.82 | Renner | Post-2000 | 2004 | Forest 600-1800m |
| 2005-47 | *Arachnothera magna* | GPS | 27.41 | 97.67 | Renner | Post-2000 | 2005 | Forest 600-1800m |
| 2005-49 | *Arachnothera magna* | GPS | 27.41 | 97.67 | Renner | Post-2000 | 2005 | Forest 600-1800m |
| 2005-46 | *Ficedula sapphira* | GPS | 27.41 | 97.67 | Renner | Post-2000 | 2005 | Forest 600-1800m |
| 2005-50 | *Hypsipetes mcclelandi* | GPS | 27.41 | 97.67 | Renner | Post-2000 | 2005 | Forest 600-1800m |
| 2005-52 | *Hypsipetes mcclelandi* | GPS | 27.41 | 97.67 | Renner | Post-2000 | 2005 | Forest 600-1800m |
| 2005-48 | *Megalaima virens* | GPS | 27.41 | 97.67 | Renner | Post-2000 | 2005 | Forest 600-1800m |
| 2005-51 | *Niltava grandis* | GPS | 27.41 | 97.67 | Renner | Post-2000 | 2005 | Forest 600-1800m |
| 2005-37 | *Abroscopus albogularis* | GPS | 27.49 | 97.82 | Renner | Post-2000 | 2005 | Forest 600-1800m |
| 2005-9 | *Aethopyga saturata* | GPS | 27.49 | 97.82 | Renner | Post-2000 | 2005 | Forest 600-1800m |
| 2005-19 | *Alcippe morrisonia* | GPS | 27.49 | 97.82 | Renner | Post-2000 | 2005 | Forest 600-1800m |
| 2005-21 | *Alcippe morrisonia* | GPS | 27.49 | 97.82 | Renner | Post-2000 | 2005 | Forest 600-1800m |
| 2005-23 | *Alcippe morrisonia* | GPS | 27.49 | 97.82 | Renner | Post-2000 | 2005 | Forest 600-1800m |
| 2005-39 | *Alcippe rufogularis* | GPS | 27.49 | 97.82 | Renner | Post-2000 | 2005 | Forest 600-1800m |
| 2005-2 | *Alophoixus flaveolus* | GPS | 27.49 | 97.82 | Renner | Post-2000 | 2005 | Forest 600-1800m |
| 2005-22 | *Alophoixus flaveolus* | GPS | 27.49 | 97.82 | Renner | Post-2000 | 2005 | Forest 600-1800m |
| 2005-3 | *Alophoixus flaveolus* | GPS | 27.49 | 97.82 | Renner | Post-2000 | 2005 | Forest 600-1800m |
| 2005-7 | *Alophoixus flaveolus* | GPS | 27.49 | 97.82 | Renner | Post-2000 | 2005 | Forest 600-1800m |
| 2005-25 | *Arachnothera magna* | GPS | 27.49 | 97.82 | Renner | Post-2000 | 2005 | Forest 600-1800m |
| 2005-45 | *Brachypteryx sp.* | GPS | 27.49 | 97.82 | Renner | Post-2000 | 2005 | Forest 600-1800m |
| 2005-6 | *Brachypteryx sp.* | GPS | 27.49 | 97.82 | Renner | Post-2000 | 2005 | Forest 600-1800m |
| 2005-17 | *Culicicapa ceylonensis* | GPS | 27.49 | 97.82 | Renner | Post-2000 | 2005 | Forest 600-1800m |
| 2005-13 | *Ficedula monileger* | GPS | 27.49 | 97.82 | Renner | Post-2000 | 2005 | Forest 600-1800m |
| 2005-31 | *Ficedula monileger* | GPS | 27.49 | 97.82 | Renner | Post-2000 | 2005 | Forest 600-1800m |
| 2005-34 | *Ficedula monileger* | GPS | 27.49 | 97.82 | Renner | Post-2000 | 2005 | Forest 600-1800m |
| 2005-42 | *Garrulax leucolophus* | GPS | 27.49 | 97.82 | Renner | Post-2000 | 2005 | Forest 600-1800m |
| 2005-27 | *Garrulax rufogularis* | GPS | 27.49 | 97.82 | Renner | Post-2000 | 2005 | Forest 600-1800m |
| 2005-10 | *Harpactes erythrocephalus* | GPS | 27.49 | 97.82 | Renner | Post-2000 | 2005 | Forest 600-1800m |
| 2005-35 | *Harpactes erythrocephalus* | GPS | 27.49 | 97.82 | Renner | Post-2000 | 2005 | Forest 600-1800m |
| 2005-36 | *Harpactes erythrocephalus* | GPS | 27.49 | 97.82 | Renner | Post-2000 | 2005 | Forest 600-1800m |
| 2005-18 | *Hemixos flavala* | GPS | 27.49 | 97.82 | Renner | Post-2000 | 2005 | Forest 600-1800m |
| 2005-30 | *Hemixos flavala* | GPS | 27.49 | 97.82 | Renner | Post-2000 | 2005 | Forest 600-1800m |
| 2005-16 | *Liocichla phoenicea* | GPS | 27.49 | 97.82 | Renner | Post-2000 | 2005 | Forest 600-1800m |
| 2005-20 | *Megalaima asiatica* | GPS | 27.49 | 97.82 | Renner | Post-2000 | 2005 | Forest 600-1800m |
| 2005-24 | *Myiomela leucura* | GPS | 27.49 | 97.82 | Renner | Post-2000 | 2005 | Forest 600-1800m |
| 2005-26 | *Myiomela leucura* | GPS | 27.49 | 97.82 | Renner | Post-2000 | 2005 | Forest 600-1800m |
| 2005-32 | *Myiomela leucura* | GPS | 27.49 | 97.82 | Renner | Post-2000 | 2005 | Forest 600-1800m |
| 2005-4 | *Myiomela leucura* | GPS | 27.49 | 97.82 | Renner | Post-2000 | 2005 | Forest 600-1800m |
| 2005-5 | *Niltava macgrigoriae* | GPS | 27.49 | 97.82 | Renner | Post-2000 | 2005 | Forest 600-1800m |
| 2005-29 | *Niltava rubicoloides* | GPS | 27.49 | 97.82 | Renner | Post-2000 | 2005 | Forest 600-1800m |
| 2005-43 | *Niltava rubicoloides* | GPS | 27.49 | 97.82 | Renner | Post-2000 | 2005 | Forest 600-1800m |
| 2005-44 | *Pycnonotus jocosus* | GPS | 27.49 | 97.82 | Renner | Post-2000 | 2005 | Forest 600-1800m |
| 2005-12 | *Sasia ochracea* | GPS | 27.49 | 97.82 | Renner | Post-2000 | 2005 | Forest 600-1800m |
| 2005-11 | *Serilophus lunatus* | GPS | 27.49 | 97.82 | Renner | Post-2000 | 2005 | Forest 600-1800m |
| 2005-14 | *Serilophus lunatus* | GPS | 27.49 | 97.82 | Renner | Post-2000 | 2005 | Forest 600-1800m |
| 2005-15 | *Serilophus lunatus* | GPS | 27.49 | 97.82 | Renner | Post-2000 | 2005 | Forest 600-1800m |
| 2005-41 | *Stachyris chrysaea* | GPS | 27.49 | 97.82 | Renner | Post-2000 | 2005 | Forest 600-1800m |
| 2005-33 | *Terpsiphone paradisi* | GPS | 27.49 | 97.82 | Renner | Post-2000 | 2005 | Forest 600-1800m |
| 2005-38 | *Terpsiphone paradisi* | GPS | 27.49 | 97.82 | Renner | Post-2000 | 2005 | Forest 600-1800m |
| 2005-8 | *Terpsiphone paradisi* | GPS | 27.49 | 97.82 | Renner | Post-2000 | 2005 | Forest 600-1800m |
| 2005-28 | *Turdus boulboul* | GPS | 27.49 | 97.82 | Renner | Post-2000 | 2005 | Forest 600-1800m |
| 2005-1 | *Zoothera citrina* | GPS | 27.49 | 97.82 | Renner | Post-2000 | 2005 | Forest 600-1800m |
| 2005-40 | *Zoothera citrina* | GPS | 27.49 | 97.82 | Renner | Post-2000 | 2005 | Forest 600-1800m |
| 2006-3269 | *Ficedula monileger* | GPS | 27.47 | 97.72 | Renner | Post-2000 | 2006 | Forest 600-1800m |
| 2006-3267 | *Garrulax squamatus* | GPS | 27.47 | 97.72 | Renner | Post-2000 | 2006 | Forest 600-1800m |
| 2006-3268 | *Garrulax squamatus* | GPS | 27.47 | 97.72 | Renner | Post-2000 | 2006 | Forest 600-1800m |
| 2006-3270 | *Niltava grandis* | GPS | 27.47 | 97.72 | Renner | Post-2000 | 2006 | Forest 600-1800m |
| 2006-3271 | *Pomatorhinus ferruginosus* | GPS | 27.47 | 97.72 | Renner | Post-2000 | 2006 | Forest 600-1800m |
| 2006-3265 | *Ficedula sp.* | GPS | 27.41 | 97.67 | Renner | Post-2000 | 2006 | Forest 600-1800m |
| 2006-3266 | *Pellorneum sp.* | GPS | 27.41 | 97.67 | Renner | Post-2000 | 2006 | Forest 600-1800m |
| 2006-3279 | *Abroscopus albogularis* | GPS | 27.48 | 97.82 | Renner | Post-2000 | 2006 | Forest 600-1800m |
| 2006-3317 | *Abroscopus albogularis* | GPS | 27.48 | 97.82 | Renner | Post-2000 | 2006 | Forest 600-1800m |
| 2006-3277 | *Accipiter virgatus* | GPS | 27.48 | 97.82 | Renner | Post-2000 | 2006 | Forest 600-1800m |
| 2006-3273 | *Alcippe morrisonia* | GPS | 27.48 | 97.82 | Renner | Post-2000 | 2006 | Forest 600-1800m |
| 2006-3274 | *Alcippe morrisonia* | GPS | 27.48 | 97.82 | Renner | Post-2000 | 2006 | Forest 600-1800m |
| 2006-3275 | *Alcippe morrisonia* | GPS | 27.48 | 97.82 | Renner | Post-2000 | 2006 | Forest 600-1800m |
| 2006-3284 | *Alcippe morrisonia* | GPS | 27.48 | 97.82 | Renner | Post-2000 | 2006 | Forest 600-1800m |
| 2006-3320 | *Alcippe morrisonia* | GPS | 27.48 | 97.82 | Renner | Post-2000 | 2006 | Forest 600-1800m |
| 2006-3309 | *Alcippe rufogularis* | GPS | 27.48 | 97.82 | Renner | Post-2000 | 2006 | Forest 600-1800m |
| 2006-3310 | *Alcippe rufogularis* | GPS | 27.48 | 97.82 | Renner | Post-2000 | 2006 | Forest 600-1800m |
| 2006-3311 | *Alcippe rufogularis* | GPS | 27.48 | 97.82 | Renner | Post-2000 | 2006 | Forest 600-1800m |
| 2006-3319 | *Alcippe rufogularis* | GPS | 27.48 | 97.82 | Renner | Post-2000 | 2006 | Forest 600-1800m |
| 2006-3307 | *Alophoixus flaveolus* | GPS | 27.48 | 97.82 | Renner | Post-2000 | 2006 | Forest 600-1800m |
| 2006-3321 | *Alophoixus flaveolus* | GPS | 27.48 | 97.82 | Renner | Post-2000 | 2006 | Forest 600-1800m |
| 2006-3296 | *Arachnothera longirostris* | GPS | 27.48 | 97.82 | Renner | Post-2000 | 2006 | Forest 600-1800m |
| 2006-3322 | *Blythipicus pyrrhotis* | GPS | 27.48 | 97.82 | Renner | Post-2000 | 2006 | Forest 600-1800m |
| 2006-3290 | *Chloropsis hardwickii* | GPS | 27.48 | 97.82 | Renner | Post-2000 | 2006 | Forest 600-1800m |
| 2006-3286 | *Dendrocitta frontalis* | GPS | 27.48 | 97.82 | Renner | Post-2000 | 2006 | Forest 600-1800m |
| 2006-3299 | *Dendrocitta frontalis* | GPS | 27.48 | 97.82 | Renner | Post-2000 | 2006 | Forest 600-1800m |
| 2006-3316 | *Dendrocitta frontalis* | GPS | 27.48 | 97.82 | Renner | Post-2000 | 2006 | Forest 600-1800m |
| 2006-3282 | *Ficedula strophiata* | GPS | 27.48 | 97.82 | Renner | Post-2000 | 2006 | Forest 600-1800m |
| 2006-3285 | *Garrulax leucolophus* | GPS | 27.48 | 97.82 | Renner | Post-2000 | 2006 | Forest 600-1800m |
| 2006-3318 | *Jabouilleia naungmungensis* | GPS | 27.48 | 97.82 | Renner | Post-2000 | 2006 | Forest 600-1800m |
| 2006-3297 | *Megalaima asiatica* | GPS | 27.48 | 97.82 | Renner | Post-2000 | 2006 | Forest 600-1800m |
| 2006-3272 | *Napothera brevicaudata* | GPS | 27.48 | 97.82 | Renner | Post-2000 | 2006 | Forest 600-1800m |
| 2006-3303 | *Napothera epilepidota* | GPS | 27.48 | 97.82 | Renner | Post-2000 | 2006 | Forest 600-1800m |
| 2006-3323 | *Napothera epilepidota* | GPS | 27.48 | 97.82 | Renner | Post-2000 | 2006 | Forest 600-1800m |
| 2006-3295 | *Pellorneum sp.* | GPS | 27.48 | 97.82 | Renner | Post-2000 | 2006 | Forest 600-1800m |
| 2006-3302 | *Pellorneum sp.* | GPS | 27.48 | 97.82 | Renner | Post-2000 | 2006 | Forest 600-1800m |
| 2006-3315 | *Pellorneum sp.* | GPS | 27.48 | 97.82 | Renner | Post-2000 | 2006 | Forest 600-1800m |
| 2006-3287 | *Pellorneum tickelli* | GPS | 27.48 | 97.82 | Renner | Post-2000 | 2006 | Forest 600-1800m |
| 2006-3288 | *Pellorneum tickelli* | GPS | 27.48 | 97.82 | Renner | Post-2000 | 2006 | Forest 600-1800m |
| 2006-3278 | *Phylloscopus cantator* | GPS | 27.48 | 97.82 | Renner | Post-2000 | 2006 | Forest 600-1800m |
| 2006-3308 | *Phylloscopus cantator* | GPS | 27.48 | 97.82 | Renner | Post-2000 | 2006 | Forest 600-1800m |
| 2006-3298 | *Pseudominla castaneceps* | GPS | 27.48 | 97.82 | Renner | Post-2000 | 2006 | Forest 600-1800m |
| 2006-3276 | *Rhipidura albicollis* | GPS | 27.48 | 97.82 | Renner | Post-2000 | 2006 | Forest 600-1800m |
| 2006-3306 | *Rhipidura albicollis* | GPS | 27.48 | 97.82 | Renner | Post-2000 | 2006 | Forest 600-1800m |
| 2006-3301 | *Sasia ochracea* | GPS | 27.48 | 97.82 | Renner | Post-2000 | 2006 | Forest 600-1800m |
| 2006-3305 | *Sasia ochracea* | GPS | 27.48 | 97.82 | Renner | Post-2000 | 2006 | Forest 600-1800m |
| 2006-3325 | *Seicercus burkii* | GPS | 27.48 | 97.82 | Renner | Post-2000 | 2006 | Forest 600-1800m |
| 2006-3289 | *Seicercus poliogenys* | GPS | 27.48 | 97.82 | Renner | Post-2000 | 2006 | Forest 600-1800m |
| 2006-3280 | *Seicercus sp.* | GPS | 27.48 | 97.82 | Renner | Post-2000 | 2006 | Forest 600-1800m |
| 2006-3283 | *Seicercus sp.* | GPS | 27.48 | 97.82 | Renner | Post-2000 | 2006 | Forest 600-1800m |
| 2006-3312 | *Stachyris nigriceps* | GPS | 27.48 | 97.82 | Renner | Post-2000 | 2006 | Forest 600-1800m |
| 2006-3291 | *Stachyris oglei* | GPS | 27.48 | 97.82 | Renner | Post-2000 | 2006 | Forest 600-1800m |
| 2006-3292 | *Stachyris oglei* | GPS | 27.48 | 97.82 | Renner | Post-2000 | 2006 | Forest 600-1800m |
| 2006-3300 | *Stachyris oglei* | GPS | 27.48 | 97.82 | Renner | Post-2000 | 2006 | Forest 600-1800m |
| 2006-3281 | *Tesia olivea* | GPS | 27.48 | 97.82 | Renner | Post-2000 | 2006 | Forest 600-1800m |
| 2006-3293 | *Yuhina castaneiceps* | GPS | 27.48 | 97.82 | Renner | Post-2000 | 2006 | Forest 600-1800m |
| 2006-3294 | *Yuhina castaneiceps* | GPS | 27.48 | 97.82 | Renner | Post-2000 | 2006 | Forest 600-1800m |
| 2006-3324 | *Yuhina castaneiceps* | GPS | 27.48 | 97.82 | Renner | Post-2000 | 2006 | Forest 600-1800m |
| 2006-3304 | *Yuhina zantholeuca* | GPS | 27.48 | 97.82 | Renner | Post-2000 | 2006 | Forest 600-1800m |
| 2006-3313 | *Yuhina zantholeuca* | GPS | 27.48 | 97.82 | Renner | Post-2000 | 2006 | Forest 600-1800m |
| 2006-3314 | *Yuhina zantholeuca* | GPS | 27.48 | 97.82 | Renner | Post-2000 | 2006 | Forest 600-1800m |
| 2006-3347 | *Abroscopus albogularis* | GPS | 27.29 | 97.86 | Renner | Post-2000 | 2006 | Secondary forest 600-1800m |
| 2006-3365 | *Aethopyga saturata* | GPS | 27.29 | 97.86 | Renner | Post-2000 | 2006 | Secondary forest 600-1800m |
| 2006-3362 | *Alcedo hercules* | GPS | 27.29 | 97.86 | Renner | Post-2000 | 2006 | Secondary forest 600-1800m |
| 2006-3363 | *Arachnothera magna* | GPS | 27.29 | 97.86 | Renner | Post-2000 | 2006 | Secondary forest 600-1800m |
| 2006-3342 | *Cissa chinensis* | GPS | 27.29 | 97.86 | Renner | Post-2000 | 2006 | Secondary forest 600-1800m |
| 2006-3341 | *Cyornis sp.* | GPS | 27.29 | 97.86 | Renner | Post-2000 | 2006 | Secondary forest 600-1800m |
| 2006-3337 | *Dendrocitta frontalis* | GPS | 27.29 | 97.86 | Renner | Post-2000 | 2006 | Secondary forest 600-1800m |
| 2006-3352 | *Dendrocitta frontalis* | GPS | 27.29 | 97.86 | Renner | Post-2000 | 2006 | Secondary forest 600-1800m |
| 2006-3345 | *Enicurus leuschenaulti* | GPS | 27.29 | 97.86 | Renner | Post-2000 | 2006 | Secondary forest 600-1800m |
| 2006-3326 | *Ficedula hyperythra* | GPS | 27.29 | 97.86 | Renner | Post-2000 | 2006 | Secondary forest 600-1800m |
| 2006-3340 | *Ficedula hyperythra* | GPS | 27.29 | 97.86 | Renner | Post-2000 | 2006 | Secondary forest 600-1800m |
| 2006-3346 | *Ficedula hyperythra* | GPS | 27.29 | 97.86 | Renner | Post-2000 | 2006 | Secondary forest 600-1800m |
| 2006-3332 | *Garrulax gularis* | GPS | 27.29 | 97.86 | Renner | Post-2000 | 2006 | Secondary forest 600-1800m |
| 2006-3333 | *Garrulax gularis* | GPS | 27.29 | 97.86 | Renner | Post-2000 | 2006 | Secondary forest 600-1800m |
| 2006-3334 | *Garrulax gularis* | GPS | 27.29 | 97.86 | Renner | Post-2000 | 2006 | Secondary forest 600-1800m |
| 2006-3339 | *Garrulax gularis* | GPS | 27.29 | 97.86 | Renner | Post-2000 | 2006 | Secondary forest 600-1800m |
| 2006-3335 | *Garrulax leucolophus* | GPS | 27.29 | 97.86 | Renner | Post-2000 | 2006 | Secondary forest 600-1800m |
| 2006-3357 | *Garrulax monileger* | GPS | 27.29 | 97.86 | Renner | Post-2000 | 2006 | Secondary forest 600-1800m |
| 2006-3358 | *Garrulax monileger* | GPS | 27.29 | 97.86 | Renner | Post-2000 | 2006 | Secondary forest 600-1800m |
| 2006-3364 | *Garrulax nuchalis* | GPS | 27.29 | 97.86 | Renner | Post-2000 | 2006 | Secondary forest 600-1800m |
| 2006-3328 | *Garrulax rufogularis* | GPS | 27.29 | 97.86 | Renner | Post-2000 | 2006 | Secondary forest 600-1800m |
| 2006-3343 | *Garrulax rufogularis* | GPS | 27.29 | 97.86 | Renner | Post-2000 | 2006 | Secondary forest 600-1800m |
| 2006-3354 | *Garrulax rufogularis* | GPS | 27.29 | 97.86 | Renner | Post-2000 | 2006 | Secondary forest 600-1800m |
| 2006-3355 | *Garrulax rufogularis* | GPS | 27.29 | 97.86 | Renner | Post-2000 | 2006 | Secondary forest 600-1800m |
| 2006-3367 | *Jabouilleia naungmungensis* | GPS | 27.29 | 97.86 | Renner | Post-2000 | 2006 | Secondary forest 600-1800m |
| 2006-3327 | *Leiothrix lutea* | GPS | 27.29 | 97.86 | Renner | Post-2000 | 2006 | Secondary forest 600-1800m |
| 2006-3331 | *Leiothrix lutea* | GPS | 27.29 | 97.86 | Renner | Post-2000 | 2006 | Secondary forest 600-1800m |
| 2006-3344 | *Napothera epilepidota* | GPS | 27.29 | 97.86 | Renner | Post-2000 | 2006 | Secondary forest 600-1800m |
| 2006-3361 | *Napothera epilepidota* | GPS | 27.29 | 97.86 | Renner | Post-2000 | 2006 | Secondary forest 600-1800m |
| 2006-3360 | *Niltava rubicoloides* | GPS | 27.29 | 97.86 | Renner | Post-2000 | 2006 | Secondary forest 600-1800m |
| 2006-3348 | *Niltava sundara* | GPS | 27.29 | 97.86 | Renner | Post-2000 | 2006 | Secondary forest 600-1800m |
| 2006-3359 | *Otus sunia* | GPS | 27.29 | 97.86 | Renner | Post-2000 | 2006 | Secondary forest 600-1800m |
| 2006-3350 | *Paradoxornis ruficeps* | GPS | 27.29 | 97.86 | Renner | Post-2000 | 2006 | Secondary forest 600-1800m |
| 2006-3351 | *Paradoxornis ruficeps* | GPS | 27.29 | 97.86 | Renner | Post-2000 | 2006 | Secondary forest 600-1800m |
| 2006-3329 | *Pellorneum sp.* | GPS | 27.29 | 97.86 | Renner | Post-2000 | 2006 | Secondary forest 600-1800m |
| 2006-3330 | *Pellorneum sp.* | GPS | 27.29 | 97.86 | Renner | Post-2000 | 2006 | Secondary forest 600-1800m |
| 2006-3366 | *Phylloscopus cantator* | GPS | 27.29 | 97.86 | Renner | Post-2000 | 2006 | Secondary forest 600-1800m |
| 2006-3349 | *Pomatorhinus ochraceiceps* | GPS | 27.29 | 97.86 | Renner | Post-2000 | 2006 | Secondary forest 600-1800m |
| 2006-3338 | *Seicercus affinis* | GPS | 27.29 | 97.86 | Renner | Post-2000 | 2006 | Secondary forest 600-1800m |
| 2006-3353 | *Seicercus poliogenys* | GPS | 27.29 | 97.86 | Renner | Post-2000 | 2006 | Secondary forest 600-1800m |
| 2006-3356 | *Seicercus poliogenys* | GPS | 27.29 | 97.86 | Renner | Post-2000 | 2006 | Secondary forest 600-1800m |
| 2006-3336 | *Serilophus lunatus* | *GPS* | 27.29 | 97.86 | Renner | Post-2000 | 2006 | Secondary forest 600-1800m |

**Table S2.** Model estimates of GLM (Table 3 in text).

| **Model Name** | **Response** | **Coefficients** | **Estimate** | **SE** | **t-value** | ***p*** |
| --- | --- | --- | --- | --- | --- | --- |
| s1 | species richness | (Intercept) | 1.322 | NA | NA | NA |
| s1 | species richness | locality_simplifiedBabwa | -0.770 | NA | NA | NA |
| s1 | species richness | locality_simplifiedGawai | -1.660 | NA | NA | NA |
| s1 | species richness | locality_simplifiedGawlai | 0.710 | NA | NA | NA |
| s1 | species richness | locality_simplifiedHkamko | -1.987 | NA | NA | NA |
| s1 | species richness | locality_simplifiedHtingnan | -2.413 | NA | NA | NA |
| s1 | species richness | locality_simplifiedKankiu | -0.845 | NA | NA | NA |
| s1 | species richness | locality_simplifiedLonnat | -1.023 | NA | NA | NA |
| s1 | species richness | locality_simplifiedMasa | -0.961 | NA | NA | NA |
| s1 | species richness | locality_simplifiedMatsatap | 0.157 | NA | NA | NA |
| s1 | species richness | locality_simplifiedNagwa | -1.412 | NA | NA | NA |
| s1 | species richness | locality_simplifiedNamTamaiRiver | -1.021 | NA | NA | NA |
| s1 | species richness | locality_simplifiedNamTi | -0.389 | NA | NA | NA |
| s1 | species richness | locality_simplifiedNamTisangriver | -1.660 | NA | NA | NA |
| s1 | species richness | locality_simplifiedNaunghkai | -0.544 | NA | NA | NA |
| s1 | species richness | locality_simplifiedNaungMung | 0.333 | NA | NA | NA |
| s1 | species richness | locality_simplifiedPangnamdim | -0.998 | NA | NA | NA |
| s1 | species richness | locality_simplifiedPutao | -1.799 | NA | NA | NA |
| s1 | species richness | locality_simplifiedRatnamhti | -2.192 | NA | NA | NA |
| s1 | species richness | locality_simplifiedSeinghku | 0.903 | NA | NA | NA |
| s1 | species richness | locality_simplifiedShinsanku | -1.183 | NA | NA | NA |
| s1 | species richness | locality_simplifiedTahaundam | -0.954 | NA | NA | NA |
| s1 | species richness | locality_simplifiedTazundam | -1.498 | NA | NA | NA |
| s1 | species richness | locality_simplifiedThalatu | -1.021 | NA | NA | NA |
| s1 | species richness | locality_simplifiedURingGa | 1.065 | NA | NA | NA |
| s1 | species richness | locality_simplifiedWangsewan | 0.340 | NA | NA | NA |
| s1 | species richness | Habitat2016Forest6001800m | 1.568 | NA | NA | NA |
| s1 | species richness | Habitat2016Forest600m | -0.845 | NA | NA | NA |
| s1 | species richness | Habitat2016Iceglacier | -0.623 | NA | NA | NA |
| s1 | species richness | Habitat2016na | -1.021 | NA | NA | NA |
| s1 | species richness | Habitat2016PineRhododendron | 0.502 | NA | NA | NA |
| s1 | species richness | Habitat2016Rockboulder | 0.803 | NA | NA | NA |
| s1 | species richness | Habitat2016Secondaryforest6001800m | NA | NA | NA | NA |
| s1 | species richness | Habitat2016Secondaryforest600m | NA | NA | NA | NA |
| s1 | species richness | Habitat2016Settlement | 1.708 | NA | NA | NA |
| s1 | species richness | Habitat2016ShrubBushFern | -1.178 | NA | NA | NA |
| s1 | species richness | Habitat2016Streambed | NA | NA | NA | NA |
| s1 | species richness | Habitat1989Forest6001800m | NA | NA | NA | NA |
| s1 | species richness | Habitat1989Forest600m | 0.421 | NA | NA | NA |
| s1 | species richness | Habitat1989Iceglacier | -1.125 | NA | NA | NA |
| s1 | species richness | Habitat1989na | NA | NA | NA | NA |
| s1 | species richness | Habitat1989Paddyfield | NA | NA | NA | NA |
| s1 | species richness | Habitat1989PineRhododendron | -1.347 | NA | NA | NA |
| s1 | species richness | Habitat1989Rockboulder | -1.523 | NA | NA | NA |
| s1 | species richness | Habitat1989Secondaryforest600m | NA | NA | NA | NA |
| s1 | species richness | Habitat1989Settlement | NA | NA | NA | NA |
| s1 | species richness | Habitat1989ShrubBushFern | NA | NA | NA | NA |
| s1 | species richness | Habitat1989Streambed | NA | NA | NA | NA |
| s1 | species richness | TypeRecent20012006 | -0.753 | NA | NA | NA |
| s2 | species richness | (Intercept) | 0.833 | 0.273 | 3.051 | 0.038 |
| s2 | species richness | locality_simplifiedBabwa | 0.523 | 0.863 | 0.605 | 0.578 |
| s2 | species richness | locality_simplifiedGawai | -1.171 | 1.092 | -1.072 | 0.344 |
| s2 | species richness | locality_simplifiedGawlai | -0.508 | 0.863 | -0.589 | 0.588 |
| s2 | species richness | locality_simplifiedHkamko | -1.498 | 0.863 | -1.735 | 0.158 |
| s2 | species richness | locality_simplifiedHtingnan | -1.924 | 0.863 | -2.229 | 0.090 |
| s2 | species richness | locality_simplifiedKankiu | -0.356 | 0.546 | -0.651 | 0.550 |
| s2 | species richness | locality_simplifiedLonnat | -0.534 | 1.092 | -0.489 | 0.651 |
| s2 | species richness | locality_simplifiedMasa | -0.472 | 1.092 | -0.432 | 0.688 |
| s2 | species richness | locality_simplifiedMatsatap | 0.646 | 1.280 | 0.505 | 0.640 |
| s2 | species richness | locality_simplifiedNagwa | 1.491 | 1.280 | 1.165 | 0.309 |
| s2 | species richness | locality_simplifiedNamTamaiRiver | -0.532 | 0.546 | -0.974 | 0.385 |
| s2 | species richness | locality_simplifiedNamTi | 0.101 | 1.092 | 0.092 | 0.931 |
| s2 | species richness | locality_simplifiedNamTisangriver | -1.171 | 1.092 | -1.072 | 0.344 |
| s2 | species richness | locality_simplifiedNaunghkai | -0.055 | 0.546 | -0.100 | 0.925 |
| s2 | species richness | locality_simplifiedNaungMung | 0.822 | 1.092 | 0.753 | 0.493 |
| s2 | species richness | locality_simplifiedPangnamdim | -0.508 | 0.863 | -0.589 | 0.588 |
| s2 | species richness | locality_simplifiedPutao | 0.398 | 0.546 | 0.728 | 0.507 |
| s2 | species richness | locality_simplifiedRatnamhti | -1.702 | 0.863 | -1.972 | 0.120 |
| s2 | species richness | locality_simplifiedSeinghku | 0.206 | 0.438 | 0.471 | 0.663 |
| s2 | species richness | locality_simplifiedShinsanku | -0.693 | 1.092 | -0.635 | 0.560 |
| s2 | species richness | locality_simplifiedTahaundam | -0.465 | 0.863 | -0.538 | 0.619 |
| s2 | species richness | locality_simplifiedTazundam | -1.009 | 0.863 | -1.169 | 0.307 |
| s2 | species richness | locality_simplifiedThalatu | -0.532 | 0.546 | -0.974 | 0.385 |
| s2 | species richness | locality_simplifiedURingGa | -0.014 | 1.092 | -0.013 | 0.990 |
| s2 | species richness | locality_simplifiedWangsewan | 1.074 | 0.875 | 1.227 | 0.287 |
| s2 | species richness | Habitat1989Forest6001800m | 1.568 | 0.669 | 2.345 | 0.079 |
| s2 | species richness | Habitat1989Forest600m | -0.425 | 0.946 | -0.449 | 0.677 |
| s2 | species richness | Habitat1989Iceglacier | -0.197 | 0.484 | -0.407 | 0.705 |
| s2 | species richness | Habitat1989na | -0.532 | 0.546 | -0.974 | 0.385 |
| s2 | species richness | Habitat1989Paddyfield | NA | NA | NA | NA |
| s2 | species richness | Habitat1989PineRhododendron | -0.356 | 0.546 | -0.651 | 0.550 |
| s2 | species richness | Habitat1989Rockboulder | -0.199 | 0.413 | -0.482 | 0.655 |
| s2 | species richness | Habitat1989Secondaryforest600m | NA | NA | NA | NA |
| s2 | species richness | Habitat1989Settlement | 1.708 | 0.946 | 1.806 | 0.145 |
| s2 | species richness | Habitat1989ShrubBushFern | -1.178 | 1.158 | -1.017 | 0.367 |
| s2 | species richness | Habitat1989Streambed | NA | NA | NA | NA |
| s2 | species richness | TypeRecent20012006 | -0.753 | 0.669 | -1.127 | 0.323 |
| s3 | species richness | (Intercept) | 1.252 | 0.300 | 4.177 | 0.014 |
| s3 | species richness | locality_simplifiedBabwa | 0.554 | 0.614 | 0.902 | 0.418 |
| s3 | species richness | locality_simplifiedGawai | -1.450 | 0.697 | -2.081 | 0.106 |
| s3 | species richness | locality_simplifiedGawlai | 0.780 | 0.643 | 1.213 | 0.292 |
| s3 | species richness | locality_simplifiedHkamko | -1.777 | 0.519 | -3.422 | 0.027 |
| s3 | species richness | locality_simplifiedHtingnan | -2.203 | 0.519 | -4.243 | 0.013 |
| s3 | species richness | locality_simplifiedKankiu | -0.775 | 0.445 | -1.743 | 0.156 |
| s3 | species richness | locality_simplifiedLonnat | -0.813 | 0.697 | -1.167 | 0.308 |
| s3 | species richness | locality_simplifiedMasa | -0.751 | 0.697 | -1.078 | 0.342 |
| s3 | species richness | locality_simplifiedMatsatap | 0.437 | 0.758 | 0.576 | 0.595 |
| s3 | species richness | locality_simplifiedNagwa | -0.781 | 0.697 | -1.121 | 0.325 |
| s3 | species richness | locality_simplifiedNamTamaiRiver | -0.881 | 0.379 | -2.323 | 0.081 |
| s3 | species richness | locality_simplifiedNamTi | -0.179 | 0.697 | -0.256 | 0.810 |
| s3 | species richness | locality_simplifiedNamTisangriver | -1.450 | 0.697 | -2.081 | 0.106 |
| s3 | species richness | locality_simplifiedNaunghkai | -0.474 | 0.445 | -1.066 | 0.347 |
| s3 | species richness | locality_simplifiedNaungMung | 0.613 | 0.600 | 1.023 | 0.364 |
| s3 | species richness | locality_simplifiedPangnamdim | -0.928 | 0.643 | -1.443 | 0.223 |
| s3 | species richness | locality_simplifiedPutao | -1.729 | 0.643 | -2.690 | 0.055 |
| s3 | species richness | locality_simplifiedRatnamhti | -1.981 | 0.519 | -3.816 | 0.019 |
| s3 | species richness | locality_simplifiedSeinghku | 0.371 | 0.304 | 1.219 | 0.290 |
| s3 | species richness | locality_simplifiedShinsanku | -0.973 | 0.697 | -1.396 | 0.235 |
| s3 | species richness | locality_simplifiedTahaundam | -0.884 | 0.643 | -1.375 | 0.241 |
| s3 | species richness | locality_simplifiedTazundam | -1.428 | 0.643 | -2.221 | 0.091 |
| s3 | species richness | locality_simplifiedThalatu | -0.951 | 0.445 | -2.139 | 0.099 |
| s3 | species richness | locality_simplifiedURingGa | 1.135 | 0.643 | 1.765 | 0.152 |
| s3 | species richness | locality_simplifiedWangsewan | -0.973 | 0.697 | -1.396 | 0.235 |
| s3 | species richness | Habitat2016Forest6001800m | 1.428 | 0.379 | 3.766 | 0.020 |
| s3 | species richness | Habitat2016Forest600m | -0.705 | 0.379 | -1.859 | 0.137 |
| s3 | species richness | Habitat2016Iceglacier | -0.849 | 0.409 | -2.079 | 0.106 |
| s3 | species richness | Habitat2016na | -0.951 | 0.445 | -2.139 | 0.099 |
| s3 | species richness | Habitat2016PineRhododendron | -0.715 | 0.369 | -1.938 | 0.125 |
| s3 | species richness | Habitat2016Rockboulder | -0.451 | 0.379 | -1.190 | 0.300 |
| s3 | species richness | Habitat2016Secondaryforest6001800m | NA | NA | NA | NA |
| s3 | species richness | Habitat2016Secondaryforest600m | NA | NA | NA | NA |
| s3 | species richness | Habitat2016Settlement | 1.708 | 0.657 | 2.600 | 0.060 |
| s3 | species richness | Habitat2016ShrubBushFern | -1.388 | 0.697 | -1.993 | 0.117 |
| s3 | species richness | Habitat2016Streambed | NA | NA | NA | NA |
| s3 | species richness | TypeRecent20012006 | -0.753 | 0.464 | -1.622 | 0.180 |
| s4 | species richness | (Intercept) | 0.641 | 0.278 | 2.306 | 0.029 |
| s4 | species richness | Habitat2016Forest6001800m | 0.471 | 0.309 | 1.527 | 0.138 |
| s4 | species richness | Habitat2016Forest600m | -0.164 | 0.440 | -0.374 | 0.712 |
| s4 | species richness | Habitat2016Iceglacier | -0.053 | 0.440 | -0.121 | 0.904 |
| s4 | species richness | Habitat2016na | -0.340 | 0.556 | -0.612 | 0.546 |
| s4 | species richness | Habitat2016PineRhododendron | 0.019 | 0.393 | 0.049 | 0.961 |
| s4 | species richness | Habitat2016Rockboulder | 0.093 | 0.393 | 0.237 | 0.814 |
| s4 | species richness | Habitat2016Secondaryforest6001800m | 0.815 | 0.440 | 1.853 | 0.075 |
| s4 | species richness | Habitat2016Secondaryforest600m | -0.164 | 0.556 | -0.295 | 0.770 |
| s4 | species richness | Habitat2016Settlement | 0.416 | 0.341 | 1.220 | 0.233 |
| s4 | species richness | Habitat2016ShrubBushFern | -0.252 | 0.440 | -0.574 | 0.571 |
| s4 | species richness | Habitat2016Streambed | 0.137 | 0.556 | 0.246 | 0.808 |
| s5 | species richness | (Intercept) | 0.699 | 0.103 | 6.802 | 0.000 |
| s5 | species richness | TypeRecent20012006 | 0.391 | 0.151 | 2.587 | 0.014 |
| s6 | species richness | (Intercept) | 0.647 | 0.235 | 2.751 | 0.017 |
| s6 | species richness | locality_simplifiedBabwa | -0.045 | 0.706 | -0.064 | 0.950 |
| s6 | species richness | locality_simplifiedGawai | -0.170 | 0.706 | -0.241 | 0.813 |
| s6 | species richness | locality_simplifiedGawlai | 0.631 | 0.706 | 0.894 | 0.388 |
| s6 | species richness | locality_simplifiedHkamko | 0.256 | 0.706 | 0.362 | 0.723 |
| s6 | species richness | locality_simplifiedHtingnan | -0.170 | 0.706 | -0.241 | 0.813 |
| s6 | species richness | locality_simplifiedKankiu | -0.170 | 0.706 | -0.241 | 0.813 |
| s6 | species richness | locality_simplifiedLonnat | 0.467 | 0.706 | 0.661 | 0.520 |
| s6 | species richness | locality_simplifiedMasa | 0.529 | 0.706 | 0.749 | 0.467 |
| s6 | species richness | locality_simplifiedMatsatap | -0.346 | 0.706 | -0.491 | 0.632 |
| s6 | species richness | locality_simplifiedNagwa | 0.499 | 0.706 | 0.706 | 0.493 |
| s6 | species richness | locality_simplifiedNamTamaiRiver | 0.438 | 0.526 | 0.832 | 0.421 |
| s6 | species richness | locality_simplifiedNamTi | 1.101 | 0.706 | 1.559 | 0.143 |
| s6 | species richness | locality_simplifiedNamTisangriver | -0.170 | 0.706 | -0.241 | 0.813 |
| s6 | species richness | locality_simplifiedNaunghkai | 0.131 | 0.706 | 0.185 | 0.856 |
| s6 | species richness | locality_simplifiedNaungMung | 0.494 | 0.451 | 1.096 | 0.293 |
| s6 | species richness | locality_simplifiedPangnamdim | 0.631 | 0.706 | 0.894 | 0.388 |
| s6 | species richness | locality_simplifiedPutao | 0.206 | 0.526 | 0.392 | 0.701 |
| s6 | species richness | locality_simplifiedRatnamhti | 0.052 | 0.706 | 0.073 | 0.943 |
| s6 | species richness | locality_simplifiedSeinghku | 0.193 | 0.526 | 0.367 | 0.720 |
| s6 | species richness | locality_simplifiedShinsanku | 0.307 | 0.706 | 0.434 | 0.671 |
| s6 | species richness | locality_simplifiedTahaundam | 0.675 | 0.706 | 0.956 | 0.357 |
| s6 | species richness | locality_simplifiedTazundam | 0.131 | 0.706 | 0.185 | 0.856 |
| s6 | species richness | locality_simplifiedThalatu | 0.131 | 0.526 | 0.248 | 0.808 |
| s6 | species richness | locality_simplifiedURingGa | 0.986 | 0.706 | 1.396 | 0.186 |
| s6 | species richness | locality_simplifiedWangsewan | 0.307 | 0.706 | 0.434 | 0.671 |
| s7 | species richness | (Intercept) | 0.620 | 0.221 | 2.802 | 0.009 |
| s7 | species richness | Habitat1989Forest6001800m | 0.547 | 0.263 | 2.074 | 0.048 |
| s7 | species richness | Habitat1989Forest600m | 0.192 | 0.414 | 0.463 | 0.647 |
| s7 | species richness | Habitat1989Iceglacier | 0.119 | 0.414 | 0.286 | 0.777 |
| s7 | species richness | Habitat1989na | -0.319 | 0.542 | -0.588 | 0.561 |
| s7 | species richness | Habitat1989Paddyfield | 0.150 | 0.361 | 0.414 | 0.682 |
| s7 | species richness | Habitat1989PineRhododendron | -0.143 | 0.542 | -0.264 | 0.794 |
| s7 | species richness | Habitat1989Rockboulder | 0.145 | 0.332 | 0.438 | 0.665 |
| s7 | species richness | Habitat1989Secondaryforest600m | -0.143 | 0.542 | -0.264 | 0.794 |
| s7 | species richness | Habitat1989Settlement | 0.563 | 0.313 | 1.797 | 0.083 |
| s7 | species richness | Habitat1989ShrubBushFern | -0.231 | 0.414 | -0.558 | 0.582 |
| s7 | species richness | Habitat1989Streambed | 0.158 | 0.542 | 0.292 | 0.773 |
| a1 | relative abundance | (Intercept) | -1.332 | 3.124 | -0.426 | 0.670 |
| a1 | relative abundance | speciesAbroscopus schisticeps | -0.124 | 2.153 | -0.058 | 0.954 |
| a1 | relative abundance | speciesAccipiter virgatus | -0.681 | 1.529 | -0.446 | 0.656 |
| a1 | relative abundance | speciesAceros nipalensis | -0.374 | 2.030 | -0.184 | 0.854 |
| a1 | relative abundance | speciesAceros undulatus | -0.469 | 2.116 | -0.222 | 0.825 |
| a1 | relative abundance | speciesAcridotheres tristes | -0.284 | 2.092 | -0.136 | 0.892 |
| a1 | relative abundance | speciesActinodura egertoni | -0.374 | 2.030 | -0.184 | 0.854 |
| a1 | relative abundance | speciesActinodura waldeni | -0.553 | 3.564 | -0.155 | 0.877 |
| a1 | relative abundance | speciesAegithalos iouschistos | -0.124 | 2.153 | -0.058 | 0.954 |
| a1 | relative abundance | speciesAethopyga gouldiae | -0.124 | 2.153 | -0.058 | 0.954 |
| a1 | relative abundance | speciesAethopyga nipalensis | -0.247 | 1.631 | -0.152 | 0.879 |
| a1 | relative abundance | speciesAethopyga saturata | -0.023 | 0.963 | -0.024 | 0.981 |
| a1 | relative abundance | speciesAethopyga siparaja | -0.485 | 1.653 | -0.293 | 0.769 |
| a1 | relative abundance | speciesAlcedo athis | -0.382 | 3.266 | -0.117 | 0.907 |
| a1 | relative abundance | speciesAlcedo hercules | -0.382 | 2.006 | -0.190 | 0.849 |
| a1 | relative abundance | speciesAlcippe cinerea | 0.325 | 1.103 | 0.295 | 0.768 |
| a1 | relative abundance | speciesAlcippe cinereiceps | 0.336 | 1.683 | 0.200 | 0.842 |
| a1 | relative abundance | speciesAlcippe morrisonia | 0.368 | 0.981 | 0.375 | 0.708 |
| a1 | relative abundance | speciesAlcippe rufogularis | 0.328 | 1.044 | 0.314 | 0.754 |
| a1 | relative abundance | speciesAlcippe vinipectus | -0.355 | 1.849 | -0.192 | 0.848 |
| a1 | relative abundance | speciesAlophoixus flaveolus | 0.263 | 1.049 | 0.251 | 0.802 |
| a1 | relative abundance | speciesAnthus richardi | -0.330 | 1.566 | -0.211 | 0.833 |
| a1 | relative abundance | speciesAnthus roseatus | -0.177 | 2.222 | -0.080 | 0.937 |
| a1 | relative abundance | speciesArachnothera longirostris | -0.606 | 1.347 | -0.450 | 0.653 |
| a1 | relative abundance | speciesArachnothera magna | -0.001 | 1.048 | -0.001 | 0.999 |
| a1 | relative abundance | speciesArborophila atrogularis | -0.945 | 1.990 | -0.475 | 0.635 |
| a1 | relative abundance | speciesArborophila rufogularis | -0.351 | 2.003 | -0.175 | 0.861 |
| a1 | relative abundance | speciesBlythipicus pyrrhotis | -0.505 | 1.231 | -0.410 | 0.682 |
| a1 | relative abundance | speciesBrachypteryx hyperthyra | -0.485 | 1.653 | -0.293 | 0.769 |
| a1 | relative abundance | speciesBrachypteryx leucophris | -0.174 | 1.608 | -0.108 | 0.914 |
| a1 | relative abundance | speciesBrachypteryx sp. | -0.252 | 1.516 | -0.166 | 0.868 |
| a1 | relative abundance | speciesCeryle lugubris | -0.369 | 1.698 | -0.218 | 0.828 |
| a1 | relative abundance | speciesChaimarrornis leucocephalus | -0.345 | 2.527 | -0.136 | 0.892 |
| a1 | relative abundance | speciesChalcophaps indica | 0.109 | 1.669 | 0.066 | 0.948 |
| a1 | relative abundance | speciesCheliodorhynx hypoxantha | -0.374 | 2.030 | -0.184 | 0.854 |
| a1 | relative abundance | speciesChloropsis hardwickii | -0.945 | 1.990 | -0.475 | 0.635 |
| a1 | relative abundance | speciesCinclidium leucurum | 0.176 | 2.409 | 0.073 | 0.942 |
| a1 | relative abundance | speciesCinclus pallasii | -0.138 | 2.104 | -0.065 | 0.948 |
| a1 | relative abundance | speciesCissa chinensis | -0.263 | 1.130 | -0.233 | 0.816 |
| a1 | relative abundance | speciesCissa hypoleuca | 0.139 | 1.769 | 0.079 | 0.937 |
| a1 | relative abundance | speciesCollocalia brevirostris | -0.001 | 1.461 | 0.000 | 1.000 |
| a1 | relative abundance | speciesCoracina melaschistos | 0.086 | 1.702 | 0.051 | 0.960 |
| a1 | relative abundance | speciesCorvus macrorhynchus | -0.124 | 2.153 | -0.058 | 0.954 |
| a1 | relative abundance | speciesCulicicapa ceylonensis | -0.024 | 1.183 | -0.021 | 0.984 |
| a1 | relative abundance | speciesCyornis hainanus | -0.284 | 2.092 | -0.136 | 0.892 |
| a1 | relative abundance | speciesCyornis sp. | -0.382 | 2.006 | -0.190 | 0.849 |
| a1 | relative abundance | speciesDelichon dasypus | -0.374 | 2.030 | -0.184 | 0.854 |
| a1 | relative abundance | speciesDendrocitta frontalis | -0.026 | 1.102 | -0.024 | 0.981 |
| a1 | relative abundance | speciesDendrocopus cathparius | -0.124 | 2.153 | -0.058 | 0.954 |
| a1 | relative abundance | speciesDicrurus aeneus | -0.091 | 2.120 | -0.043 | 0.966 |
| a1 | relative abundance | speciesDicrurus paradiseus | -0.276 | 2.754 | -0.100 | 0.920 |
| a1 | relative abundance | speciesDicrurus remifer | -0.945 | 1.990 | -0.475 | 0.635 |
| a1 | relative abundance | speciesEnicurus leuschenaulti | -0.382 | 2.006 | -0.190 | 0.849 |
| a1 | relative abundance | speciesEnicurus maculatus | -0.351 | 2.003 | -0.175 | 0.861 |
| a1 | relative abundance | speciesEnicurus schistaceus | -0.138 | 2.104 | -0.065 | 0.948 |
| a1 | relative abundance | speciesEnicurus scouleri | -0.209 | 2.114 | -0.099 | 0.921 |
| a1 | relative abundance | speciesEurystomus orientalis | 0.319 | 1.569 | 0.203 | 0.839 |
| a1 | relative abundance | speciesFicedula hyperythra | -0.054 | 1.118 | -0.049 | 0.961 |
| a1 | relative abundance | speciesFicedula monileger | 0.061 | 0.998 | 0.061 | 0.951 |
| a1 | relative abundance | speciesFicedula sapphira | -0.352 | 1.784 | -0.198 | 0.843 |
| a1 | relative abundance | speciesFicedula sp. | -0.351 | 2.003 | -0.175 | 0.861 |
| a1 | relative abundance | speciesFicedula strophiata | -0.033 | 1.050 | -0.032 | 0.975 |
| a1 | relative abundance | speciesFicedula tricolor | -0.001 | 2.782 | 0.000 | 1.000 |
| a1 | relative abundance | speciesGarrulax affinis | -0.331 | 1.360 | -0.244 | 0.808 |
| a1 | relative abundance | speciesGarrulax erythrocephalus | 0.095 | 1.246 | 0.076 | 0.940 |
| a1 | relative abundance | speciesGarrulax gularis | 0.208 | 1.213 | 0.171 | 0.864 |
| a1 | relative abundance | speciesGarrulax leucolophus | -0.119 | 1.243 | -0.096 | 0.924 |
| a1 | relative abundance | speciesGarrulax monileger | -0.077 | 1.424 | -0.054 | 0.957 |
| a1 | relative abundance | speciesGarrulax nuchalis | -0.205 | 1.189 | -0.172 | 0.863 |
| a1 | relative abundance | speciesGarrulax ruficollis | -0.139 | 1.359 | -0.102 | 0.918 |
| a1 | relative abundance | speciesGarrulax rufogularis | -0.195 | 1.272 | -0.154 | 0.878 |
| a1 | relative abundance | speciesGarrulax squamatus | 0.370 | 1.807 | 0.205 | 0.838 |
| a1 | relative abundance | speciesGarrulax striatus | 0.217 | 1.202 | 0.181 | 0.857 |
| a1 | relative abundance | speciesGarrulax subunicolor | -0.351 | 1.785 | -0.196 | 0.844 |
| a1 | relative abundance | speciesGecinulus grantia | -0.336 | 3.006 | -0.112 | 0.911 |
| a1 | relative abundance | speciesGlaucidium brodiei | -0.471 | 1.458 | -0.323 | 0.747 |
| a1 | relative abundance | speciesGlaucidium cuculoides | -0.295 | 1.570 | -0.188 | 0.851 |
| a1 | relative abundance | speciesHarpactes erythrocephalus | -0.108 | 1.244 | -0.087 | 0.931 |
| a1 | relative abundance | speciesHemipus picatus | -0.448 | 2.333 | -0.192 | 0.848 |
| a1 | relative abundance | speciesHemixos flavala | -0.444 | 1.391 | -0.319 | 0.750 |
| a1 | relative abundance | speciesHeterophasia picaoides | -0.249 | 1.221 | -0.204 | 0.839 |
| a1 | relative abundance | speciesHeterophasia pulchella | 0.314 | 1.380 | 0.228 | 0.820 |
| a1 | relative abundance | speciesHirundo rustica | 0.086 | 1.702 | 0.051 | 0.960 |
| a1 | relative abundance | speciesHirundo striolata | -0.374 | 3.281 | -0.114 | 0.909 |
| a1 | relative abundance | speciesHypsipetes mcclelandi | -0.111 | 1.283 | -0.087 | 0.931 |
| a1 | relative abundance | speciesIthaginis cruentus | 0.783 | 2.741 | 0.286 | 0.775 |
| a1 | relative abundance | speciesJabouilleia naungmungensis | -0.195 | 1.272 | -0.154 | 0.878 |
| a1 | relative abundance | speciesJynx torquilla | 0.319 | 1.569 | 0.203 | 0.839 |
| a1 | relative abundance | speciesLanius schach | -0.284 | 2.092 | -0.136 | 0.892 |
| a1 | relative abundance | speciesLeiothrix argentauris | -0.096 | 1.087 | -0.089 | 0.929 |
| a1 | relative abundance | speciesLeiothrix lutea | -0.005 | 1.434 | -0.003 | 0.997 |
| a1 | relative abundance | speciesLeucosticte nemoricola | -0.124 | 2.153 | -0.058 | 0.954 |
| a1 | relative abundance | speciesLiocichla phoenicea | -0.643 | 1.532 | -0.420 | 0.674 |
| a1 | relative abundance | speciesLophophorus impejanus | -0.357 | 2.120 | -0.168 | 0.866 |
| a1 | relative abundance | speciesLophophorus sclateri | -0.357 | 2.120 | -0.168 | 0.866 |
| a1 | relative abundance | speciesLophura leucomelanus | -0.336 | 1.547 | -0.218 | 0.828 |
| a1 | relative abundance | speciesMacropygia unchall | -0.374 | 2.030 | -0.184 | 0.854 |
| a1 | relative abundance | speciesMalacocincla abbotti | -0.945 | 1.990 | -0.475 | 0.635 |
| a1 | relative abundance | speciesMegalaima asiatica | -0.485 | 1.653 | -0.293 | 0.769 |
| a1 | relative abundance | speciesMegalaima franklinii | 0.468 | 1.494 | 0.313 | 0.754 |
| a1 | relative abundance | speciesMegalaima virens | -0.363 | 1.530 | -0.237 | 0.813 |
| a1 | relative abundance | speciesMelanochlora sultanea | -0.374 | 2.030 | -0.184 | 0.854 |
| a1 | relative abundance | speciesMicropternus brachyurus | -0.276 | 2.754 | -0.100 | 0.920 |
| a1 | relative abundance | speciesMinla ignotincta | -0.945 | 1.990 | -0.475 | 0.635 |
| a1 | relative abundance | speciesMotacilla alba | 0.319 | 1.569 | 0.203 | 0.839 |
| a1 | relative abundance | speciesMotacilla citriola | -0.374 | 2.030 | -0.184 | 0.854 |
| a1 | relative abundance | speciesMuscicapa sibirica | -0.345 | 2.527 | -0.136 | 0.892 |
| a1 | relative abundance | speciesMycerobas affinis | -0.345 | 2.527 | -0.136 | 0.892 |
| a1 | relative abundance | speciesMycerobas carnipes | -0.345 | 2.527 | -0.136 | 0.892 |
| a1 | relative abundance | speciesMyiomela leucura | -0.103 | 1.438 | -0.072 | 0.943 |
| a1 | relative abundance | speciesMyophonus caeruleus | -0.352 | 2.160 | -0.163 | 0.871 |
| a1 | relative abundance | speciesNapothera brevicaudata | 0.043 | 1.191 | 0.036 | 0.971 |
| a1 | relative abundance | speciesNapothera epilepidota | -0.012 | 1.107 | -0.011 | 0.991 |
| a1 | relative abundance | speciesNiltava grandis | -0.042 | 0.967 | -0.043 | 0.966 |
| a1 | relative abundance | speciesNiltava macgrigoriae | 0.043 | 1.191 | 0.036 | 0.971 |
| a1 | relative abundance | speciesNiltava rubicoloides | -0.446 | 1.380 | -0.323 | 0.746 |
| a1 | relative abundance | speciesNiltava sp. | -0.138 | 2.104 | -0.065 | 0.948 |
| a1 | relative abundance | speciesNiltava sundara | -0.062 | 1.016 | -0.061 | 0.952 |
| a1 | relative abundance | speciesNyctyornis athertoni | -0.381 | 1.612 | -0.236 | 0.813 |
| a1 | relative abundance | speciesOriolus traillii | -0.381 | 1.612 | -0.236 | 0.813 |
| a1 | relative abundance | speciesOrthotomus cucullatus | -0.162 | 1.651 | -0.098 | 0.922 |
| a1 | relative abundance | speciesOrthotomus sutorius | -0.284 | 2.092 | -0.136 | 0.892 |
| a1 | relative abundance | speciesOtus bakkamoena | -0.351 | 2.003 | -0.175 | 0.861 |
| a1 | relative abundance | speciesOtus sunia | -0.110 | 1.385 | -0.079 | 0.937 |
| a1 | relative abundance | speciesParadoxornis atrosupercilliaris | -0.945 | 1.990 | -0.475 | 0.635 |
| a1 | relative abundance | speciesParadoxornis gularis | -0.469 | 2.116 | -0.222 | 0.825 |
| a1 | relative abundance | speciesParadoxornis nipalensis | 0.323 | 1.789 | 0.181 | 0.857 |
| a1 | relative abundance | speciesParadoxornis ruficeps | 0.217 | 1.248 | 0.174 | 0.862 |
| a1 | relative abundance | speciesParus ater | -0.352 | 2.129 | -0.165 | 0.869 |
| a1 | relative abundance | speciesParus monticolus | -0.151 | 1.188 | -0.127 | 0.899 |
| a1 | relative abundance | speciesParus spilnotus | -0.374 | 2.030 | -0.184 | 0.854 |
| a1 | relative abundance | speciesPasser montanus | -0.945 | 1.990 | -0.475 | 0.635 |
| a1 | relative abundance | speciesPasser rutilans | -0.124 | 2.153 | -0.058 | 0.954 |
| a1 | relative abundance | speciesPellorneum ruficeps | 0.342 | 1.533 | 0.223 | 0.823 |
| a1 | relative abundance | speciesPellorneum sp. | -0.175 | 1.155 | -0.151 | 0.880 |
| a1 | relative abundance | speciesPellorneum tickelli | -0.103 | 1.438 | -0.072 | 0.943 |
| a1 | relative abundance | speciesPericrocotus ethologus | -0.945 | 1.990 | -0.475 | 0.635 |
| a1 | relative abundance | speciesPericrocotus flammeus | -0.326 | 1.791 | -0.182 | 0.856 |
| a1 | relative abundance | speciesPericrocotus roseus | -0.322 | 2.087 | -0.154 | 0.878 |
| a1 | relative abundance | speciesPhoenicurus auroreus | -0.132 | 2.084 | -0.063 | 0.950 |
| a1 | relative abundance | speciesPhylloscopus cantator | -0.446 | 1.380 | -0.323 | 0.746 |
| a1 | relative abundance | speciesPhylloscopus inornatus | -0.345 | 2.527 | -0.136 | 0.892 |
| a1 | relative abundance | speciesPhylloscopus magnirostris | -0.367 | 1.839 | -0.200 | 0.842 |
| a1 | relative abundance | speciesPhylloscopus proregulus | -0.945 | 1.990 | -0.475 | 0.635 |
| a1 | relative abundance | speciesPicus canus | -0.322 | 2.087 | -0.154 | 0.878 |
| a1 | relative abundance | speciesPnoepyga pusilla | -0.322 | 2.087 | -0.154 | 0.878 |
| a1 | relative abundance | speciesPolyplectron bicalcaratum | -0.227 | 1.941 | -0.117 | 0.907 |
| a1 | relative abundance | speciesPomatorhinus ferruginosus | -0.023 | 1.079 | -0.022 | 0.983 |
| a1 | relative abundance | speciesPomatorhinus ochraceiceps | -0.382 | 2.006 | -0.190 | 0.849 |
| a1 | relative abundance | speciesPrunella immaculata | -0.222 | 1.408 | -0.158 | 0.875 |
| a1 | relative abundance | speciesPsarisomus dalhousiae | -0.330 | 1.566 | -0.211 | 0.833 |
| a1 | relative abundance | speciesPseudominla castaneceps | -0.265 | 1.152 | -0.230 | 0.818 |
| a1 | relative abundance | speciesPycnonotus jocosus | 0.087 | 1.350 | 0.064 | 0.949 |
| a1 | relative abundance | speciesPycnonotus leucocephalus | 0.086 | 1.702 | 0.051 | 0.960 |
| a1 | relative abundance | speciesPycnonotus xanthorrous | -0.124 | 2.153 | -0.058 | 0.954 |
| a1 | relative abundance | speciesPyrrhula erythaca | -0.345 | 2.527 | -0.136 | 0.892 |
| a1 | relative abundance | speciesPyrrhula nipalensis | -0.124 | 2.153 | -0.058 | 0.954 |
| a1 | relative abundance | speciesRhipidura albicollis | 0.210 | 1.179 | 0.178 | 0.858 |
| a1 | relative abundance | speciesRhyacornis fuliginosus | -0.138 | 2.104 | -0.065 | 0.948 |
| a1 | relative abundance | speciesRimator malacoptilus | 0.220 | 1.886 | 0.116 | 0.907 |
| a1 | relative abundance | speciesSasia ochracea | 0.109 | 1.150 | 0.094 | 0.925 |
| a1 | relative abundance | speciesSeicercus affinis | -0.703 | 1.510 | -0.465 | 0.642 |
| a1 | relative abundance | speciesSeicercus burkii | -0.945 | 1.990 | -0.475 | 0.635 |
| a1 | relative abundance | speciesSeicercus poliogenys | 0.033 | 1.028 | 0.032 | 0.974 |
| a1 | relative abundance | speciesSeicercus sp. | -0.485 | 1.653 | -0.293 | 0.769 |
| a1 | relative abundance | speciesSeicercus tephrocephalus | -0.252 | 1.516 | -0.166 | 0.868 |
| a1 | relative abundance | speciesSeicercus whistleri | -0.351 | 2.003 | -0.175 | 0.861 |
| a1 | relative abundance | speciesSerilophus lunatus | 0.099 | 1.168 | 0.085 | 0.932 |
| a1 | relative abundance | speciesSitta formosa | 0.218 | 1.360 | 0.160 | 0.873 |
| a1 | relative abundance | speciesSphenocichla humei | 0.370 | 1.807 | 0.205 | 0.838 |
| a1 | relative abundance | speciesSphenocichla roberti | -0.374 | 2.030 | -0.184 | 0.854 |
| a1 | relative abundance | speciesStachyris chrysaea | -0.248 | 1.243 | -0.199 | 0.842 |
| a1 | relative abundance | speciesStachyris nigriceps | 0.260 | 1.033 | 0.251 | 0.801 |
| a1 | relative abundance | speciesStachyris oglei | -0.252 | 1.516 | -0.166 | 0.868 |
| a1 | relative abundance | speciesStachyris ruficeps | 0.152 | 1.426 | 0.107 | 0.915 |
| a1 | relative abundance | speciesStachyris striolata | -0.485 | 1.653 | -0.293 | 0.769 |
| a1 | relative abundance | speciesStrix aluco | -1.406 | 3.062 | -0.459 | 0.646 |
| a1 | relative abundance | speciesTarsiger cyanurus | -0.267 | 1.593 | -0.167 | 0.867 |
| a1 | relative abundance | speciesTerpsiphone paradisi | -0.252 | 1.516 | -0.166 | 0.868 |
| a1 | relative abundance | speciesTesia olivea | 0.004 | 1.387 | 0.003 | 0.998 |
| a1 | relative abundance | speciesTrogopan temminckii | -0.124 | 2.153 | -0.058 | 0.954 |
| a1 | relative abundance | speciesTurdus boulboul | -0.945 | 1.990 | -0.475 | 0.635 |
| a1 | relative abundance | speciesUpupa epops | -0.124 | 2.153 | -0.058 | 0.954 |
| a1 | relative abundance | speciesUrocissa flavirostris | -0.357 | 2.120 | -0.168 | 0.866 |
| a1 | relative abundance | speciesYuhina bakeri | -0.091 | 2.120 | -0.043 | 0.966 |
| a1 | relative abundance | speciesYuhina castaneiceps | -0.252 | 1.516 | -0.166 | 0.868 |
| a1 | relative abundance | speciesYuhina flavicollis | -0.333 | 1.390 | -0.239 | 0.811 |
| a1 | relative abundance | speciesYuhina gularis | -0.124 | 2.153 | -0.058 | 0.954 |
| a1 | relative abundance | speciesYuhina nigrimenta | -0.081 | 1.325 | -0.061 | 0.951 |
| a1 | relative abundance | speciesYuhina zantholeuca | 0.138 | 1.160 | 0.119 | 0.905 |
| a1 | relative abundance | speciesZoothera citrina | -0.485 | 1.653 | -0.293 | 0.769 |
| a1 | relative abundance | speciesZoothera dixoni | -0.352 | 2.821 | -0.125 | 0.901 |
| a1 | relative abundance | localityBabwa | -1.582 | 2.702 | -0.586 | 0.558 |
| a1 | relative abundance | localityGawai | NA | NA | NA | NA |
| a1 | relative abundance | localityGawlai | 0.453 | 3.094 | 0.146 | 0.884 |
| a1 | relative abundance | localityHkamko | -0.636 | 2.461 | -0.259 | 0.796 |
| a1 | relative abundance | localityHtingnan | -0.438 | 3.437 | -0.128 | 0.898 |
| a1 | relative abundance | localityKankiu | 0.505 | 3.539 | 0.143 | 0.886 |
| a1 | relative abundance | localityLonnat | -0.604 | 2.391 | -0.253 | 0.800 |
| a1 | relative abundance | localityMasa | -0.722 | 2.393 | -0.302 | 0.763 |
| a1 | relative abundance | localityMatsatap | 0.171 | 4.118 | 0.041 | 0.967 |
| a1 | relative abundance | localityNagwa | -0.681 | 2.359 | -0.289 | 0.773 |
| a1 | relative abundance | localityNam Tisang river | -0.431 | 3.462 | -0.125 | 0.901 |
| a1 | relative abundance | localityNamTamaiRiver | -0.438 | 2.273 | -0.193 | 0.847 |
| a1 | relative abundance | localityNamTi | -0.462 | 2.307 | -0.200 | 0.841 |
| a1 | relative abundance | localityNaunghkai | 0.580 | 3.261 | 0.178 | 0.859 |
| a1 | relative abundance | localityNaungMung | 0.133 | 2.299 | 0.058 | 0.954 |
| a1 | relative abundance | localityPangnamdim | 0.269 | 3.095 | 0.087 | 0.931 |
| a1 | relative abundance | localityPutao | 0.415 | 3.072 | 0.135 | 0.892 |
| a1 | relative abundance | localityRatnamhti | -0.537 | 2.944 | -0.183 | 0.855 |
| a1 | relative abundance | localitySeinghku | 1.252 | 2.563 | 0.488 | 0.625 |
| a1 | relative abundance | localityShinsanku | -0.572 | 2.458 | -0.233 | 0.816 |
| a1 | relative abundance | localityTahaundam | 0.488 | 3.070 | 0.159 | 0.874 |
| a1 | relative abundance | localityTazundam | 0.317 | 3.191 | 0.099 | 0.921 |
| a1 | relative abundance | localityThalatu | 0.428 | 3.066 | 0.140 | 0.889 |
| a1 | relative abundance | localityURingGa | 0.513 | 3.031 | 0.169 | 0.866 |
| a1 | relative abundance | localityWangsewan | -0.461 | 2.442 | -0.189 | 0.850 |
| a1 | relative abundance | Habitat1989Forest > 1800m | -0.229 | 2.009 | -0.114 | 0.909 |
| a1 | relative abundance | Habitat1989Forest 600-1800m | 0.944 | 1.959 | 0.482 | 0.630 |
| a1 | relative abundance | Habitat1989Ice/glacier | -0.382 | 2.138 | -0.179 | 0.858 |
| a1 | relative abundance | Habitat1989Pine/Rhododendron | -0.651 | 2.637 | -0.247 | 0.805 |
| a1 | relative abundance | Habitat1989Rock/boulder | 1.538 | 3.829 | 0.402 | 0.688 |
| a1 | relative abundance | Habitat1989Secondary forest <600m | NA | NA | NA | NA |
| a1 | relative abundance | Habitat1989Secondary forest 600-1800m | NA | NA | NA | NA |
| a1 | relative abundance | Habitat1989Settlement | NA | NA | NA | NA |
| a1 | relative abundance | Habitat1989Shrub/Bush/Fern | 0.335 | 2.985 | 0.112 | 0.911 |
| a1 | relative abundance | Habitat1989Streambed | NA | NA | NA | NA |
| a1 | relative abundance | Habitat2016Forest > 1800m | 0.486 | 3.530 | 0.138 | 0.891 |
| a1 | relative abundance | Habitat2016Forest 600-1800m | NA | NA | NA | NA |
| a1 | relative abundance | Habitat2016Ice/glacier | -1.061 | 2.809 | -0.378 | 0.706 |
| a1 | relative abundance | Habitat2016Paddyfield | NA | NA | NA | NA |
| a1 | relative abundance | Habitat2016Pine/Rhododendron | 1.143 | 3.059 | 0.373 | 0.709 |
| a1 | relative abundance | Habitat2016Rock/boulder | NA | NA | NA | NA |
| a1 | relative abundance | Habitat2016Secondary forest <600m | NA | NA | NA | NA |
| a1 | relative abundance | Habitat2016Settlement | NA | NA | NA | NA |
| a1 | relative abundance | Habitat2016Shrub/Bush/Fern | NA | NA | NA | NA |
| a1 | relative abundance | Habitat2016Streambed | NA | NA | NA | NA |
| a1 | relative abundance | TypeRecent20012006 | NA | NA | NA | NA |
| a2 | relative abundance | (Intercept) | 0.252 | 0.101 | 2.499 | 0.013 |
| a2 | relative abundance | localityBabwa | -0.016 | 0.107 | -0.150 | 0.881 |
| a2 | relative abundance | localityGawai | -0.041 | 0.202 | -0.203 | 0.839 |
| a2 | relative abundance | localityGawlai | 0.113 | 0.111 | 1.020 | 0.309 |
| a2 | relative abundance | localityHkamko | -0.188 | 0.136 | -1.383 | 0.168 |
| a2 | relative abundance | localityHtingnan | -0.217 | 0.165 | -1.313 | 0.190 |
| a2 | relative abundance | localityKankiu | 0.225 | 0.192 | 1.171 | 0.242 |
| a2 | relative abundance | localityLonnat | -0.164 | 0.130 | -1.261 | 0.208 |
| a2 | relative abundance | localityMasa | -0.169 | 0.128 | -1.320 | 0.188 |
| a2 | relative abundance | localityMatsatap | 0.098 | 0.233 | 0.421 | 0.674 |
| a2 | relative abundance | localityNagwa | -0.169 | 0.129 | -1.313 | 0.190 |
| a2 | relative abundance | localityNam Tisang river | -0.217 | 0.165 | -1.313 | 0.190 |
| a2 | relative abundance | localityNamTamaiRiver | -0.103 | 0.118 | -0.870 | 0.385 |
| a2 | relative abundance | localityNamTi | -0.109 | 0.122 | -0.897 | 0.370 |
| a2 | relative abundance | localityNaunghkai | 0.093 | 0.130 | 0.716 | 0.474 |
| a2 | relative abundance | localityNaungMung | 0.098 | 0.118 | 0.830 | 0.407 |
| a2 | relative abundance | localityPangnamdim | -0.042 | 0.181 | -0.234 | 0.815 |
| a2 | relative abundance | localityPutao | -0.100 | 0.180 | -0.554 | 0.580 |
| a2 | relative abundance | localityRatnamhti | -0.217 | 0.144 | -1.511 | 0.132 |
| a2 | relative abundance | localitySeinghku | 0.084 | 0.092 | 0.908 | 0.365 |
| a2 | relative abundance | localityShinsanku | -0.122 | 0.139 | -0.876 | 0.382 |
| a2 | relative abundance | localityTahaundam | -0.042 | 0.181 | -0.232 | 0.817 |
| a2 | relative abundance | localityTazundam | -0.066 | 0.193 | -0.341 | 0.733 |
| a2 | relative abundance | localityThalatu | -0.005 | 0.167 | -0.029 | 0.977 |
| a2 | relative abundance | localityURingGa | 0.135 | 0.106 | 1.280 | 0.201 |
| a2 | relative abundance | localityWangsewan | -0.122 | 0.139 | -0.876 | 0.382 |
| a2 | relative abundance | Habitat1989Forest > 1800m | 0.054 | 0.104 | 0.518 | 0.605 |
| a2 | relative abundance | Habitat1989Forest 600-1800m | 0.266 | 0.102 | 2.612 | 0.009 |
| a2 | relative abundance | Habitat1989Ice/glacier | 0.067 | 0.126 | 0.536 | 0.592 |
| a2 | relative abundance | Habitat1989Pine/Rhododendron | 0.024 | 0.132 | 0.185 | 0.854 |
| a2 | relative abundance | Habitat1989Rock/boulder | 0.065 | 0.112 | 0.579 | 0.563 |
| a2 | relative abundance | Habitat1989Secondary forest <600m | NA | NA | NA | NA |
| a2 | relative abundance | Habitat1989Secondary forest 600-1800m | NA | NA | NA | NA |
| a2 | relative abundance | Habitat1989Settlement | 0.159 | 0.201 | 0.792 | 0.429 |
| a2 | relative abundance | Habitat1989Shrub/Bush/Fern | -0.049 | 0.153 | -0.319 | 0.750 |
| a2 | relative abundance | Habitat1989Streambed | NA | NA | NA | NA |
| a3 | relative abundance | (Intercept) | 0.305 | 0.026 | 11.840 | <0.001 |
| a3 | relative abundance | localityBabwa | -0.004 | 0.099 | -0.043 | 0.965 |
| a3 | relative abundance | localityGawai | 0.172 | 0.167 | 1.028 | 0.305 |
| a3 | relative abundance | localityGawlai | 0.059 | 0.053 | 1.132 | 0.258 |
| a3 | relative abundance | localityHkamko | 0.025 | 0.072 | 0.347 | 0.729 |
| a3 | relative abundance | localityHtingnan | -0.004 | 0.120 | -0.036 | 0.971 |
| a3 | relative abundance | localityKankiu | 0.172 | 0.167 | 1.028 | 0.305 |
| a3 | relative abundance | localityLonnat | 0.049 | 0.061 | 0.802 | 0.423 |
| a3 | relative abundance | localityMasa | 0.044 | 0.056 | 0.780 | 0.436 |
| a3 | relative abundance | localityMatsatap | -0.004 | 0.167 | -0.026 | 0.980 |
| a3 | relative abundance | localityNagwa | 0.043 | 0.058 | 0.746 | 0.456 |
| a3 | relative abundance | localityNam Tisang river | -0.004 | 0.120 | -0.036 | 0.971 |
| a3 | relative abundance | localityNamTamaiRiver | 0.105 | 0.036 | 2.893 | 0.004 |
| a3 | relative abundance | localityNamTi | 0.104 | 0.039 | 2.688 | 0.008 |
| a3 | relative abundance | localityNaunghkai | 0.040 | 0.086 | 0.459 | 0.646 |
| a3 | relative abundance | localityNaungMung | 0.294 | 0.032 | 9.089 | <0.001 |
| a3 | relative abundance | localityPangnamdim | 0.063 | 0.053 | 1.207 | 0.228 |
| a3 | relative abundance | localityPutao | 0.006 | 0.048 | 0.127 | 0.899 |
| a3 | relative abundance | localityRatnamhti | -0.004 | 0.086 | -0.050 | 0.960 |
| a3 | relative abundance | localitySeinghku | 0.058 | 0.054 | 1.074 | 0.284 |
| a3 | relative abundance | localityShinsanku | 0.091 | 0.078 | 1.165 | 0.245 |
| a3 | relative abundance | localityTahaundam | 0.064 | 0.051 | 1.249 | 0.212 |
| a3 | relative abundance | localityTazundam | 0.040 | 0.086 | 0.459 | 0.646 |
| a3 | relative abundance | localityThalatu | 0.091 | 0.056 | 1.628 | 0.105 |
| a3 | relative abundance | localityURingGa | 0.082 | 0.041 | 2.003 | 0.046 |
| a3 | relative abundance | localityWangsewan | 0.091 | 0.078 | 1.165 | 0.245 |
| a4 | relative abundance | (Intercept) | 0.301 | 0.090 | 3.338 | 0.001 |
| a4 | relative abundance | Habitat1989Forest > 1800m | 0.000 | 0.098 | 0.000 | 1.000 |
| a4 | relative abundance | Habitat1989Forest 600-1800m | 0.164 | 0.091 | 1.801 | 0.073 |
| a4 | relative abundance | Habitat1989Ice/glacier | 0.035 | 0.121 | 0.291 | 0.771 |
| a4 | relative abundance | Habitat1989Pine/Rhododendron | 0.041 | 0.102 | 0.401 | 0.689 |
| a4 | relative abundance | Habitat1989Rock/boulder | 0.013 | 0.102 | 0.123 | 0.902 |
| a4 | relative abundance | Habitat1989Secondary forest <600m | 0.176 | 0.202 | 0.873 | 0.383 |
| a4 | relative abundance | Habitat1989Secondary forest 600-1800m | 0.079 | 0.095 | 0.835 | 0.404 |
| a4 | relative abundance | Habitat1989Settlement | 0.056 | 0.093 | 0.599 | 0.550 |
| a4 | relative abundance | Habitat1989Shrub/Bush/Fern | 0.000 | 0.138 | 0.000 | 1.000 |
| a4 | relative abundance | Habitat1989Streambed | 0.044 | 0.128 | 0.345 | 0.730 |
| a5 | relative abundance | (Intercept) | 0.341 | 0.052 | 6.561 | 0.000 |
| a5 | relative abundance | Habitat2016Forest > 1800m | -0.040 | 0.062 | -0.640 | 0.522 |
| a5 | relative abundance | Habitat2016Forest 600-1800m | 0.121 | 0.053 | 2.273 | 0.024 |
| a5 | relative abundance | Habitat2016Ice/glacier | -0.022 | 0.077 | -0.288 | 0.774 |
| a5 | relative abundance | Habitat2016Paddyfield | -0.031 | 0.066 | -0.471 | 0.638 |
| a5 | relative abundance | Habitat2016Pine/Rhododendron | -0.040 | 0.137 | -0.289 | 0.773 |
| a5 | relative abundance | Habitat2016Rock/boulder | 0.025 | 0.066 | 0.374 | 0.708 |
| a5 | relative abundance | Habitat2016Secondary forest <600m | 0.136 | 0.187 | 0.728 | 0.467 |
| a5 | relative abundance | Habitat2016Settlement | 0.032 | 0.057 | 0.562 | 0.574 |
| a5 | relative abundance | Habitat2016Shrub/Bush/Fern | -0.040 | 0.116 | -0.342 | 0.732 |
| a5 | relative abundance | Habitat2016Streambed | 0.004 | 0.104 | 0.041 | 0.967 |
| a6 | relative abundance | (Intercept) | 0.348 | 0.016 | 21.871 | <0.001 |
| a6 | relative abundance | TypeRecent20012006 | 0.098 | 0.020 | 4.915 | 0.000 |
| a7 | relative abundance | (Intercept) | 0.548 | 0.123 | 4.462 | 0.000 |
| a7 | relative abundance | speciesAbroscopus schisticeps | -0.247 | 0.246 | -1.005 | 0.316 |
| a7 | relative abundance | speciesAccipiter virgatus | -0.247 | 0.194 | -1.271 | 0.205 |
| a7 | relative abundance | speciesAceros nipalensis | -0.247 | 0.246 | -1.005 | 0.316 |
| a7 | relative abundance | speciesAceros undulatus | -0.247 | 0.246 | -1.005 | 0.316 |
| a7 | relative abundance | speciesAcridotheres tristes | -0.247 | 0.246 | -1.005 | 0.316 |
| a7 | relative abundance | speciesActinodura egertoni | -0.247 | 0.194 | -1.271 | 0.205 |
| a7 | relative abundance | speciesActinodura waldeni | -0.247 | 0.246 | -1.005 | 0.316 |
| a7 | relative abundance | speciesAegithalos iouschistos | -0.247 | 0.246 | -1.005 | 0.316 |
| a7 | relative abundance | speciesAethopyga gouldiae | -0.247 | 0.246 | -1.005 | 0.316 |
| a7 | relative abundance | speciesAethopyga nipalensis | -0.247 | 0.194 | -1.271 | 0.205 |
| a7 | relative abundance | speciesAethopyga saturata | -0.103 | 0.144 | -0.717 | 0.474 |
| a7 | relative abundance | speciesAethopyga siparaja | -0.071 | 0.246 | -0.288 | 0.774 |
| a7 | relative abundance | speciesAlcedo athis | -0.247 | 0.246 | -1.005 | 0.316 |
| a7 | relative abundance | speciesAlcedo hercules | -0.247 | 0.194 | -1.271 | 0.205 |
| a7 | relative abundance | speciesAlcippe cinerea | -0.008 | 0.162 | -0.051 | 0.960 |
| a7 | relative abundance | speciesAlcippe cinereiceps | 0.054 | 0.246 | 0.221 | 0.825 |
| a7 | relative abundance | speciesAlcippe morrisonia | 0.067 | 0.150 | 0.447 | 0.656 |
| a7 | relative abundance | speciesAlcippe rufogularis | 0.201 | 0.174 | 1.156 | 0.249 |
| a7 | relative abundance | speciesAlcippe vinipectus | -0.247 | 0.194 | -1.271 | 0.205 |
| a7 | relative abundance | speciesAlophoixus flaveolus | 0.163 | 0.174 | 0.941 | 0.348 |
| a7 | relative abundance | speciesAnthus richardi | -0.247 | 0.194 | -1.271 | 0.205 |
| a7 | relative abundance | speciesAnthus roseatus | -0.247 | 0.246 | -1.005 | 0.316 |
| a7 | relative abundance | speciesArachnothera longirostris | -0.247 | 0.174 | -1.421 | 0.157 |
| a7 | relative abundance | speciesArachnothera magna | -0.072 | 0.155 | -0.462 | 0.645 |
| a7 | relative abundance | speciesArborophila atrogularis | -0.247 | 0.246 | -1.005 | 0.316 |
| a7 | relative abundance | speciesArborophila rufogularis | -0.247 | 0.246 | -1.005 | 0.316 |
| a7 | relative abundance | speciesBlythipicus pyrrhotis | -0.247 | 0.162 | -1.519 | 0.131 |
| a7 | relative abundance | speciesBrachypteryx hyperthyra | -0.071 | 0.246 | -0.288 | 0.774 |
| a7 | relative abundance | speciesBrachypteryx leucophris | -0.247 | 0.194 | -1.271 | 0.205 |
| a7 | relative abundance | speciesBrachypteryx sp. | 0.054 | 0.246 | 0.221 | 0.825 |
| a7 | relative abundance | speciesCeryle lugubris | -0.247 | 0.194 | -1.271 | 0.205 |
| a7 | relative abundance | speciesChaimarrornis leucocephalus | -0.247 | 0.246 | -1.005 | 0.316 |
| a7 | relative abundance | speciesChalcophaps indica | -0.071 | 0.246 | -0.288 | 0.774 |
| a7 | relative abundance | speciesCheliodorhynx hypoxantha | -0.247 | 0.246 | -1.005 | 0.316 |
| a7 | relative abundance | speciesChloropsis hardwickii | -0.247 | 0.246 | -1.005 | 0.316 |
| a7 | relative abundance | speciesCinclidium leucurum | -0.247 | 0.246 | -1.005 | 0.316 |
| a7 | relative abundance | speciesCinclus pallasii | -0.247 | 0.246 | -1.005 | 0.316 |
| a7 | relative abundance | speciesCissa chinensis | -0.212 | 0.155 | -1.362 | 0.175 |
| a7 | relative abundance | speciesCissa hypoleuca | -0.071 | 0.246 | -0.288 | 0.774 |
| a7 | relative abundance | speciesCollocalia brevirostris | -0.159 | 0.194 | -0.818 | 0.415 |
| a7 | relative abundance | speciesCoracina melaschistos | -0.071 | 0.246 | -0.288 | 0.774 |
| a7 | relative abundance | speciesCorvus macrorhynchus | -0.247 | 0.246 | -1.005 | 0.316 |
| a7 | relative abundance | speciesCulicicapa ceylonensis | -0.065 | 0.174 | -0.377 | 0.707 |
| a7 | relative abundance | speciesCyornis hainanus | -0.247 | 0.246 | -1.005 | 0.316 |
| a7 | relative abundance | speciesCyornis sp. | -0.247 | 0.246 | -1.005 | 0.316 |
| a7 | relative abundance | speciesDelichon dasypus | -0.247 | 0.246 | -1.005 | 0.316 |
| a7 | relative abundance | speciesDendrocitta frontalis | -0.039 | 0.162 | -0.243 | 0.808 |
| a7 | relative abundance | speciesDendrocopus cathparius | -0.247 | 0.246 | -1.005 | 0.316 |
| a7 | relative abundance | speciesDicrurus aeneus | -0.247 | 0.246 | -1.005 | 0.316 |
| a7 | relative abundance | speciesDicrurus paradiseus | -0.247 | 0.246 | -1.005 | 0.316 |
| a7 | relative abundance | speciesDicrurus remifer | -0.247 | 0.246 | -1.005 | 0.316 |
| a7 | relative abundance | speciesEnicurus leuschenaulti | -0.247 | 0.246 | -1.005 | 0.316 |
| a7 | relative abundance | speciesEnicurus maculatus | -0.247 | 0.246 | -1.005 | 0.316 |
| a7 | relative abundance | speciesEnicurus schistaceus | -0.247 | 0.246 | -1.005 | 0.316 |
| a7 | relative abundance | speciesEnicurus scouleri | -0.247 | 0.246 | -1.005 | 0.316 |
| a7 | relative abundance | speciesEurystomus orientalis | 0.054 | 0.246 | 0.221 | 0.825 |
| a7 | relative abundance | speciesFicedula hyperythra | -0.029 | 0.174 | -0.167 | 0.867 |
| a7 | relative abundance | speciesFicedula monileger | -0.091 | 0.147 | -0.618 | 0.537 |
| a7 | relative abundance | speciesFicedula sapphira | -0.247 | 0.194 | -1.271 | 0.205 |
| a7 | relative abundance | speciesFicedula sp. | -0.247 | 0.246 | -1.005 | 0.316 |
| a7 | relative abundance | speciesFicedula strophiata | -0.130 | 0.150 | -0.866 | 0.387 |
| a7 | relative abundance | speciesFicedula tricolor | -0.247 | 0.246 | -1.005 | 0.316 |
| a7 | relative abundance | speciesGarrulax affinis | -0.247 | 0.162 | -1.519 | 0.131 |
| a7 | relative abundance | speciesGarrulax erythrocephalus | -0.114 | 0.174 | -0.657 | 0.512 |
| a7 | relative abundance | speciesGarrulax gularis | -0.055 | 0.174 | -0.319 | 0.750 |
| a7 | relative abundance | speciesGarrulax leucolophus | -0.008 | 0.194 | -0.042 | 0.966 |
| a7 | relative abundance | speciesGarrulax monileger | -0.159 | 0.194 | -0.818 | 0.415 |
| a7 | relative abundance | speciesGarrulax nuchalis | -0.203 | 0.162 | -1.248 | 0.214 |
| a7 | relative abundance | speciesGarrulax ruficollis | -0.188 | 0.174 | -1.083 | 0.280 |
| a7 | relative abundance | speciesGarrulax rufogularis | -0.048 | 0.194 | -0.246 | 0.806 |
| a7 | relative abundance | speciesGarrulax squamatus | -0.071 | 0.246 | -0.288 | 0.774 |
| a7 | relative abundance | speciesGarrulax striatus | -0.046 | 0.174 | -0.265 | 0.791 |
| a7 | relative abundance | speciesGarrulax subunicolor | -0.247 | 0.194 | -1.271 | 0.205 |
| a7 | relative abundance | speciesGecinulus grantia | -0.247 | 0.246 | -1.005 | 0.316 |
| a7 | relative abundance | speciesGlaucidium brodiei | -0.159 | 0.194 | -0.818 | 0.415 |
| a7 | relative abundance | speciesGlaucidium cuculoides | -0.247 | 0.194 | -1.271 | 0.205 |
| a7 | relative abundance | speciesHarpactes erythrocephalus | -0.008 | 0.194 | -0.042 | 0.966 |
| a7 | relative abundance | speciesHemipus picatus | -0.247 | 0.246 | -1.005 | 0.316 |
| a7 | relative abundance | speciesHemixos flavala | -0.159 | 0.194 | -0.818 | 0.415 |
| a7 | relative abundance | speciesHeterophasia picaoides | -0.129 | 0.174 | -0.745 | 0.457 |
| a7 | relative abundance | speciesHeterophasia pulchella | -0.114 | 0.174 | -0.657 | 0.512 |
| a7 | relative abundance | speciesHirundo rustica | -0.071 | 0.246 | -0.288 | 0.774 |
| a7 | relative abundance | speciesHirundo striolata | -0.247 | 0.246 | -1.005 | 0.316 |
| a7 | relative abundance | speciesHypsipetes mcclelandi | -0.188 | 0.174 | -1.083 | 0.280 |
| a7 | relative abundance | speciesIthaginis cruentus | -0.159 | 0.194 | -0.818 | 0.415 |
| a7 | relative abundance | speciesJabouilleia naungmungensis | -0.048 | 0.194 | -0.246 | 0.806 |
| a7 | relative abundance | speciesJynx torquilla | 0.054 | 0.246 | 0.221 | 0.825 |
| a7 | relative abundance | speciesLanius schach | -0.247 | 0.246 | -1.005 | 0.316 |
| a7 | relative abundance | speciesLeiothrix argentauris | -0.083 | 0.162 | -0.514 | 0.608 |
| a7 | relative abundance | speciesLeiothrix lutea | -0.159 | 0.194 | -0.818 | 0.415 |
| a7 | relative abundance | speciesLeucosticte nemoricola | -0.247 | 0.246 | -1.005 | 0.316 |
| a7 | relative abundance | speciesLiocichla phoenicea | -0.247 | 0.194 | -1.271 | 0.205 |
| a7 | relative abundance | speciesLophophorus impejanus | -0.247 | 0.246 | -1.005 | 0.316 |
| a7 | relative abundance | speciesLophophorus sclateri | -0.247 | 0.246 | -1.005 | 0.316 |
| a7 | relative abundance | speciesLophura leucomelanus | -0.247 | 0.174 | -1.421 | 0.157 |
| a7 | relative abundance | speciesMacropygia unchall | -0.247 | 0.246 | -1.005 | 0.316 |
| a7 | relative abundance | speciesMalacocincla abbotti | -0.247 | 0.246 | -1.005 | 0.316 |
| a7 | relative abundance | speciesMegalaima asiatica | -0.071 | 0.246 | -0.288 | 0.774 |
| a7 | relative abundance | speciesMegalaima franklinii | 0.151 | 0.246 | 0.616 | 0.539 |
| a7 | relative abundance | speciesMegalaima virens | -0.247 | 0.194 | -1.271 | 0.205 |
| a7 | relative abundance | speciesMelanochlora sultanea | -0.247 | 0.246 | -1.005 | 0.316 |
| a7 | relative abundance | speciesMicropternus brachyurus | -0.247 | 0.246 | -1.005 | 0.316 |
| a7 | relative abundance | speciesMinla ignotincta | -0.247 | 0.246 | -1.005 | 0.316 |
| a7 | relative abundance | speciesMotacilla alba | 0.054 | 0.246 | 0.221 | 0.825 |
| a7 | relative abundance | speciesMotacilla citriola | -0.247 | 0.246 | -1.005 | 0.316 |
| a7 | relative abundance | speciesMuscicapa sibirica | -0.247 | 0.246 | -1.005 | 0.316 |
| a7 | relative abundance | speciesMycerobas affinis | -0.247 | 0.246 | -1.005 | 0.316 |
| a7 | relative abundance | speciesMycerobas carnipes | -0.247 | 0.246 | -1.005 | 0.316 |
| a7 | relative abundance | speciesMyiomela leucura | 0.151 | 0.246 | 0.616 | 0.539 |
| a7 | relative abundance | speciesMyophonus caeruleus | -0.247 | 0.246 | -1.005 | 0.316 |
| a7 | relative abundance | speciesNapothera brevicaudata | 0.080 | 0.194 | 0.411 | 0.681 |
| a7 | relative abundance | speciesNapothera epilepidota | -0.007 | 0.174 | -0.039 | 0.969 |
| a7 | relative abundance | speciesNiltava grandis | -0.131 | 0.142 | -0.924 | 0.357 |
| a7 | relative abundance | speciesNiltava macgrigoriae | 0.080 | 0.194 | 0.411 | 0.681 |
| a7 | relative abundance | speciesNiltava rubicoloides | -0.159 | 0.194 | -0.818 | 0.415 |
| a7 | relative abundance | speciesNiltava sp. | -0.247 | 0.246 | -1.005 | 0.316 |
| a7 | relative abundance | speciesNiltava sundara | -0.147 | 0.147 | -1.001 | 0.318 |
| a7 | relative abundance | speciesNyctyornis athertoni | -0.247 | 0.194 | -1.271 | 0.205 |
| a7 | relative abundance | speciesOriolus traillii | -0.247 | 0.194 | -1.271 | 0.205 |
| a7 | relative abundance | speciesOrthotomus cucullatus | -0.247 | 0.194 | -1.271 | 0.205 |
| a7 | relative abundance | speciesOrthotomus sutorius | -0.247 | 0.246 | -1.005 | 0.316 |
| a7 | relative abundance | speciesOtus bakkamoena | -0.247 | 0.246 | -1.005 | 0.316 |
| a7 | relative abundance | speciesOtus sunia | -0.159 | 0.194 | -0.818 | 0.415 |
| a7 | relative abundance | speciesParadoxornis atrosupercilliaris | -0.247 | 0.246 | -1.005 | 0.316 |
| a7 | relative abundance | speciesParadoxornis gularis | -0.247 | 0.246 | -1.005 | 0.316 |
| a7 | relative abundance | speciesParadoxornis nipalensis | -0.071 | 0.246 | -0.288 | 0.774 |
| a7 | relative abundance | speciesParadoxornis ruficeps | -0.008 | 0.194 | -0.042 | 0.966 |
| a7 | relative abundance | speciesParus ater | -0.247 | 0.194 | -1.271 | 0.205 |
| a7 | relative abundance | speciesParus monticolus | -0.212 | 0.155 | -1.362 | 0.175 |
| a7 | relative abundance | speciesParus spilnotus | -0.247 | 0.246 | -1.005 | 0.316 |
| a7 | relative abundance | speciesPasser montanus | -0.247 | 0.246 | -1.005 | 0.316 |
| a7 | relative abundance | speciesPasser rutilans | -0.247 | 0.246 | -1.005 | 0.316 |
| a7 | relative abundance | speciesPellorneum ruficeps | 0.054 | 0.246 | 0.221 | 0.825 |
| a7 | relative abundance | speciesPellorneum sp. | -0.088 | 0.174 | -0.505 | 0.614 |
| a7 | relative abundance | speciesPellorneum tickelli | 0.151 | 0.246 | 0.616 | 0.539 |
| a7 | relative abundance | speciesPericrocotus ethologus | -0.159 | 0.194 | -0.818 | 0.415 |
| a7 | relative abundance | speciesPericrocotus flammeus | -0.247 | 0.194 | -1.271 | 0.205 |
| a7 | relative abundance | speciesPericrocotus roseus | -0.247 | 0.246 | -1.005 | 0.316 |
| a7 | relative abundance | speciesPhoenicurus auroreus | -0.247 | 0.246 | -1.005 | 0.316 |
| a7 | relative abundance | speciesPhoenicurus frontalis | -0.247 | 0.246 | -1.005 | 0.316 |
| a7 | relative abundance | speciesPhylloscopus cantator | -0.159 | 0.194 | -0.818 | 0.415 |
| a7 | relative abundance | speciesPhylloscopus inornatus | -0.247 | 0.246 | -1.005 | 0.316 |
| a7 | relative abundance | speciesPhylloscopus magnirostris | -0.247 | 0.194 | -1.271 | 0.205 |
| a7 | relative abundance | speciesPhylloscopus proregulus | -0.247 | 0.246 | -1.005 | 0.316 |
| a7 | relative abundance | speciesPicus canus | -0.247 | 0.246 | -1.005 | 0.316 |
| a7 | relative abundance | speciesPnoepyga pusilla | -0.247 | 0.246 | -1.005 | 0.316 |
| a7 | relative abundance | speciesPolyplectron bicalcaratum | -0.247 | 0.194 | -1.271 | 0.205 |
| a7 | relative abundance | speciesPomatorhinus ferruginosus | -0.067 | 0.162 | -0.411 | 0.682 |
| a7 | relative abundance | speciesPomatorhinus ochraceiceps | -0.247 | 0.246 | -1.005 | 0.316 |
| a7 | relative abundance | speciesPrunella immaculata | -0.247 | 0.174 | -1.421 | 0.157 |
| a7 | relative abundance | speciesPsarisomus dalhousiae | -0.247 | 0.194 | -1.271 | 0.205 |
| a7 | relative abundance | speciesPseudominla castaneceps | -0.172 | 0.162 | -1.056 | 0.292 |
| a7 | relative abundance | speciesPycnonotus jocosus | 0.113 | 0.194 | 0.584 | 0.560 |
| a7 | relative abundance | speciesPycnonotus leucocephalus | -0.071 | 0.246 | -0.288 | 0.774 |
| a7 | relative abundance | speciesPycnonotus xanthorrous | -0.247 | 0.246 | -1.005 | 0.316 |
| a7 | relative abundance | speciesPyrrhula erythaca | -0.247 | 0.246 | -1.005 | 0.316 |
| a7 | relative abundance | speciesPyrrhula nipalensis | -0.247 | 0.246 | -1.005 | 0.316 |
| a7 | relative abundance | speciesRhipidura albicollis | 0.142 | 0.194 | 0.733 | 0.465 |
| a7 | relative abundance | speciesRhyacornis fuliginosus | -0.247 | 0.246 | -1.005 | 0.316 |
| a7 | relative abundance | speciesRimator malacoptilus | -0.071 | 0.246 | -0.288 | 0.774 |
| a7 | relative abundance | speciesSasia ochracea | 0.013 | 0.174 | 0.073 | 0.942 |
| a7 | relative abundance | speciesSeicercus affinis | -0.247 | 0.194 | -1.271 | 0.205 |
| a7 | relative abundance | speciesSeicercus burkii | -0.247 | 0.246 | -1.005 | 0.316 |
| a7 | relative abundance | speciesSeicercus poliogenys | -0.056 | 0.155 | -0.360 | 0.719 |
| a7 | relative abundance | speciesSeicercus sp. | -0.071 | 0.246 | -0.288 | 0.774 |
| a7 | relative abundance | speciesSeicercus tephrocephalus | 0.054 | 0.246 | 0.221 | 0.825 |
| a7 | relative abundance | speciesSeicercus whistleri | -0.247 | 0.246 | -1.005 | 0.316 |
| a7 | relative abundance | speciesSerilophus lunatus | 0.123 | 0.194 | 0.636 | 0.526 |
| a7 | relative abundance | speciesSitta formosa | -0.071 | 0.194 | -0.364 | 0.716 |
| a7 | relative abundance | speciesSphenocichla humei | -0.071 | 0.246 | -0.288 | 0.774 |
| a7 | relative abundance | speciesSphenocichla roberti | -0.247 | 0.194 | -1.271 | 0.205 |
| a7 | relative abundance | speciesStachyris chrysaea | -0.146 | 0.174 | -0.843 | 0.400 |
| a7 | relative abundance | speciesStachyris nigriceps | 0.089 | 0.162 | 0.545 | 0.587 |
| a7 | relative abundance | speciesStachyris oglei | 0.054 | 0.246 | 0.221 | 0.825 |
| a7 | relative abundance | speciesStachyris ruficeps | -0.096 | 0.194 | -0.496 | 0.621 |
| a7 | relative abundance | speciesStachyris striolata | -0.071 | 0.246 | -0.288 | 0.774 |
| a7 | relative abundance | speciesStrix aluco | -0.247 | 0.246 | -1.005 | 0.316 |
| a7 | relative abundance | speciesTarsiger cyanurus | -0.247 | 0.194 | -1.271 | 0.205 |
| a7 | relative abundance | speciesTerpsiphone paradisi | 0.054 | 0.246 | 0.221 | 0.825 |
| a7 | relative abundance | speciesTesia olivea | 0.230 | 0.246 | 0.938 | 0.350 |
| a7 | relative abundance | speciesTrogopan temminckii | -0.247 | 0.246 | -1.005 | 0.316 |
| a7 | relative abundance | speciesTurdus boulboul | -0.247 | 0.246 | -1.005 | 0.316 |
| a7 | relative abundance | speciesUpupa epops | -0.247 | 0.246 | -1.005 | 0.316 |
| a7 | relative abundance | speciesUrocissa flavirostris | -0.247 | 0.246 | -1.005 | 0.316 |
| a7 | relative abundance | speciesYuhina bakeri | -0.247 | 0.246 | -1.005 | 0.316 |
| a7 | relative abundance | speciesYuhina castaneiceps | 0.054 | 0.246 | 0.221 | 0.825 |
| a7 | relative abundance | speciesYuhina flavicollis | -0.247 | 0.174 | -1.421 | 0.157 |
| a7 | relative abundance | speciesYuhina gularis | -0.247 | 0.246 | -1.005 | 0.316 |
| a7 | relative abundance | speciesYuhina nigrimenta | -0.188 | 0.174 | -1.083 | 0.280 |
| a7 | relative abundance | speciesYuhina zantholeuca | 0.142 | 0.194 | 0.733 | 0.465 |
| a7 | relative abundance | speciesZoothera citrina | -0.071 | 0.246 | -0.288 | 0.774 |
| a7 | relative abundance | speciesZoothera dixoni | -0.071 | 0.246 | -0.288 | 0.774 |


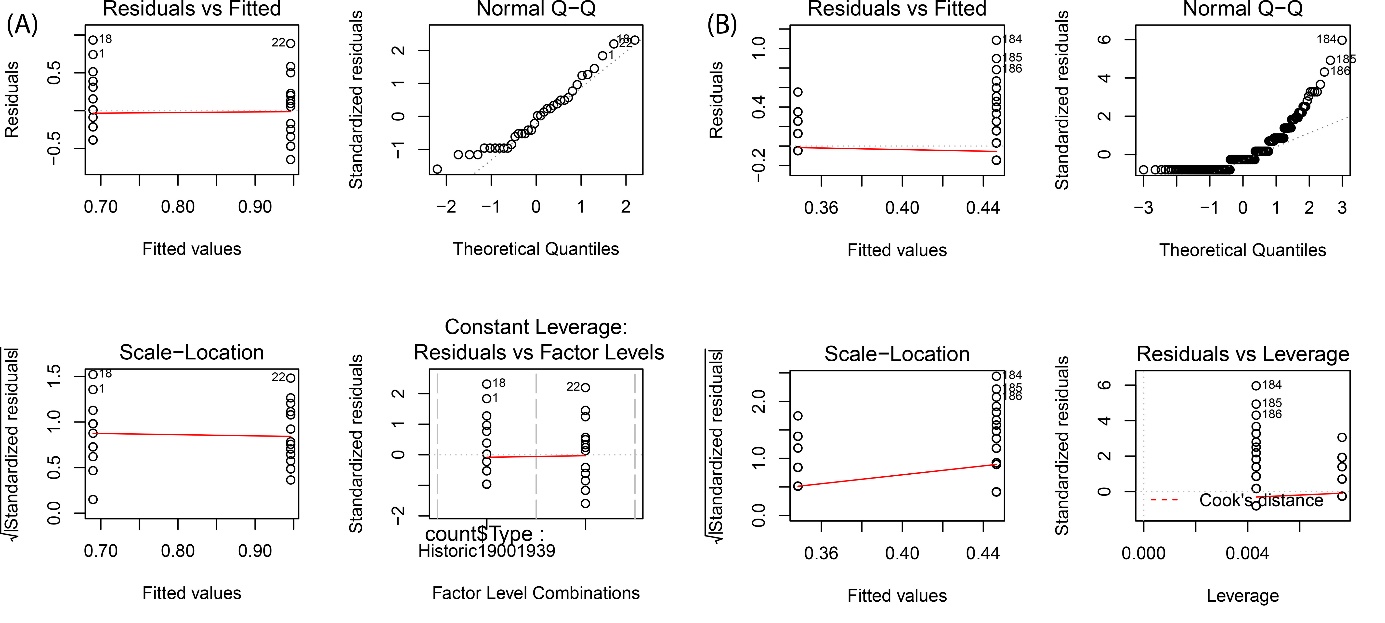
**Figure** **S1.** Residuals for species numbers **(A)** and relative abundance **(B)** of birds in the Hkakabo Razi Landscape pre-1940 and post-2000 with similar methods and effort established.

**
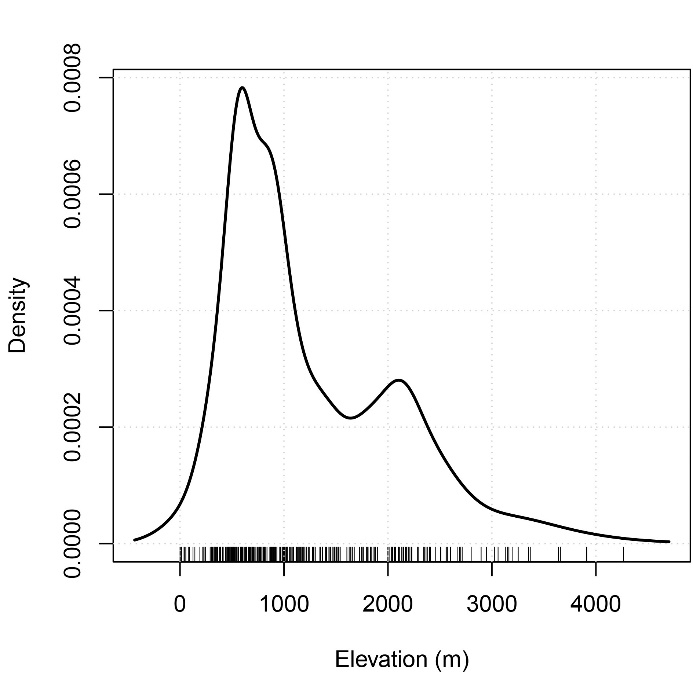
Figure S2.** Density of bird records in respect to elevation of 365 species known from the Hkakabo Razi Landscape. Included are all records with elevational indications (2,999 individuals accounted for). Gaussian density estimate with 1.5 times multiplied bandwidth to smooth graph and based on mean elevation per species (adapted and amended from data in Renner, et al. ^17^).

**
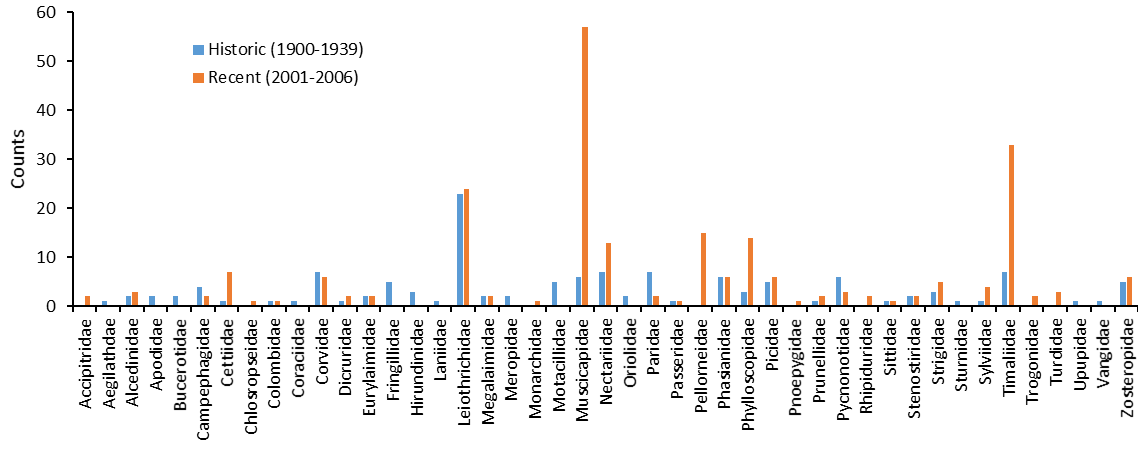
**

**Figure S3.** Families after “The Tree of Live” (alphabetical order) of the species encountered pre-1940 and post-2000 in the Hkakabo Razi Landscape. Counts indicate the number of species per family encountered.

# References cited in Online Supporting Information

1 CCKP. *Climate Change Knowledge Portal - Putao data*. (The World Bank,, 2015).

2 Brown, G. E. R. G. *Upper Chindwin District Vol. A*. Vol. A 1-91 (Government Printing Office, 1960).

3 George, E. C. S. *Ruby Mines District Vol. A*. Vol. A (Government Printing and Stationary, 1915).

4 Grantham, S. & Maung Lat. *Yamethin District Vol. B*. Vol. B 61 (Government Printing and Stationary, 1924).

5 Harrington, H. H. *The birds of Burma*. (Rangoon Gazette Press 1909).

6 Hertz, W. A. *Myitkyina District Vol. A*. Vol. A (Government Printing and Stationary, 1912).

7 N.N. *Mandalay District Vol. B*. Vol. B 1-62 (Government Printing Office, 1925).

8 N.N. *Ruby Mines District Vol. B*. Vol. B 1-64 (Government Printing and Stationary, 1913).

9 Scott, J. G. & Hardiman, A. P. *Gazetteer of Upper Burma and the Shan States*. Vol. 1 (Government Publication, 1900).

10 Searle, H. F. *Mandalay District Vol. A*. Vol. A 1-257 (Government Printing Office, 1928).

11 Spearman, H. R. *The British Burma Gazetteer*. (Printed at the Government Press, 1880).

12 Stewart, J. A., Neild, R. & Searle, H. F. *Kyauksè District Vol. A*. Vol. A (Government Printing and Stationery, 1925).

13 Tydd, W. B. *Sandoway District Vol. A*. Vol. A 35 (Government Printing and Stationery, 1962).

14 Watson, J. W. *Myitkyina District and Putao District Vol. B*. Vol. B 1-67 (Supdt., Government Printing And Stationery, Burma, 1925).

15 Wilkie, R. S. *Yamethin District Vol. A*. Vol. A 1-184 (Government Printing and Stationary, 1934).

16 Ticehurst, D. C. B. IV.-On the Birds of Northern Burma-Part I. *Ibis* **80**, 65-102, doi:10.1111/j.1474-919X.1938.tb00185.x (2008).

17 Renner, S. C. *et al.* Avifauna of the Southeastern Himalayan Mountains and neighboring Myanmar hill country. *Bonn Zoological Bulletin - Supplementum* **62**, 1-75 (2015).

18 Renner, S. & van Hoesel, W. Ecological and functional traits in 99 bird species over a large-scale gradient in Germany. *Data* **2**, 12, doi:10.3390/data2020012 (2017).

19 Suarez-Rubio, M. *et al.* Last of their kind: The Hkakabo Razi Landscape as a global precedence for high integrity of forests. *Scientific reports* (in review).
